# RLPredictiOme, a Machine Learning-Derived Method for High-Throughput Prediction of Plant Receptor-like Proteins, Reveals Novel Classes of Transmembrane Receptors

**DOI:** 10.3390/ijms232012176

**Published:** 2022-10-12

**Authors:** Jose Cleydson F. Silva, Marco Aurélio Ferreira, Thales F. M. Carvalho, Fabyano F. Silva, Sabrina de A. Silveira, Sergio H. Brommonschenkel, Elizabeth P. B. Fontes

**Affiliations:** 1 National Institute of Science and Technology in Plant-Pest Interactions, Bioagro, Viçosa 36570-900, Brazil; 2 Departament of Biochemistry and Molecular Biology, Universidade Federal de Viçosa, Viçosa 36570-900, Brazil; 3 Institute of Engineering, Science and Technology, Universidade Federal dos Vales do Jequitinhonha e Mucuri, Janaúba 39447-814, Brazil; 4 Departament of Animal Science, Universidade Federal de Viçosa, Viçosa 36570-900, Brazil; 5 Department of Computer Science, Universidade Federal de Viçosa, Viçosa 36570-900, Brazil; 6 Plant Pathology Department/Bioagro, Universidade Federal de Viçosa, Viçosa 36570-900, Brazil

**Keywords:** RLPredictiOme, probable lipid transfer (PLT)-RLP, plastocyanin-like-RLP, ring finger-RLP, glycosyl-hydrolase-RLP, glycerophosphoryldiester phosphodiesterase (GDPD GDPDL)-RLP, receptor-like protein kinases, receptor-like proteins

## Abstract

Cell surface receptors play essential roles in perceiving and processing external and internal signals at the cell surface of plants and animals. The receptor-like protein kinases (RLK) and receptor-like proteins (RLPs), two major classes of proteins with membrane receptor configuration, play a crucial role in plant development and disease defense. Although RLPs and RLKs share a similar single-pass transmembrane configuration, RLPs harbor short divergent C-terminal regions instead of the conserved kinase domain of RLKs. This RLP receptor structural design precludes sequence comparison algorithms from being used for high-throughput predictions of the RLP family in plant genomes, as has been extensively performed for RLK superfamily predictions. Here, we developed the RLPredictiOme, implemented with machine learning models in combination with Bayesian inference, capable of predicting RLP subfamilies in plant genomes. The ML models were simultaneously trained using six types of features, along with three stages to distinguish RLPs from non-RLPs (NRLPs), RLPs from RLKs, and classify new subfamilies of RLPs in plants. The ML models achieved high accuracy, precision, sensitivity, and specificity for predicting RLPs with relatively high probability ranging from 0.79 to 0.99. The prediction of the method was assessed with three datasets, two of which contained leucine-rich repeats (LRR)-RLPs from Arabidopsis and rice, and the last one consisted of the complete set of previously described Arabidopsis RLPs. In these validation tests, more than 90% of known RLPs were correctly predicted via RLPredictiOme. In addition to predicting previously characterized RLPs, RLPredictiOme uncovered new RLP subfamilies in the Arabidopsis genome. These include probable lipid transfer (PLT)-RLP, plastocyanin-like-RLP, ring finger-RLP, glycosyl-hydrolase-RLP, and glycerophosphoryldiester phosphodiesterase (GDPD, GDPDL)-RLP subfamilies, yet to be characterized. Compared to the only Arabidopsis GDPDL-RLK, molecular evolution studies confirmed that the ectodomain of GDPDL-RLPs might have undergone a purifying selection with a predominance of synonymous substitutions. Expression analyses revealed that predicted GDPGL-RLPs display a basal expression level and respond to developmental and biotic signals. The results of these biological assays indicate that these subfamily members have maintained functional domains during evolution and may play relevant roles in development and plant defense. Therefore, RLPredictiOme provides a framework for genome-wide surveys of the RLP superfamily as a foundation to rationalize functional studies of surface receptors and their relationships with different biological processes.

## 1. Introduction

The capacity to transiently regulate cellular processes in response to external environmental signals is crucial to all living organisms. While the downstream regulatory events in a signaling cascade can involve biochemical modifications, including protein phosphorylation, ligand binding, and allosteric regulation, as well as changes in the transcription/translation profiles, the initial sensing event is predominantly mediated by membrane receptors. In plants, two major classes of proteins with membrane receptor structural configuration co-exist, namely receptor-like kinases (RLK) and receptor-like proteins (RLP) [1,2]. The receptor-like kinases comprise a large family with more than 420 family members in Arabidopsis [3]. These transmembrane receptors harbor a divergent extracellular domain (ectodomain, ECD) at the N-terminal region, followed by a transmembrane segment (TM) and a C-terminal cytoplasmic signaling domain. This configuration of a single-pass transmembrane kinase receptor invokes a mechanism of ligand binding-induced homo or hetero oligomerization of RLKs as the essential early event for signaling and transducing from the receptor, similarly to the receptor-tyrosine kinases (RTK) of mammalian cells [4,5]. In this scenario, ECD is the stimulus-sensing, ligand recognition domain that induces multimerization, and the kinase domain functions as the phosphorylation-dependent transducing module that relays the signal intracellularly.

Phylogenetic analyses based on the RLK kinase domains organized their ectodomain into clusters of conserved motifs and classified the RLKs into 15 subfamilies. Among them, the leucine-rich repeat (LRR)-RLK subfamily is further subdivided into 13 subfamilies (LRRI-XIII) according to the LRR motif organization ranging from 3 to 26 LRRs [6,7]. The RLK family size has been determined in other plant species, which revealed even larger RLK gene families in the genome of soybean, rice, and tomato [3,8,9,10]. The complexity of the RLK superfamily may reflect the intricate coordination of plant responses to external signals during plant development and interactions with the biotic and abiotic environment. Accordingly, several RLKs have been functionally characterized in development, environmental stresses, and plant defenses (for more details, see references [11,12,13,14,15,16,17,18,19,20,21,22]).

RLKs are also involved in plant immunity and function as pattern recognition receptors (PRRs), which perceive pathogen-associated molecular patterns (PAMPs) or damage-associated molecular patterns (DAMPs) presented, respectively, by pathogens and plants during infection. Interaction of PRRs with PAMPs/DAMPs initiates PAMP-triggered immunity (PTI), the first layer of the innate immune system in plants [23]. Many examples of leucine-rich repeat receptor-like kinases (LRR-RLKs) have been functionally characterized as PRRs (for more details, see references [24,25,26,27,28,29,30,31,32,33,34,35,36,37,38,39,40,41,42]).

The second class of plant transmembrane proteins, RLPs, are built into an N-terminal extracellular domain, which shares similar motifs with RLK ectodomains, an internal single transmembrane segment followed by a short cytoplasmic domain that lacks a transducing-kinase domain [23]. RLPs are structurally similar to Toll-like receptors (TLRs) involved in mammalian immunity, which also contain a leucine-rich repeat ectodomain and a short cytoplasmic tail [5]. The RLP configuration poses a higher degree of complexity for signaling as they depend on heterodimerization with RLKs or association with receptor-like cytoplasmic kinases (RLCK) for transducing a stimulus from the receptor. Accordingly, the leucine-rich repeat receptor-like protein (LRR-RLP) TOO MANY MOUTHS (TMM) forms complexes with LRR-RLKs ERECTA and ERECTA-LIKE 1 (ERL1) to perceive the EPIDERMAL PATTERNING FACTOR 1 (EPF1) and EPF2 peptides for the regulation of stomatal patterning [43], and CLAVATA2 RLP is required for the stability of CLAVATA1 (CLV1) RLK [44]. Likewise, lysine motif (LysM)-RLPs, LYSIN-MOTIF 1 (LYM1), and LYM3 associate with the LysM-RLK CERK1 (CHITIN ELICITOR RECEPTOR KINASE 1) to recognize bacterial peptidoglycans [45], and the LRR-RLP RLP23 forms a complex with the LRR-RLK SUPPRESSOR OF BIR1-1 (SOBIR1) that recognizes NECROSIS- AND ETHYLENE-INDUCING PEPTIDE 1 (NEP1)-LIKE PROTEINS (NLPs) to trigger PTI signaling [46]. In addition to these Arabidopsis RLPs, the first characterized RLP, Cf-9, was identified in tomato plants as an LRR-RLP and has been shown to trigger effector-triggered immunity (ETI)-like signaling, elicited specifically by the *Cladosporium fulvum* Av9 effector [47]. The tomato LRR-RLP Cf-4 is also required for resistance to *C. fulvum* expressing the Avr4 gene [48]. Cf-9 and Cf-4 associate with the RLKs SOBIR1 AND BRI1-ASSOCIATED KINASE 1 to initiate receptor endocytosis and plant immunity [49]. Likewise, *N. benthamiana* LRR-RLP RESPONSE TO XEG1 (RXEG1), which recognizes the glycoside hydrolase 12 protein XEG1, and RLP RE02 (Response to VmE02) forms a complex with BAK1 and SOBIR1 to transduce the XEG1- and VmE02- induced defense signals, respectively [50,51]. The rice RLP, OsRLP1, also interacts with OsSOBIR1 to induce immune responses against viral infection [52].

Although some progress has been reached in characterizing RLPs, a biological function has been assigned to only a few plant RLPs, despite their conceptual relevance in cell signaling events. While 15 RLK subfamilies with distinct ECD have been detected in Arabidopsis, only three Arabidopsis RLP subfamilies have been identified based on single-gene identification and functional studies [2]. The only genome-wide study of RLPs was restricted to the LRR-RLP subfamily [53]. In the case of RLKs, the successful identification and organization of the superfamily in different subfamilies relied on methods that use algorithms, such as BLAST and hidden Markov models (HMM), to perform searches for sequence alignments of conserved regions. One possible explanation for the poor characterization of RLPs may be the difficulty of assigning members to this family based on sequence comparison, as they lack the conserved C-terminal serine/threonine kinase domain, restricting the prediction of novel RLPs. In addition to requiring RLPs to be associated with a kinase domain-containing receptor for signaling, the lack of a cytoplasmic transducing kinase domain prevents genome-wide predictions of RLP subfamilies based on sequence comparisons. Therefore, a complete inventory of the RLP family in the genome of different plant species is lacking, and, hence, functional studies have been limited.

The limitation of software based on multiple sequence alignments for identifying RLPs may be overcome with the application of artificial intelligence algorithms developed based on filters that support the point features of these receptors. In artificial intelligence, machine learning (ML) has emerged as a potential tool in molecular biology to analyze massive datasets and extract knowledge from complex biosystems [54]. ML has been extensively used in all sorts of thematic issues, from medicine to robotics [55,56,57]. In plant science, ML has been applied for viral gene identification [58], the diagnosis of bacterial infection [59], salt stress tolerance [60], and the taxonomy of grapevine [61], in addition to global analysis of gene expression, in response to hormones and environmental stresses [62], plant immunity, and miRNA network prediction [54]. Trained models have also been successfully used for functional protein classification in plant genomes [63].

To provide a framework for identifying and predicting RLP function, we developed the RLPredictiOme as a machine learning method associated with Bayesian inference approaches. In addition to six different features to train ML models, the method used multiple datasets based on RLK ectodomains and the hypothesis that RLP lacks the kinase domain but retains the same RLK receptor configuration. It is reasonable to suppose that the RLP family may contain all RLK-identified ectodomains as they may have emerged during evolution from kinase domain-losing RLKs. So far, five RLK different ectodomains-containing RLP groups have been identified [53]. Our ML models could distinguish RLPs from non-RLPs (NRLPs), RLPs from RLKs and classify subfamilies with relatively high accuracy, precision, sensitivity, and specificity. To prove the capacity to predict RLP families, we validated the method with biological experiments describing a new RLP family, designated GDPDL-RLP. The RLPredictiOme may facilitate the prediction and provide new insights into the role of RLPs in plants.

## 2. Results

### 2.1. Revisiting the Ectodomain of the RLK Superfamily in Plants

We performed a survey in the genome of 80 plant species to identify the functional ectodomains of RLKs based on in silico models as a first step for defining the datasets. A total of 40,418 sequences were retrieved. We identified 100 classes of RLK ectodomains associated with C-terminal kinase domains (Table 1). However, most of these ectodomains generated subfamilies with less than 10 members. Sequence identities higher than 0.85 were removed through CD-hit software. Additionally, only sequences with a single membrane segment were selected. A total of 14,787 amino acid sequences were recovered, and their ectodomains were used as positive datasets for filtering RLPs versus NRLPs and RLPs versus RLKs.

Three datasets were created to represent a higher number of negative examples. The first dataset contained 14,973 positive examples and 15,993 negative ones. The second and third ones contained the same examples, 14,973 positives, and 15,973 negative examples. To distinguish RLPs from NRLPs, we used six types of features (see Methods sections) from the three datasets, thus implying a total of 18 training sets. On the other hand, to distinguish RLPs from RLKs, only one dataset with 14,973 positives (ectodomain of the RLKs) and negative (full-length sequence of the RLKs) examples were used, implied in six training sets based on the assumed number of features.

The RLP subfamily members were assigned according to the ectodomains of RLKs. For each training set, 15 classes were considered, and a 16th class, designated Other RLPs, was defined by grouping the smaller subfamilies (Table 2). In some plant species, uncharacterized RLK subfamilies have at least one to ten members and were grouped in the class Other-RLPs. LRR-RLKs, unknown-RLK, S-domain-RLK, and WAK-RLKs are over-represented RLK subfamilies in plants. In contrast, thaumatin, GDPD, and malectin are small subfamilies not represented in all plant species [9]. For each super-represented subfamily, 500 sequences were randomly selected to compose ten additional datasets; thereby, considering the previously mentioned six types of features, 60 training sets were obtained for training.

### 2.2. Feature Analysis

**Figure 1:**
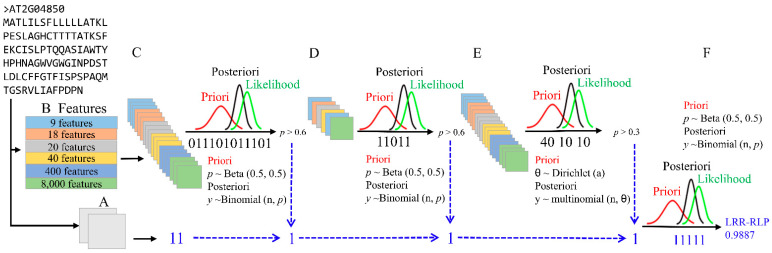
Schematic representation of the RLPredictiOme method. Amino acid sequences are submitted to the method with the sequential filters A to F. (**A**) The signal peptide and segment transmembrane prediction. (**B**) Attribute vector provided to the ML models. (**C**) The first step of the classification to distinguish RLP from NRLP (RLP/NRLP). The result (binary vector) of the classification is submitted to perform Bayesian inference using probability distribution Binomial conjugated with Beta distribution. (**D**) The second classification step to distinguish RLP from RLK (RLP/RLK). The result (binary vector) is submitted to perform Bayesian inference using probability distribution Binomial conjugated with Beta distribution. (**E**) The ML models for subfamily classification is the third step to classify RLP families. The result (numerical vector) of the classification is submitted to perform Bayesian inference through the Multinomial and Dirichlet probability distributions. (**F**) The Bayesian inference for making decisions and final prediction using binary vector resulting from the preview inferences.

We implemented the RLPredictiOme method using six distinct types of attributes (Figure 1). These included (i) the frequency of the chemical properties of amino acid side chains (CPAASC), which have 9 features, and (ii) CPAASC2 extracted from N-terminal and C-terminal regions with 18 features; (iii) the amino acid composition with 20 features and (iv) amino acid composition extracted from N-terminal and C-terminal regions with 40 features (Figure 1B). Furthermore, we used (v) dipeptide and (vi) tripeptide compositions resulting in 400 and 8000 features, respectively. The simultaneous use of six types of features and multiple datasets provided RLPredictiOme with information to apply Bayesian inference (see Section 4) as a powerful ensemble method to make robust predictions.

For the classification models for RLPs/NRLPs (first step, Figure 1C), the tripeptide composition was the feature with the best performance among all tested features of the models built with the RLPs/NRLPs datasets using the logistic regression algorithm (Table 3). The three models built with tripeptide composition achieved accuracy (ACC) of 0.953, 0.955, and 0.953, respectively, and Matthew’s correlation coefficient (MCC) of 0.906, 0.910, and 0.96, respectively. Furthermore, the false discovery rate (FDR) was lower than 0.05.

For the classification models for RLPs/RLKs (second step, Figure 1D), the amino acid composition of the N-terminus and C-terminus and tripeptide composition were the features archiving both the best performance, resulting in ACC of 0.97, MCC of 0.95 and FDR lower than 0.05 (Table 4). In the RLP subfamily models built with RLP subfamily datasets (third step, Figure 1E), the tripeptide composition outperformed the others, with ACC and MCC of 0.984 and 0.866, respectively (Table 5).

### 2.3. ML Model Capacity of Distinguishing RLPs from NRLPs

The ability of the ML models to distinguish RLPs from NRLPs was examined through the predictive capacity of the models created with the RLPs/NRLPs datasets (Figure 1C). The models that classify RLPs/NRLPs were evaluated using 10-fold cross-validation based on the following metrics: ACC, sensitivity, precision, F-measure, specificity, FDR, and MCC. For each dataset, 21 models (21 algorithms) were selected, and the performance results are presented in Table 3. In general, the selected models provided average values for ACC, F-measure, FDR, MCC, precision, sensitivity, and specificity equal to 0.93, 0.934, 0.070, 0.861, 0.948, 0.948, and 0.911, respectively.

### 2.4. ML Model Abilities to Distinguish RLPs from RLKs

To distinguish RLPs from RLKs, we assessed the generality of models constructed with RLP/RLK datasets (Figure 1D). The outcome of 10-fold cross-validations and evaluated metrics for RLPs/RLKs models are shown in Table 4. The quadratic discriminant analysis and gradient boosting classifier with the amino acid composition of the N-terminus, C-terminus, and tripeptide features outperformed the others (Table 4). The average performance of the six models provided ACC 0.968, F-measure 0.967, FDR 0.04, MCC 0.936, precision 0.981, sensitivity 0.981, and specificity 0.955, respectively.

### 2.5. The Ability of ML Models to Classify RLP Subfamilies

To classify the RLP subfamily, we evaluated models built with RLP subfamily datasets using 10-fold cross-validation. The performance of the models was examined by the previously mentioned metrics (Figure 1E). The tripeptide and dipeptide composition features achieved average MCC values higher than 0.90 when using the K-nearest neighbor algorithm. The N-terminus and C-terminus amino acid composition feature achieved an average MCC value of 0.899 using a calibrated classifier and linear discriminant analysis (Table 4). The average performance of the six models provided ACC 0.98, F-measure 0.874, FDR, MCC, precision 0.877, sensitivity 0.87, while MCC varied from 0.759 to 0.953 (Table 5).

### 2.6. Validation of RLPredictiOme

For RLPredictiOme validation, we tested the ML models in combination with Bayesian inference as an ensemble method approach (Figure 1). In the first validation, we submitted 47 near-characterized sequences of RLPs against the RLPredictiOme. The validation data set comprises thirty-nine LRR-RLPs, six LysM-RLPs, two WAK-RLPs, and one salt stress-responsive/antifungal-RLP (Table 6). However, six of these RLPs were not characterized as RLP as they did not have a TM. The test resulted in thirty-seven LRR-RLPs correctly classified, two LysM-RLPs were correctly classified, and two LysM-RLPs were classified as undefined due to relative low probability (*p*) provided by Bayesian inference of the RLP subfamily. The remaining two LysM-RLPs (Q67UE8.1 LYP4 and Q69T51.1 LYP6), one WAK-RLP (AKP45167), and one salt stress-responsive/antifungal- RLP (LOC_Os04g56430.1) were not classified as RLPs due to the TM absence (Table 6).

In the second validation, we used the data of a genome-wide study of RLPs restricted to the LRR-RLP subfamily [53]. The 57 LRR-RLPs of Arabidopsis were submitted to the RLPredictiOme predictor. As a result, 47 LRR-RLPs were classified correctly, although 13 LRR-RLPs did not have a signal peptide (SP). One LRR-RLP harboring SP was undefined, and the remaining nine LRR-RLPs were not classified as RLP due to the TM absence (Table 7). Interestingly, the AtRLP4 protein was previously classified as LRR-RLP; however, the RLPredictiOme classified it as malectin-RLP due to one di-glucose binding domain within the endoplasmic reticulum-associated LRR domain.

In a third validation, we selected 148 LRR-RLPs described in a genome-wide study of rice RLPs [64] (Table S1). The results show that 78 LRR-RLPs with SP and TM were correctly classified with a relatively high probability (greater than 0.98). Additionally, from 73 LRR-RLPs with a single TM, 71 were correctly classified, whereas 2 were classified as Other-RLPs with an estimated probability ranging from 0.792 to 0.805. Only four predicted LRR-RLPs from rice were classified as NRLPs; two lack both SP and TM, and two do not harbor TM. The fourth validation was carried out to ensure that RLPredictiOme does not randomly classify proteins. For this, 100 randomly generated sequences were confronted against RLPredictiOme, and all sequences were classified as NRLP in the first step (Table 8).

### 2.7. High Throughput Prediction of RLPs in the Arabidopsis Genome Using RLPredictiOme

We performed high throughput prediction by submitting the Arabidopsis sequences against RLPredictiOme. The cutoff tuning for the probability filter was assumed to be 0.6 in the first two-step and 0.7 in the last step (Figure 1F). In the third step, the probability estimates were more flexible in order to predict the RLP subfamilies.

From this genome-wide prediction, RLPredictiOme classified 176 RLP sequences into 15 subfamilies (Table S2). Table 9 summarizes the correct predictions within the subfamily. The number of proteins with unknown functions is highlighted in red, whereas the blue description represents the RLPs subfamilies predicted in other subfamilies. The LRR-RLPs subfamily contained 49 members. Three new members (AT5G37360, AT5G19230, and AT4G28560), predicted with relatively high probability, were not classified into a known subfamily, whereas two sequences were incorrectly classified. Interestingly, AtRLP4 has two domains, an LRR domain, and an endoplasmic reticulum protein-associated Di-glucose binding domain, which characterizes malectin proteins. The RLPredictiOme method classified the AtRLP4 into the malectin-RLP subfamily (see Table S2).

The candidate sequences with a legume lectin domain were classified into two RLP subfamilies, B-Lectin-RLP and L-Lectin-RLP (Table S2). Only one member was classified as B-Lectin-RLP with an unknown function, while six members were classified into the L-Lectin-RLP subfamily, also designated as unknown function proteins. Seven proteins were classified incorrectly into this subfamily. The 20 Lysin motif-containing candidate proteins were classified as LysM-RLP (Table S2). Two (AT1G77630.1 and AT2G17120.1) of the three previously characterized LysM-RLPs [65] and two classified LysM-RLPs (AT3G06360.1 and AT5G26270.1) belong to subfamilies previously identified as unknown function subfamilies, and one sequence (AT1G63550.1) belongs to the salt stress response/antifungal-RLP family. The other 15 sequences may belong to the lipid transfer protein family, not yet characterized. Additionally, the ectodomain lipid transfer family associated with a kinase domain was allocated in the other-RLP group as probable lipid transfer-RLK. Twelve sequences were classified as probable lipid transfer-RLP; however, this misclassification occurred in the LysM-RLP and unknown-RLP groups, which may be functionally similar. It may be due to the over-representability of these two mentioned groups.

In the malectin-RLP subfamily, RLPredictiOme correctly classified two members previously characterized (AT1G28340.1 and AT1G24485.1). Four candidate members were identified into subfamilies of unknown function, and seven sequences were incorrectly predicted (Table S2). Furthermore, the third previously identified malectin-RLP (AT3G46240.1) was predicted as an RCC1-RLP. This subfamily has seven predicted members without known functions. One salt stress response/antifungal-RLP was predicted within this family. The salt stress response/antifungal-RLPs had four members correctly classified and four predicted within other subfamilies (three in WAK-RLP and one in RCC1-RLP). The S-domain-RLP had a correctly and an incorrectly predicted sequence (Table S2).

As for the thaumatin-RLP subfamily, all six members were correctly predicted (Table S2). The WAK-RLP subfamily correctly predicted five members but also incorporated one candidate sequence with an unknown function and three salt stress response/antifungal-RLPs. Ectodomains without a functional domain were classified within a subfamily designated unknown-RLPs. This group also includes RLPs harboring the ectodomains PERK-like, extensin, RKF3-like, CrRLK1, and RLK10-like proline-rich proteins. RLPredictiOme predicted 46 sequences with unknown functions classified as an unknown-RLP subfamily (Table S2). The protein sequences, which are not classified correctly or have a low relative probability of subfamily classification, were designated as undefined and not considered RLPs. In summary, a total of 78 proteins were classified in this group (Table S2).

RLPredictiOme identified probable lipid transfer-RLPs, considered a novel RLP class associated with RLKs, yet to be characterized. Furthermore, three new classes of RLPs were predicted: plastocyanin-like-RLP, ring finger-RLP, and glycosyl-hydrolase-RLP, which contained eight, five, and seven members, respectively. Interestingly, five glycerophosphoryl diester phosphodiesterase family (GDPDL members were predicted as other-RLPs. As a rare protein family in plants, we selected GDPDL-RLP to carry out an experimental validation for these receptor-like protein candidates. The number of predicted RLPs in each subfamily is shown in Table 9.

### 2.8. GDPDL Family Downstream Analysis

**Figure 2:**
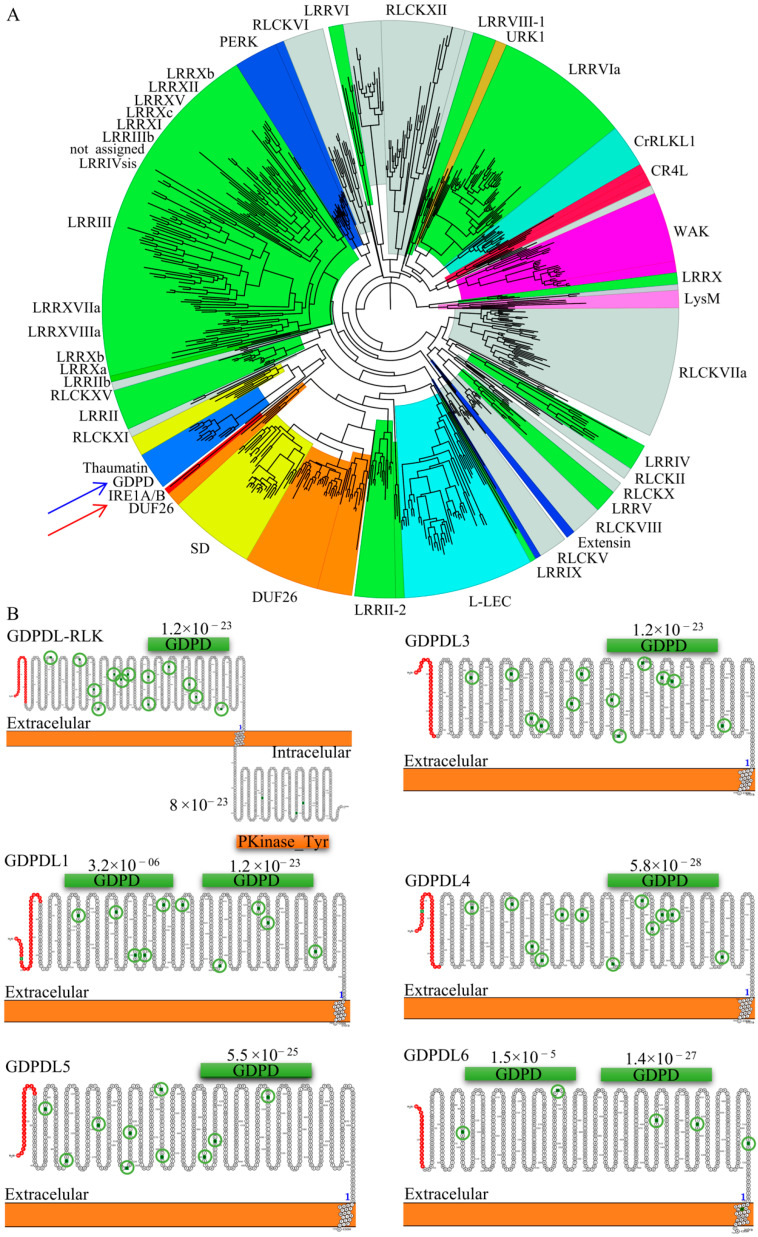
Analysis in silico of the GDPDL-RLPs. (**A**) Phylogenetic tree of the kinase catalytic domain of RLKs, IRE1A and IRE1B. (**B**) The topology of GDPDL-RLPs.

Phylogenetic analysis of the kinase domain of the RLK family and the kinase domain of IRE1A and IRE1B, endoplasmic reticulum (ER)-specific protein kinase, clustered the kinase domain of GDPDL-RLK and thaumatin in the same group distinct from the ER kinases (Figure 2A). These results suggest that GDPDL-RLKs are not ER transmembrane proteins. The secondary structure and the topology of GDPDL show that the N-terminal region of GDPDL-RLK is composed of a signal peptide, a GDPD domain, and more than 10 candidate sites for N-glycosylation (Figure 2B). As an RLK, GDPDL-RLK contains an ectodomain facing the extracellular space, a transmembrane segment, and a cytoplasmic portion harboring the kinase domain. The topology of classified GDPDL-RLPs fits a typical RLP configuration with an N-terminal peptide signal, the glycerophosphoryl diester phosphodiesterase ectodomain, the transmembrane segment, and it lacks a short C-terminal cytoplasmic domain. GDPDL1 and GDPDl6 harbor two glycerophosphoryl diester phosphodiesterase domains, whereas GDPDL3/4/5 has a single domain localized in a similar position compared with GDPDL-RLK.

The molecular evolution of the new GDPDLs and the GDPDL-RLK ectodomain was investigated by calculating the ratio between non-synonymous and synonymous substitutions (Ka/Ks). Compared to the full-length sequence of GDPDL-RLK, only the gene pair GDPDL-RLK/GDPDL6 with a ratio of Ka/Ks > 1 may have undergone a positive selection (Table 10). The ectodomain sequence of GDPDL-RLK compared with gene pairs GDPL1/3/4 was submitted to purifying selection, as suggested by their Ka/Ks ratio < 1 and *p*-value < 0.05. The divergence time of GDPL1/3/4 was 23.7, 32.5, and 120.1 Mya. These results suggest that despite the divergence time of GDPL1/3/4 compared to the GDPDL-RLK ectodomain, the higher frequency of synonymous mutations may have maintained the GDPL1/3/4 and the ectodomain GDPDL-RLK functionally similar.

### 2.9. Identification of GDPDLs- and SNC4-Interacting Proteins from Arabidopsis

**Figure 3:**
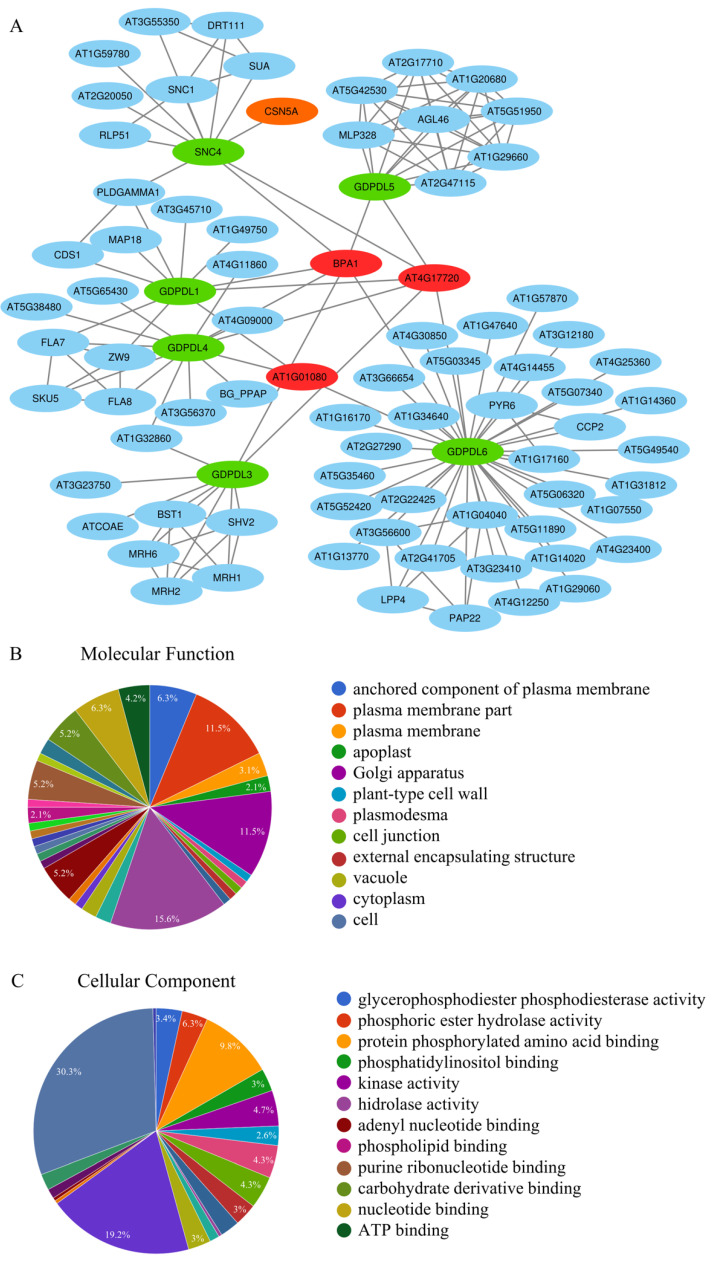
GDPDL-RLPs-interacting Arabidopsis proteins. (**A**) GDPDL-RLP-interacting proteins were identified in the Arabidopsis interactome, and the network was assembled by the Cytoscape software. GDPDL-RLPs and SNC4 (GDPDL2) are indicated in green, GDPDL-specifically interacting proteins in light blue, RNA-binding proteins, which interact with all 6 GDPDLs, including GDPDL_RLK (SNC4), are shown in red. In orange, CSN5A as a central hub of plant-pathogen interactions (**B**) Gene enrichment of proteins under the molecular function term from the GDPD-RLP-Arabidopsis protein-protein interactions (PPI) network. (**C**) Gene enrichment of proteins from the GDPD-RLP-Arabidopsis PPI network under the cellular component term.

Protein–protein interactions between the GDPDLs and GDPDL-RLK, also designated SUPPRESSOR OF NPR1, CONSTITUTIVE 4 (SNC4), and the Arabidopsis proteins were identified in silico through the protein–protein interactome using Cytoscape software and several databases (BioGRID database, Arabidopsis interactome database, and the String database). This procedure identified the protein-protein interaction (PPI) network containing GDPDLs and directly interacting Arabidopsis proteins (Figure 3). The GDPDL6 formed the largest hub (degree 38). Among the GDLDL6-interacting proteins, the glycogen synthase kinase 3/SHAGGY-like kinases (GSKs-AT1G57870) may represent a candidate protein for signaling (Figure 3A, Table 11). Although GSKs have been recently discovered in plants, evidence suggests that they are involved in different biological processes, such as brassinosteroid signaling, flower development, and injury responses [66]). The node-hub GDPDL5 contains the AtMLP328 pathogenesis-related protein and other proteins of unknown function (Figure 3A, Table 11). The AtMLP328 is a member of the major latex protein-like (MLPL) gene family responsible for promoting vegetative growth and delaying flowering.

The cluster of GDPDL3-interacting proteins includes the BRASSINOSTEROIDE INSENTIVE 1 (BRI1)-ASSOCIATED RECEPTOR KINASE 1 (BAK1), also designated SOMATIC EMBRYOGENESIS RECEPTOR KINASE 3 (SERK3). BAK1 has been shown to function as a co-receptor for many RLKs, including the recruitment of receptor-like proteins and SOBIR to form a heterodimeric complex upon recognition of ligands by RLPs, for example, RLP23-SOBIR1-BAK1, cf-4-BAK1/SERK3- SOBIR1, RE02-BAK1-SOBIR1, and RXEG1-BAK1-SOBIR1 [46,49,51,67] (Figure 3A, Table 11).

The interactions of GDPDLs- and SNC4 converge to centralized hubs represented by BPA1, AT1G01080, and AT4G17720 (BPL1), which contain an RNA binding motif (Figure 3A, Table 11). The BPA1 protein has been shown to interact with Arabidopsis ACD11, which induces the expression of genes associated with disease resistance and genes involved in the ROS-mediated response defense upon recognizing fungal elicitors [68,69]. Furthermore, BPA1 and BPL1 are induced during geminivirus infection [70]. The GDPDLs-Arabidopsis PPI network is enriched for proteins involved in plant defense response to pathogens and vegetative growth, indicating that this new RLP family may be involved in immunity and developmental signaling.

To gain further insights into the cellular processes involved by GDPDLs, we performed functional enrichment analyses of their direct interactors. In all three categories, biological process, molecular function, and cellular component ontology, we identified enriched GO terms with a *p*-value < 0.05. Under molecular function, we identified enriched terms for Glycerophosphodiester phosphodiesterase activity, nucleotide binding, purine ribonucleotide binding, and hydrolase activity, which are unusual enzyme activities associated with membrane receptor activity (Table 10). Under the cellular component ontology, we observed an over-representation of proteins from plasma membrane term, membrane-bounded term, and plant-type cell wall term, which may suggest that the location and functional activities of these hubs are specific to transmembrane proteins. (Figure 3B). Under the biological process ontology, the response to defense response, response to external stimulus, and developmental growth term represented significantly enriched GO terms, which show that this family of proteins may be related to immunity and plant development (Table S3).

### 2.10. The Expression Profile of the GDPDLs in Response to Pathogens and Different Organs

**Figure 4:**
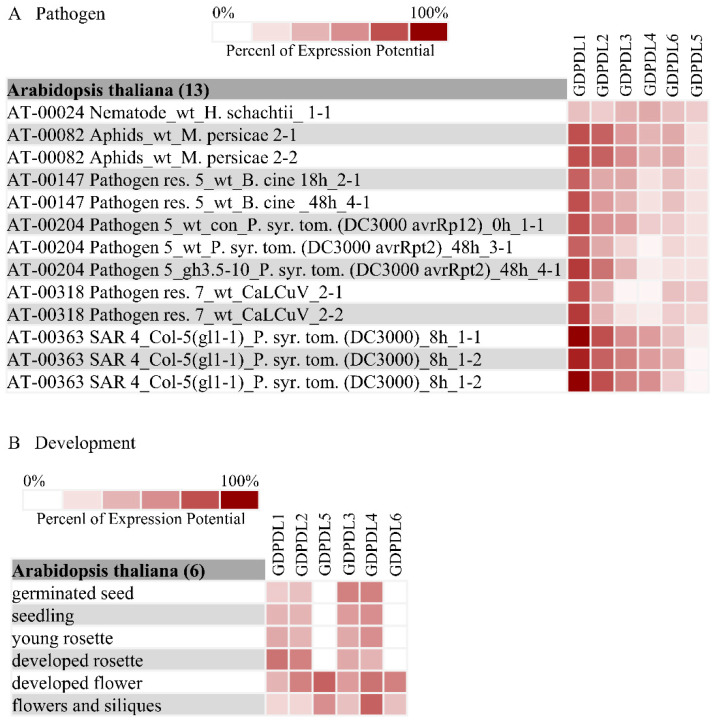
Analysis in silico of the expression of GDPDL-RLPs. (**A**) The expression profile of the GDPDL-RLPs in response to pathogens. (**B**) The expression profile of the GDPDL-RLPs in different organs and developmental stages.

To gain insights into the potential defense response of the GDPDLs genes and to validate these candidate receptor-like proteins as expressed genes, we investigated their expression profiles through publicly available expression datasets using the gene investigator (NEBION, AG, Zurich, Switzerland; www.genevestigator.com, academic free license, accessed on 28 February 2020) (Figure 4A). From these microarray data, GDPDL1-RLK was induced by aphids, the bacteria Pseudomonas syringae, and the begomovirus cabbage leaf curl virus (CabLCV), but not by nematodes. Likewise, GDPDL2-RLP is induced by bacteria and aphids, and begomoviruses to a lesser extent. *GDPDL3-RLP* and *GDPDL4-RLP* are upregulated by aphids and bacteria and down-regulated by begomovirus. *GDPDL5* and *GDPDL6* are not induced by aphids and bacteria but downregulated by CabLCV. As for organ-specific expression, except for *GDPDL5-RLP* and *GDPDL6*-*RLP* which only expressed in flowers and siliques, the remaining GDPDLs are expressed in all organs tested, although to a different extent (Figure 4B). While *GDPDL1* and *GDPDL2* expressions predominate in the developed rosette, *GDPDL3* is highly expressed in germinated seeds, and the *GDPDL4* expression is fairly distributed in all organs.

**Figure 5:**
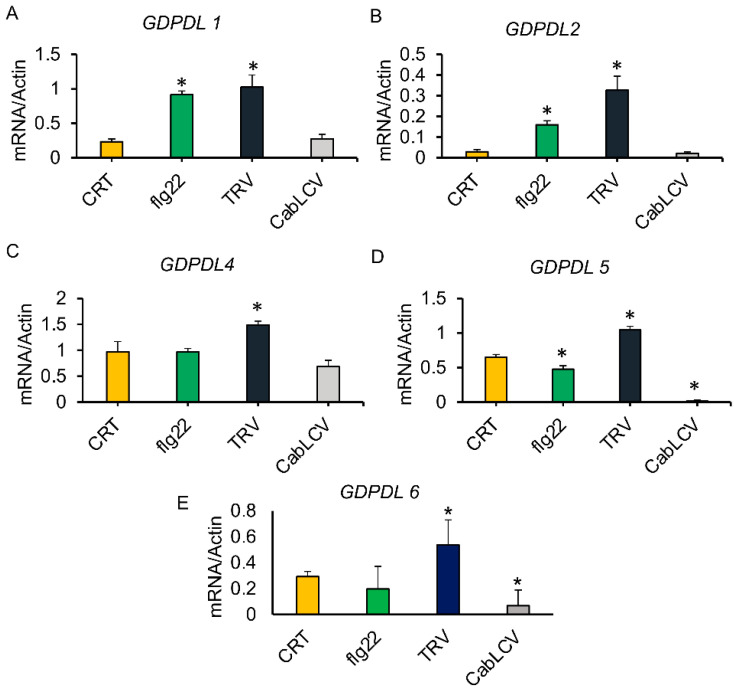
Expression analysis of the GDPDL genes in response to biotic signals. For the flg22-induced expression of GDPDLs (as indicated in the figure), 12-day-old Arabidopsis seedlings were treated with 100 nM flg22, and total RNA was prepared from 100 µg of a pool of 10 flg22-treated plants. For TRV infection, Arabidopsis leaves were mechanically inoculated with TRV from *N. benthamiana*-infected leaves, and TRV infection was diagnosed by PCR. For CabLCV infection, Arabidopsis plants were inoculated with infectious DNA-A and DNA-B clones, and viral accumulation was monitored by PCR. After 15 days of TRV inoculation and 21 days of CabLCV inoculation, total RNA was extracted from a pool of 10 TRV- and CabLCV-infected plants. The transcript accumulation of the indicated genes was monitored by quantitative RT-PCR with gene-specific primers. The gene expression was calculated by the 2^−∆CT^ method using actin as an endogenous control. The error or standard bars indicate the mean ± SD (*n* = 3, technical replicates). * *p* < 0.05.

**Figure 6:**
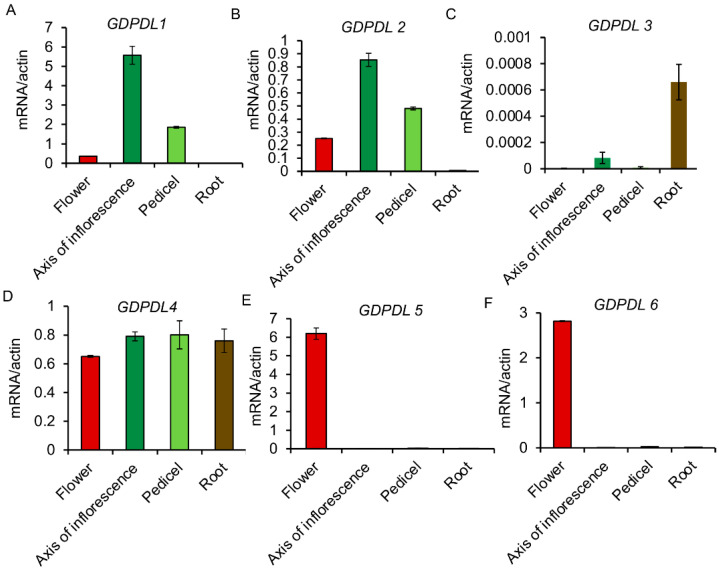
Organ-specific expression of the GDPDL genes. Total RNA was extracted from different Arabidopsis organs (as indicated in the figure) of 35-day-grown plants. We used 3 samples of different pools of 10 plants each (therefore n = 3, biological replicates), and the transcript levels of the indicated genes (GDPDL1, GDPDL2, GDPDL3, GDPDL, GDPDL5, and GDPDL6) were determined by qRT-PCR using gene-specific primers. The gene expression was calculated by the 2−∆CT method using actin as an endogenous control. The error or standard bars indicate the mean *±* SD (n = 3 biological replicates + *n* = 3 technical replicates each) of three independent experiments.

Pathogen-induced and organ-specific expression profiles of the predicted GDPDL-RLP genes were confirmed by qRT-PCR (Figure 5 and Figure 6). We also monitored the expression of the *GDPDL-RLP* genes in response to infections with tobacco rattle virus (TRV) and CabLCV. The antibacterial immune responses (PTI) were activated by treatment with flg22, and the expression of GDPDLs was monitored (Figure 5). Consistent with the microarray data, *GDPL5* and *GDPL6* expression was not affected by flg22 treatment but was downregulated by CabLCV, whereas *GDPDL1* and *GDPDL2* were induced by flg22 and CabLCV. All 5 GDPDLs analyzed by qRT-PCR were induced by TRV, a plant RNA virus. Remarkably, these GDPDL proteins are interconnected via interactions with RNA recognition motif-containing proteins, which form centralized hubs in the network interaction (Figure 3A, Table 11). This result may suggest an involvement of GDPDLs in the antiviral response induced by an RNA virus.

We also confirmed the expression profile of these GDPDL genes in different tissues by qRT-PCR. We used the root, pedicel, inflorescence axis, and flower tissues. The expression levels of *GDPDL1* and *GDPDL2* are similar in all tissues (Figure 6A,B). The highest expression levels were identified in the inflorescence axis and pedicel, suggesting distinct functions in development. Likewise, *GDPDL3* is most expressed in roots and barely detected in other tissues (Figure 6C). Interestingly, the expression levels of *GDPDL4* are regular in all tissues, showing that this protein may have a varied role during development (Figure 6D). In contrast, qRT-PCR confirmed that the *GDPDL5* and *GDPDL6* transcripts accumulated to elevated levels in flowers (Figure 6E, 6F). These gene expression analyses confirmed that GDPDL-RLPs are expressed in response to stimuli and development, substantiating the argument that they may form a new class of RLPs involved in immunity and developmental signaling.

## 3. Discussion

Due to the functional relevance of the RLK family in several biological processes, this large family has been extensively studied in different plant species [6,9,71,72,73,74,75]. In contrast, far less is known about the plant RLP family, despite their conceptual relevance in signaling modules. RLPs can perceive external signals but depend on association with RLKs for signal transduction due to the lack of a cytoplasmic kinase domain at the C-terminus. The absence of a conserved kinase domain precludes using sequence comparison algorithms for genome-wide studies of the plant RLP family. Thus, identifying RLPs in plant genomes is challenging, and few RLPs have been described in plant species. Moreover, a large-scale RLP prediction tool has not been developed. Here, we developed the RLPredictiOme method based on machine learning approaches and Bayesian inference for the throughout prediction of RLPs.

Typically, the ML classification models applied in plant molecular biology require actual data to train ML-supervised algorithms [54,76,77,78]. The RLPredictiOme can predict RLP subfamilies using the RLK ectodomain and simultaneously six types of features during the prediction process. The prediction model consists of three steps subsequently built with trained models and different algorithms capable of distinguishing RLP from NRLP, RLP from RLKs, and finally, predicting an RLP subfamily. The combination of several ML models with different algorithms has been applied for protein and viral sequence classification [58,63]. Using different classifiers requires methods that compile the results of the classifiers into a single final prediction. Some methods have used different techniques for model combinations, including a majoritarian vote of the classifiers or an average probability for the classifications [63,79]. The approaches applied in the RLPredictiOme by combining models are based on the success and failure of predictions, which are modeled with Bayesian inference. In each step after the classifications, the Bayesian inference is applied. The validation results of the RLPredictiOme showed high probabilities for classifying RLPs proteins (See Table 7, columns RLP-NRLP Probability, RLP-RLK Probability, and RLP-Subfamily Probability). In contrast, NRLP proteins were predicted with a lower probability (Table 8). Finally, based on the probability of Bayesian inferences for each step, the last step is used as a decision-making process for the prediction of RLPs (Figure 1F). The RLPredictiOme predicts RLP proteins with a probability ranging from 0.79 to 0.99 (See Table 7, Table 8 and Table 9, column Decision probability). Thus, the ML models can be successfully combined with Bayesian inference to perform robust high-throughput predictions of RLPs in plant genomes.

The RLPredictiOme could predict new RLP subfamilies with higher probability in all steps, although groups less represented were also classified into a corresponding subfamily, yet with lower probability. Furthermore, groups less represented by RLPs tended to be classified within other RLP subfamilies. This other RLP classification was the case of the probable lipid transfer-RLP subfamily, which shares similar functional characteristics with LysM-RLP. The lipid transfer proteins (LTPs), already described as non-specific lipid transfer proteins (nsLTPs), contain an eight-cysteine motif that is stabilized by four disulfide bonds (Wang et al., 2019). The probable lipid transfer family (PLT)-RLPs found by RLPredictiOme harbor a five-cysteine motif (CC-Xn-CXC-Xn-C) in the TP_2 functional domain differently from the typical nsLTPs [80]. Phylogenetics relationships, structure, and genome-wide distribution of LTPs, involved in response to nematodes, have been described in cucumbers (Wang et al., 2019). Furthermore, PLTs have been shown to play a crucial role in regulating various plant biological processes and responding to biotic and abiotic stress [81,82]. Due to evidence of association with kinases, PTL-RLPs may be classified as a new subfamily of RLPs or may represent an expansion of the LysM-RLP subfamily, which exhibits similar functional roles.

*In silico* and in vitro analyses of GDPDL-RLPs confirmed the efficiency of the RLPredictiOme in identifying a new family of RLPs based on the ectodomain of GDPDL-RLK sequences. The GDPDL-RLK is a reduced class of RLKs in plants. Among all the plant species analyzed, they have been found only in *Arabidopsis halleri* (Araha.28943s0001.1), *Arabidopsis lyrata* (475793), *Arabidopsis thaliana* (AT1G66980.1), *Boechera stricta* (Bostr.26959s0213.1, Bostr.26959s0216.1), and *Brassica rapa* (Brara.K00110.1), all from the *Brassicaceae* family, and *Capsella grandiflora* (Cagra.0792s0001.1) and *Panicum virgatum* (Pavir.6NG294600.1), from the *Poaceae* family. Despite only one GDPDL-RLK in the Arabidopsis genome [83], RLPreditiOme identified five sequences as GDPDL-RLP. Furthermore, the GDPDL-RLK subfamily has been maintained in only a few plant species; thereby, this family is likely suffering a reduction in size and distribution. The GDPDL2-RLK (AT1G66980) has been previously characterized as SNC4, an atypical receptor-like kinase with a predicted extracellular GDPD domain involved in regulating plant immunity [84]. The glycerophosphodiester phosphodiesterase (GDPD) hydrolyzes glycerophosphodiesters into sn-glycerol-3-phosphate (G-3-P) and plays a significant role in various biological processes [84]. The GDPDL2-RLK ectodomain is structurally similar to the predicted GDPDL-RLPs (Figure 2B). Molecular evolution investigated by calculating ka/ks of GDPDL-RLP-GDPDL-RLK pairs revealed a significant rate of synonymous substitutions indicating that although the kinase domain has been lost, the functional characteristics of the ectodomain remained conserved among evolution (Table 10).

A common feature of the RLK subfamilies is that they are often more extensive than the RLP subfamily counterparts, which suggests that some members of the RLK subfamilies have lost their conserved C-terminal kinase domain during evolution. In contrast, RLPredictiOme identified a new RLP subfamily, GDPDL-RLP, which seems to have expanded compared to the corresponding GDPDL-RLK subfamily. Therefore, we were interested in examining the expression profile of the GDPDL-RLP members to ensure a basal level of expression during development or in response to pathogens. In silico analyses from publicly available expression databases indicated that the RLP members display differential expression profiles in response to pathogens and different organs, indicating that they may be involved in development and immunity.

*GDPDL1* (*GDPGL-RLP*) has been previously shown to be expressed in the rosettes of Arabidopsis plants [85]. We confirmed by qRT-PCR that *GDPDL1* is expressed in the pedicels of the rosette and flowers. GDPDL1 has also been shown to be involved in processes that confer rigidity to the cell wall, related to defense against insects, nematodes, and oomycetes [85]. Accordingly, the previously published microarray data showed a high *GDPDL1* induction in response to these pathogens and pests.

*GDPDL1* and *GDPDL2* displayed the highest expression in pedicels and flower stems and were highly expressed in response to pathogens and flg22. Among all members of this new GDPDL family, *GDPDL3* was barely detected in the organs examined except in roots, consistent with its role in root morphogenesis [86]. *GDPL4* was uniformly expressed in all organs evaluated. *GDPDL4* has been described as a highly expressed gene in rosettes and is involved in the development of root hair [85,87]. Therefore, the expression profile of already described GDPDLs is coordinated with their assigned function.

Two undescribed family members, *GDPDL6* and *GDPDL5*, displayed elevated levels of expression in flowers, showing that both genes may be involved in the development of reproductive organs and structures. These genes are also induced by biotic signals, as RT-qPCR demonstrated they were upregulated by TRV infection and microarray data showed their slight induction by nematodes. We found that all GDPDLs are induced by the RNA virus TRV and form interconnected protein-protein hubs with RNA binding proteins. It would be relevant to investigate whether GDPDLs function in RNA virus infection. The expression pattern and evolution studies of members of the GDPGL-RLP subfamily further substantiate the notion that the members of this subfamily have maintained functional domains and may play relevant roles in development and plant defense.

## 4. Materials and Methods

### 4.1. Reclassification of the Plant RLK Ectodomains for Composing Datasets

The amino acid sequences of 80 plant species were retrieved from the Phytozome database (version 11.1 by DOE Joint Genome Institute, Lawrence Berkeley National Laboratory; https://phytozome.jgi.doe.gov/, accessed on 28 February 2020). We applied filters to remove unknown sequence proteins without functional annotation. The sequences were re-annotated using SMART (version 8.0, licensed by Creative Commons Licence, manufactured by Heidelberg, Germany; smart.embl-heidelberg.de) and Pfam (pfam.sanger.ac.uk) databases. Then, the amino acid sequences containing a predicted kinase domain were selected. The signal peptide was predicted using SignalP v.4.0 [50] and Phobius [88] software, whereas the transmembrane segment was identified using TMHMM [89] and Phobius software. Then, the sequences were filtered by using the criteria based on the presence of a signal peptide and a transmembrane segment. Furthermore, the redundant sequences were removed through CD-HIT algorithm [90]. Subsequently, the amino acid sequences were grouped according to the functional domain of the extracellular ectodomain (LRR-RLK, WAK-RLK, and LysMRLK, for example) [9,91].

### 4.2. Dataset Composition

For the classification of RLPs, we used three steps: two steps of binary classification and one multilabel classification. In summary, the first stage compares RLPs with other families of NRLP; the second compares RLP with receptor-like kinases (RLKs); and the third performs the classification of a protein sequence within an RLP subfamily using the functional ectodomain present in RLKs. In the first stage, the training dataset consisted of amino acid sequences containing the extracellular ectodomain, the region of the membrane segment, and the cytoplasmic region that precedes (upstream) the kinase domain of RLKs (but without the kinase domain) as a positive class (RLP). The negative class was composed of full-length amino acid randomly selected sequences (NRLP); the sequences of the positive class were removed from the negative dataset. The dataset was divided into three different datasets to increase the number of negative examples.

In the second stage, the positive class contained the training dataset (RLP), and the negative class used the full-length amino acid sequences of RLKs. In the third stage, the data from RLP positive classes were labeled according to the reclassification of RLKs based on their ectodomain. In this case, a putative LRR-RLP, for instance, contained an ectodomain of the leucine-rich repeat kinase receptor-like kinase (LRR-RLK), a transmembrane segment, and a short cytoplasmic region excluding a kinase domain. Furthermore, the whole dataset was distributed into ten different sub-datasets to work around the computational time limitations of the training.

### 4.3. Feature Extraction

Six types of feature types representing residue frequency composition were calculated for each residue sequence. These included (i) amino acid composition frequency of full-length sequence, (ii) amino acid composition frequency (monopeptide) of the N-terminal and C- terminal regions, (iii) dipeptide frequency, (iv) tripeptide frequency, (v) frequency of chemical properties of amino acid side chains (CPAASC), and (vi) CPAASC2 frequency of the N-terminal and C-terminal regions. A numerical feature vector was created for each sequence of positive and negative datasets. The CPAASC feature describes the frequency of the chemical properties of amino acid side chains, such as positively charged, negatively charged, polar uncharged, aromatic, nonpolar aliphatic, hydrophobicity, volume, and mass of the total number of amino acids in the full-length peptide sequence [63]. In contrast, the CPAASC2 is calculated by the frequency of the chemical properties of amino acid side chains of the N-terminal and C-terminal regions. The full-length sequence is split into two equal (or nearly equal) regions, and the proportion of amino acid composition was also calculated for each of these regions. We consider the N-terminus the first region of the complete amino acid sequence and the C-terminus the second region of the full-length sequence.

The amino acid composition feature describes the frequency of an individual amino acid type within the total number of amino acids in the full-length peptide sequence (Saravanan and Gautham, 2015). The amino acid composition comprises 20 features (ACDEFGHIKLMNPQRSTVWY). The amino acid composition frequency is calculated by the individual amino acid type of the N-terminal and C-terminal regions. The amino acid composition frequency in the N-terminal and C-terminal regions comprises 40 features. The dipeptide frequency describes all combinations of amino acid pairs and comprises 400 features [92]. The tripeptide frequency describes all combinations of three amino acids resulting in 8000 features [93].

The six types of features were used to train all classification models in the three proposed steps. In summary, three training datasets totaling 18 training sets were created for each feature type to compare RLPs with NRLPs proteins (first stage). However, to compare RLPs with RLKs (second stage), one training dataset for each feature type was created. Finally, to classify RLPs within a subfamily (third stage), ten training datasets for each feature type were created, resulting in 60 training sets.

### 4.4. Dealing with Imbalanced Datasets

The superfamily RLK in plants has been broadly characterized and is subdivided into different groups with a different number of members in the subfamilies. The LRR-RLK is the largest subfamily, whereas other subfamilies have a lower frequency of plant members; we used the SMOTE algorithm [94] to oversample the minority class, resulting in a balanced dataset. The SMOTE creates synthetic samples based on the values of the features from the minor class.

### 4.5. Machine Learning Algorithms

The RLPredictiOme method embeds several ML models built with the previously described training sets. This study tested 20 ML algorithms to select the one that suits the supervised learning task. Those algorithms are implemented in the Python library Scikit-learn v.0.22.1 [95]. The algorithms AdaBoost, probability calibration, Gradient Boosting, K-Nearest Neighbors, Linear discriminant analysis, Logistic Regression, and Deep Neural Network were selected, respectively, to compose RLPredictiOme [96,97,98,99,100,101,102,103,104].

### 4.6. Performance Assessment of the Models

The evaluation metrics used in bioinformatics were applied to choose the most efficient algorithms and training models. We evaluated accuracy, F-measure, false discovery rate (FDR), Mathew’s correlation coefficient (MCC), precision, sensitivity, and specificity for each training set and algorithm. These metrics are calculated based on the confusion matrix (contingence matrix) using the number of true positives (TP), true negatives (TN), false positives (FP), and false negatives (FN), respectively. For multi-class models, PyCM python library was used (multi-class confusion matrix library in Python) [105].

### 4.7. Bayesian Inference in Ensemble Methods

Ensemble methods under an ML approach combine the predictions of several classification models with improving the overall performance. Thus, it attempts to avoid misclassification due to noise, bias, and data variance reductions. In an ensemble method, several models are used to predict each data instance. In the binary classification contrasts involving the models RLPs versus NRLPs, and RLPs versus RLKs, we assumed the results provided by *n* independent Bernoulli trials (0 or 1 values) with probability parameter *π*. Thus, the number of successes (*x*) derived from these trials follows a binomial distribution [106]. In this context, we assumed a Beta distribution as the prior distribution for *π* [107]. Under the Bayes theorem, the posterior distribution for *π* (probability of success of classification) is a beta distribution and is conjugated with a binomial distribution. The multilabel models to classify RLP sub-families have different probabilities of success. Thus, the sum of the classification success for each subfamily follows a multivariate generalization of the binomial distribution, named multinomial distribution. We assumed the multinomial distribution for response vector *x* and probability of observed, and N is a vector of the total counts in each RLP sub-families. Thus, the data distribution assumes a multinomial model for all trials. The prior probability widely used for multinomial models is the Dirichlet distribution, which presents the parameters *π* and *θ*. The data vector (*x*) accounts for the total counts in each RLP sub-family.

We perform Bayesian inference using the Bayesian statistical modeling and PyMC3 Python library, which uses the Markov chain Monte Carlo (MCMC) algorithms to explore the posterior distributions [108]. Based on previous analyses with MCMC chains, we opted to use a single chain with 10,000 iterations per amino acid sequence. We used burn-in to 2000 iterations and four chains for all models. The Gibbs sampler algorithm was used to generate random samples from the posterior distribution for all analyses [109].

### 4.8. Classifier Evaluation Strategy

The classification models were evaluated using 10-fold cross-validation. Thus, the data were divided into ten subsets, assuming the training with nine datasets and validation with one dataset. This procedure was repeated ten times, whereas the testing for the RLPredictiOme method was performed with three independent datasets. One dataset was composed of 44 RLPs already described in the literature, and other datasets with 57 LRR-RLPs and legume-like (L-type) lectins, G-type lectins, calcium-dependent (C-type) lectins, and the lectin-like Lysin-motifs (LysM) described in Arabidopsis [53,110,111]. In addition, 100 random amino acid sequences were created by an in-house algorithm to demonstrate that the classifiers do not calculate random predictions.

### 4.9. RLP Subfamilies Downstream Analysis

The function domain prediction analysis was carried out with the Pfam database (version 31, licensed by Creative Commons Zero (“CC0”), manufactured by European Molecular Biology Laboratory, European Bioinformatics Institute (EMBL-EBI; Hinxton, Cambridge; http://pfam.xfam.org/) with a Hidden Markov Model (HMM) algorithm implemented in Hummer software. The signal peptide and transmembrane segment were predicted with SignalP v.4.0 and TMHMM software, respectively [50]. The topology diagram was performed with Protter Web server [112]. The sequence alignment of the RLP superfamily was conducted using the Muscle algorithm (version V1.4.4 by EMBL-EBI, Hinxton, Cambridges, United Kingdom; www.ebi.ac.uk/Tools/msa/muscle/). The phylogenetic analysis was performed by the maximum likelihood statistical method with 10.000 bootstraps using FastTree software [113]. The tree was edited using the FigTree (version V1.4.4 by Andrew Rambaut; http://tree.bio.ed.ac.uk/software/figtree/) software. The gene expression of the glycerophosphoryl diester phosphodiesterase RLP subfamily was investigated through the meta-analysis of transcriptomes using Geneinvestigator V3 [114] and ePlant [115] for the expression in tissues and responses to pathogens.

### 4.10. Protein-Protein Interaction (PPI) Network Analysis

GDPDLs- and SNC4-interacting proteins from Arabidopsis were used as a query term to identify their respective interactions described in the BAR database (Genome Evolution and Function (CAGEF, University of Toronto, Toronto, Canadá; http://bar.utoronto.ca/interactions/). The IntAct and Biogrid databases were selected for searching. The protein–protein interactions (PPI) were visualized in the Cytoscape software (version 3.8.1, licensed by LGP, manufactured by National Resource for Network Biology (NRNB, USA; https://cytoscape.org/), which allowed us to spot the firework topology of the interactions network and measure the network centrality metrics for each protein. We used betweenness, closeness, eccentricity, and degree. Briefly, the betweenness centrality in the PPI network of the graph G = (V, E) was calculated by the number of times a protein interacts along the shorter paths among all nodes. The closeness centrality of a protein *v* is the sum of the shortest path distances from *w* to all other proteins. The eccentricity centrality of a protein *v* is the maximum distance from *v* to all other proteins in graph *G*. The degree of centrality of protein *v* is the total number of adjacent proteins.

### 4.11. Plant Growth, Treatment with flg22, and Viral infection with TRV and CabLCV

All gene expression experiments used *Arabidopsis thaliana* ecotype Columbia (Col-0) at different ages. The seeds were germinated on half-strength Murashige and Skoog (MS; Sigma = Aldrich) plates containing 10% (*w*/*v*) sucrose and 0.8% (*w*/*v*) agar, sterile, and grown under normal growth conditions at 21 °C under a 16 h light/8 h dark cycle. After 10 days, the seedlings were transferred to a tissue culture plate containing 2 mL of 100 nM flg22 (Sigma-Aldrich), and incubated for 15 min [116]. For the viral infection assay with tobacco rattle virus (TRV), Agrobacterium cultures containing TRV-RNA1 (pTRV1) and TRV-RNA2 (pTRV2) T-DNA constructs were infiltrated onto the lower leaf of four-leaf stage *N. benthamiana* plants using a 1-mL needleless syringe. Infected leaves were confirmed by conventional RT-PCR using TRV-RNA2-specific primers. TRV was mechanically inoculated in *A. thaliana* grown in soil in a growth chamber for 14 days by rubbing the leaves with sap (0.05 M K2HPO4, pH 7.2, 0.01 M Na2SO3) from infected *N. benthamiana* leaves. After 2 weeks of inoculation, viral infection was confirmed by RT-PCR. For infection with cabbage leaf curl virus (CabLCV), plants at the seven-leaf stage were inoculated with plasmids containing partial tandem repeats of CabLCV DNA-A and DNA-B [117], using biolistic delivery as previously described [118,119]. Inoculated plants were transferred to a growth chamber, and infection was confirmed by conventional PCR using CabLCV DNA-B-specific primers.

### 4.12. RNA Extraction, Synthesis of cDNA, and qRT-PCR Analysis

For quantitative RT-PCR, total RNA was extracted from frozen leaves or seedlings with TRIzol (Invitrogen) according to the instructions from the manufacturer. To quantify flg22-induced expression, total RNA was extracted from a pool of 10 flg22-treated seedlings (as described in 4.11). For the TRV infection experiment, total RNA was extracted from a pool of 10 infected plants two weeks post-inoculation (as described in 4.11). For CabLCV infection, total RNA was extracted from a pool of 10 infected plants after 21 days of inoculation. To quantify gene expression in different organs, total RNA was extracted from flowers, the inflorescence axis, pedicels of 35 days-soil-grown Col-0 plants, and from roots of 10 days-grown plants in MS medium under the conditions described in 4.11. We used 3 samples of different pools of 10 plants each (therefore n = 3, biological replicates) and three technical replicates.

Total RNA was treated with 2 units of RNase-free DNase (Promega). First-strand cDNA was synthesized from 3.5 mg of total RNA using oligo-dT(18) and Transcriptase Reverse M-MLV (Invitrogen), according to the manufacturer’s instructions. Real-time RT-PCR reactions were performed on ABI7500 equipment (Applied Biosystems), using SYBR Green PCR Master Mix (Bio-rad). The amplification reactions were performed as follows: 2 min at 50 °C, 10 min at 95 °C, and 40 cycles of 94 °C for 15 s and 60 °C for 1 min. To quantify gene expression, we used the 2^−∆Ct^ method and actin 3 (At3g53750) as the endogenous control genes for data normalization.

## 5. Conclusions

An extensive family of RLKs and RLPs on the cell surface perceive external stimuli and allows communication of plant cells with the environment. Due to their conceptual relevance in cell signaling, RLKs have been extensively studied and characterized. In contrast, little is known about the RLP family that does not harbor conserved domains to prototype genome-wide searching and characterization of members in different plant species. As a result of this investigation, a new method, based on artificial intelligence and machine learning models in combination with Bayesian inference, designated RLPredictiOme, is proposed to perform genome-wide surveys of RLPs in plant species.

We provided evidence indicating that RLPredictiOme reliably predicts RLP subfamilies in plant genomes. First, the ML models achieved high accuracy, precision, sensitivity, and specificity for predicting RLPs with relatively high probability ranging from 0.79 to 0.99. Second, in the validation tests, more than 90% of known RLPs from Arabidopsis and rice were correctly predicted via RLPredictiOme. Finally, RLPredictiOme may have outperformed the predicting methods based on sequence comparison because it discovered new RLP subfamilies in the Arabidopsis genome. Therefore, PredctOme provides a reliable means to rationalize functional studies of the RLP gene family.

The new GDPDL-RLP subfamily seems to have expanded from the only GDPDL-RLK representative in the Arabidopsis genome. All five GDPDL-RLPs were expressed in different organs and responded to biotic signals. Evolution studies showed that their ectodomain may have undergone purifying selection, indicating that the members of this subfamily may have kept conserved functional signatures during evolution. In addition, an in silico analysis demonstrated that GDPDL-RLPs form biologically relevant hubs in the GDPDL-RLP-Arabidopsis protein-protein interactions network. Collectively, these biological studies confirmed the prediction of the new GDPDL-RLP subfamily.

In addition to using a set of conventional extractable features for training the classification models, RLPredictiOme also filters the conserved characteristics of the RLP configuration. These conserved attributes include the presence of a signal peptide, RLK ectodomains, a transmembrane segment, and the lack of a C-terminal kinase domain. Therefore, RLPredictiOme has the potential to predict RLPs from other organisms as well. Furthermore, the consistent and expanded results using RLPredctOme, which applies a different approach from sequence comparison methods, certify this new method as an innovative and promising tool for predicting RLPs. RLPredictOme will ultimately serve as an essential complement for protein annotation, identification, and functional prediction of novel RLPs in different plant species and organisms. 

## Figures and Tables

**Table 1:** Number of RLKs harboring the indicated ectodomain type.

Description	Total	Description	Total	Description	Total
LRR-RLK	14,087	CHASE-RLK	8	CUB-RLK	2
Unknown-RLK	10,020	Cysteine-rich-secretory-RLK	7	DUF1084-RLK	2
S-domain-RLK	3859	GDPDL-RLK	7	DUF726-RLK	2
Malectin-RLK	3299	Universal-stress-RLK	6	Endomembrane-RLK	2
Salt-stress-response/antifungal-RLK	2345	ACT-RLK	5	GAF-domain	2
L-Lectin-RLK	2213	Probable-lipid-transfer-RLK	5	GTPase-RLK	2
WAK-RLK	1844	Ankyrin-Kinase	4	Glycosyl hydrolases-RLK	2
B-lectin-RLK	549	Chromo-RLK	4	Glycosyltransferase-RLK	2
LysM-RLK	381	PAN-like-Kinase	4	HAD-RLK	2
WAK-EGF-RLK	285	PB1-RLK	4	HAD-hyrolase-like-RLK	2
EGF-like-RLK	212	Sel1-RLK	4	MSP-RLK	2
WAK-EFG-RLK	177	Alpha/beta-hydrolase-RLK	3	NB-ARC-RLK	2
RCC1-RLK	148	Cytochrome P450-RLK	3	PQQ-enzyme-RLK	2
B-Lectin-RLK	145	Helix-loop-helix-DNA-binding-RLK	3	Peptidase-RLK	2
PAN-RLK	131	Histidine-phosphatase-RLK	3	PfkB-RLK	2
C-Lectin-RLK	90	Major-Facilitator-RLK	3	Wnt-and-FGF-inhibitory-regulator-RLK	2
Glycosyl-hydrolases-RLK	90	MatE-RLK	3	Adenylate-cyclase-associated-(CAP)-N-terminal-RLK	1
Thaumatin-RLK	86	PPR	3	Alcohol-dehydrogenase-GroES-like-RLK	1
NAF-RLK	79	PPR-RLK	3	Aldose-1-epimerase-RLK	1
Ethylene-responsive-RLK	74	Phospholipase-RLK	3	Ankyrin-RLK	1
EF-hand-RLK	50	Proline-rich-RLK	3	Castor-and-Pollux-RLK	1
Cache-RLK	32	Sugar-(and other)-transporter-RLK	3	Cyclic nucleotide-binding-RLK	1
Chitinase-RLK	15	Transmembrane-RLK	3	Cyclic-nucleotide-binding-RLK	1
PAS-RLK	12	Alpha-amylase-catalytic-RLK	2	Cytochrome-P450-RLK	1
Plastocyanin-like-RLK	12	Barwin-RLK	2	DEAD/DEAH-box-helicase-RLK	1
Ring-finger-RLK	9	C2-RLK	2	DUF1221-RLK	1
Adenovirus E3-RLK	8				

**Table 2:** Subfamily size of receptor-like kinase proteins.

No	Label	Count
1	L-Lectin-RLK	980
2	LRR-RLK	5404
3	S-domain-RLK	1626
4	Malectin-RLK	1313
5	Salt-stress-response/antifungal-RLK	1004
6	WAK-RLK	1362
7	B-Lectin-RLK	362
8	Unknown-RLK	3285
10	PAN-RLK	41
11	Ethylene-responsive-RLK	29
12	Thaumatin-RLK	52
13	RCC1-RLK	65
14	Glycosyl-hydrolases-RLK	40
15	C-Lectin-RLK	21
16	Other-RLKs	192

**Table 3:** Summarized results of the evaluation models built with the RLPs/NRLPs datasets.

Data Set	Algorithm	ACC	F1	FDR	MCC	Precision	Sensitivity	Specificity
AAComposition_1	Logistic RegressionCV	0.9173	0.9211	0.0878	0.8343	0.9303	0.9303	0.9032
AAComposition_2	Logistic RegressionCV	0.9205	0.9241	0.0839	0.8407	0.9322	0.9322	0.9078
AAComposition_3	Logistic RegressionCV	0.9209	0.9245	0.0831	0.8416	0.9321	0.9321	0.9088
AAComposition_N_C terminal_1	MLP Classifier	0.9457	0.9478	0.0534	0.8912	0.9490	0.9490	0.9421
AAComposition_N_C terminal_2	MLP Classifier	0.9468	0.9487	0.0513	0.8934	0.9487	0.9487	0.9446
AAComposition_N_C terminal_3	MLP Classifier	0.9482	0.9499	0.0457	0.8964	0.9456	0.9456	0.9511
CPAASC_1	Linear Discriminant Analysis	0.9020	0.9102	0.1315	0.8074	0.9561	0.9561	0.8436
CPAASC_2	Linear Discriminant Analysis	0.9042	0.9120	0.1282	0.8116	0.9562	0.9562	0.8481
CPAASC_3	Linear Discriminant Analysis	0.9040	0.9119	0.1288	0.8113	0.9566	0.9566	0.8473
CPAASC_N_C terminal_1	Linear Discriminant Analysis	0.9104	0.9172	0.1183	0.8232	0.9558	0.9558	0.8614
CPAASC_N_C terminal_2	Linear Discriminant Analysis	0.9132	0.9196	0.1148	0.8284	0.9569	0.9569	0.8660
CPAASC_N_C terminal_3	Linear Discriminant Analysis	0.9140	0.9204	0.1137	0.8301	0.9572	0.9572	0.8674
Dipeptide_1	MLP Classifier	0.9439	0.9457	0.0497	0.8878	0.9412	0.9412	0.9468
Dipeptide_2	MLP Classifier	0.9481	0.9500	0.0501	0.8960	0.9500	0.9500	0.9459
Dipeptide_3	MLP Classifier	0.9447	0.9466	0.0497	0.8894	0.9428	0.9428	0.9468
Tripeptide_1	Logistic RegressionCV	0.9535	0.9551	0.0410	0.9069	0.9511	0.9511	0.9561
Tripeptide_2	Logistic RegressionCV	0.9550	0.9565	0.0389	0.9100	0.9519	0.9519	0.9584
Tripeptide_3	Logistic RegressionCV	0.9534	0.9549	0.0404	0.9067	0.9502	0.9502	0.9568
	Mean	0.9303	0.9342	0.0784	0.8615	0.9480	0.9480	0.9112

**Table 4:** Summarized results of the evaluation models built with the RLPs/RLKs datasets.

Data Set	Algorithm	ACC	F1	FDR	MCC	Precision	Sensitivity	Specificity
AAComposition_N_C terminal	Quadratic Discriminant Analysis	0.9775	0.9773	0.0337	0.9552	0.9884	0.9884	0.9670
Tripeptide	Gradient Boosting Classifier	0.9762	0.9760	0.0367	0.9527	0.9890	0.9890	0.9639
CPAASC_N_C_terminal	Linear Discriminant Analysis	0.9707	0.9706	0.0479	0.9421	0.9899	0.9899	0.9523
CPAASC	Linear Discriminant Analysis	0.9647	0.9647	0.0572	0.9304	0.9877	0.9877	0.9426
Dipeptide	MLP Classifier	0.9627	0.9617	0.0344	0.9254	0.9579	0.9579	0.9673
AAComposition	Quadratic Discriminant Analysis	0.9571	0.9571	0.0627	0.9151	0.9777	0.9777	0.9374
	Mean	0.9681	0.9679	0.0454	0.9368	0.9818	0.9818	0.9551

**Table 5:** Summarized results of the evaluation models built with the RLP subfamily datasets.

Data Set	Algorithm	ACC	F1	MCC	Precision	Sensitivity
AAComposition_10	Linear Discriminant Analysis	0.984	0.872	0.864	0.872	0.872
AAComposition_1	Calibrated ClassifierCV	0.984	0.869	0.861	0.869	0.869
AAComposition_2	Calibrated ClassifierCV	0.984	0.874	0.866	0.874	0.874
AAComposition_3	Linear Discriminant Analysis	0.984	0.873	0.864	0.873	0.873
AAComposition_4	Linear Discriminant Analysis	0.984	0.870	0.862	0.870	0.870
AAComposition_5	Linear Discriminant Analysis	0.983	0.867	0.858	0.867	0.867
AAComposition_6	Linear Discriminant Analysis	0.984	0.871	0.863	0.871	0.871
AAComposition_7	Calibrated ClassifierCV	0.984	0.869	0.861	0.869	0.869
AAComposition_8	Calibrated ClassifierCV	0.985	0.876	0.868	0.876	0.876
AAComposition_9	Linear Discriminant Analysis	0.984	0.875	0.867	0.875	0.875
	Mean	0.984	0.872	0.863	0.872	0.872
AAComposition_N_C_terminal_10	Calibrated ClassifierCV	0.989	0.911	0.905	0.911	0.911
AAComposition_N_C_terminal_1	Calibrated ClassifierCV	0.988	0.904	0.897	0.904	0.904
AAComposition_N_C_terminal_2	Calibrated ClassifierCV	0.989	0.908	0.902	0.908	0.908
AAComposition_N_C_terminal_3	Calibrated ClassifierCV	0.988	0.902	0.896	0.902	0.902
AAComposition_N_C_terminal_4	KNeighbors Classifier	0.989	0.911	0.905	0.911	0.911
AAComposition_N_C_terminal_5	KNeighbors Classifier	0.989	0.909	0.903	0.909	0.909
AAComposition_N_C_terminal_6	KNeighbors Classifier	0.988	0.903	0.896	0.903	0.903
AAComposition_N_C_terminal_7	KNeighbors Classifier	0.988	0.900	0.894	0.900	0.900
AAComposition_N_C_terminal_8	Calibrated ClassifierCV	0.988	0.903	0.897	0.903	0.903
AAComposition_N_C_terminal_9	Calibrated ClassifierCV	0.988	0.907	0.900	0.907	0.907
	Mean	0.988	0.906	0.899	0.906	0.906
CPAASC_10	Linear Discriminant Analysis	0.972	0.778	0.764	0.778	0.778
CPAASC_1	AdaBoost Classifier	0.971	0.772	0.757	0.772	0.772
CPAASC_2	AdaBoost Classifier	0.972	0.776	0.761	0.776	0.776
CPAASC_3	AdaBoost Classifier	0.972	0.773	0.759	0.773	0.773
CPAASC_4	Linear Discriminant Analysis	0.971	0.770	0.755	0.770	0.770
CPAASC_5	Linear Discriminant Analysis	0.972	0.773	0.759	0.773	0.773
CPAASC_6	Linear Discriminant Analysis	0.971	0.771	0.756	0.771	0.771
CPAASC_7	AdaBoos tClassifier	0.972	0.773	0.758	0.773	0.773
CPAASC_8	Linear Discriminant Analysis	0.972	0.778	0.763	0.778	0.778
CPAASC_9	AdaBoost Classifier	0.972	0.774	0.759	0.774	0.774
	Mean	0.972	0.774	0.759	0.774	0.774
CPAASC_N_C_terminal_10	AdaBoost Classifier	0.975	0.800	0.787	0.800	0.800
CPAASC_N_C_terminal_1	Linear Discriminant Analysis	0.976	0.810	0.797	0.810	0.810
CPAASC_N_C_terminal_2	AdaBoost Classifier	0.975	0.803	0.790	0.803	0.803
CPAASC_N_C_terminal_3	Linear Discriminant Analysis	0.976	0.804	0.792	0.804	0.804
CPAASC_N_C_terminal_4	Linear Discriminant Analysis	0.976	0.805	0.793	0.805	0.805
CPAASC_N_C_terminal_5	AdaBoost Classifier	0.975	0.802	0.789	0.802	0.802
CPAASC_N_C_terminal_6	Linear Discriminant Analysis	0.976	0.808	0.795	0.808	0.808
CPAASC_N_C_terminal_7	Linear Discriminant Analysis	0.976	0.808	0.795	0.808	0.808
CPAASC_N_C_terminal_8	AdaBoost Classifier	0.975	0.802	0.789	0.802	0.802
CPAASC_N_C_terminal_9	Linear Discriminant Analysis	0.976	0.805	0.792	0.805	0.805
	Mean	0.976	0.805	0.792	0.805	0.805
Dipeptide_10	KNeighbors Classifier	0.992	0.935	0.931	0.935	0.935
Dipeptide_1	KNeighbors Classifier	0.992	0.937	0.933	0.937	0.937
Dipeptide_2	KNeighbors Classifier	0.992	0.935	0.931	0.935	0.935
Dipeptide_3	KNeighbors Classifier	0.992	0.934	0.930	0.934	0.934
Dipeptide_4	KNeighbors Classifier	0.991	0.932	0.927	0.932	0.932
Dipeptide_5	KNeighbors Classifier	0.992	0.934	0.930	0.934	0.934
Dipeptide_6	KNeighbors Classifier	0.991	0.931	0.926	0.931	0.931
Dipeptide_7	KNeighbors Classifier	0.992	0.933	0.929	0.933	0.933
Dipeptide_8	KNeighbors Classifier	0.991	0.925	0.920	0.925	0.925
Dipeptide_9	KNeighbors Classifier	0.991	0.929	0.925	0.929	0.929
	Mean	0.992	0.932	0.928	0.932	0.932
Tripeptide_1	KNeighbors Classifier	0.995	0.957	0.954	0.957	0.957
Tripeptide_2	KNeighbors Classifier	0.994	0.955	0.952	0.955	0.955
Tripeptide_3	KNeighbors Classifier	0.994	0.956	0.953	0.956	0.956
Tripeptide_4	KNeighbors Classifier	0.995	0.958	0.955	0.958	0.958
Tripeptide_5	KNeighbors Classifier	0.995	0.958	0.955	0.958	0.958
Tripeptide_6	KNeighbors Classifier	0.994	0.954	0.951	0.954	0.954
Tripeptide_7	KNeighbors Classifier	0.994	0.955	0.952	0.955	0.955
Tripeptide_8	KNeighbors Classifier	0.994	0.951	0.948	0.951	0.951
Tripeptide_9	KNeighbors Classifier	0.995	0.958	0.955	0.958	0.958
Tripeptide_10	KNeighbors Classifier	0.995	0.959	0.957	0.959	0.959
	Mean	0.994	0.956	0.953	0.956	0.956

**Table 6:** Validation of the almost characterized RLPs.

Accession	SP	TM	RLP-NRLP	RLP-NRLP Probability	RLP-RLK	RLP-RLK Probability	RLP-Subfamily	RLP-Subfamily Probability	Classification	Decision Probability
NP_001234733.2	Y	Y	RLP	0.9961	RLP	0.5751	LRR-RLP	0.7666	(LRR-RLP)	0.9894
sQ9LNV9.2_RLP1	Y	Y	RLP	0.9961	RLP	0.7161	LRR-RLP	0.7671	(LRR-RLP)	0.9891
sp—Q93ZH0.1—LYM1	Y	Y	RLP	0.8941	RLP	0.9915	LysM-RLP	0.467	(LysM-RLP)	0.989
CAC40826.1_HcrVf2	Y	Y	RLP	0.9961	RLP	0.9895	LRR-RLP	0.8333	(LRR-RLP)	0.9888
AAA65235.1_Cf-9	Y	Y	RLP	0.9965	RLP	0.9906	LRR-RLP	0.8331	(LRR-RLP)	0.9887
AAC78594.1_Hcr2-2A	Y	Y	RLP	0.9965	RLP	0.8569	LRR-RLP	0.849	(LRR-RLP)	0.9885
Q9SSD1.1	Y	Y	RLP	0.9966	RLP	0.991	LRR-RLP	0.4667	(LRR-RLP)	0.9885
AAC15779.1_Cf-2.1	Y	Y	RLP	0.9965	RLP	0.855	LRR-RLP	0.85	(LRR-RLP)	0.9882
sp—Q7FZR1.1—RLP52	Y	Y	RLP	0.9966	RLP	0.9903	LRR-RLP	0.8336	(LRR-RLP)	0.9882
QED40966.1	Y	Y	RLP	0.9962	RLP	0.7168	LRR-RLP	0.8506	(LRR-RLP)	0.9881
CAC40827.1_HcrVf3	Y	Y	RLP	0.9964	RLP	0.9909	LRR-RLP	0.8501	(LRR-RLP)	0.988
sp—Q9LJS0.1—RLP42	Y	Y	RLP	0.9966	RLP	0.9911	LRR-RLP	0.8502	(LRR-RLP)	0.988
AAC78593.1_Hcr2-0B	Y	Y	RLP	0.9962	RLP	0.991	LRR-RLP	0.8495	(LRR-RLP)	0.9879
Q9FK66.1_RLP55	Y	Y	RLP	0.9958	RLP	0.9915	LRR-RLP	0.6669	(LRR-RLP)	0.9879
sQ9SN38.1_RLP5	Y	Y	RLP	0.9963	RLP	0.9912	LRR-RLP	0.8497	(LRR-RLP)	0.9879
AAC78596.1_Hcr2-5D	Y	Y	RLP	0.9959	RLP	0.9909	LRR-RLP	0.85	(LRR-RLP)	0.9878
BAE95828.1 (LysM)	Y	Y	RLP	0.9964	RLP	0.99	Undefined	0.4169	(Undefined)	0.9878
Q9LJS2.1	Y	Y	RLP	0.9964	RLP	0.9906	LRR-RLP	0.8505	(LRR-RLP)	0.9878
AJG42080.1_RLM2	Y	Y	RLP	0.9963	RLP	0.9908	LRR-RLP	0.8493	(LRR-RLP)	0.9877
CAA05269.1_Hcr9-4E	Y	Y	RLP	0.9962	RLP	0.9893	LRR-RLP	0.8332	(LRR-RLP)	0.9877
AJG42091.1_LEPR3	Y	Y	RLP	0.9967	RLP	0.9911	LRR-RLP	0.8508	(LRR-RLP)	0.9875
Q9M2Y3.1_RLP44	Y	Y	RLP	0.9962	RLP	0.9902	LRR-RLP	0.7503	(LRR-RLP)	0.9875
CAC40825.1_HcrVf1	Y	Y	RLP	0.9965	RLP	0.9921	LRR-RLP	0.8166	(LRR-RLP)	0.9874
NP_001234474.2	Y	Y	RLP	0.9963	RLP	0.991	LRR-RLP	0.8332	(LRR-RLP)	0.9874
Solyc08g016270.1.1	Y	Y	RLP	0.9961	RLP	0.72	LRR-RLP	0.6335	(LRR-RLP)	0.9874
AAC78595.1_Hcr2-5B	Y	Y	RLP	0.9963	RLP	0.8517	LRR-RLP	0.85	(LRR-RLP)	0.9873
O80809.1_CLV2	Y	Y	RLP	0.9964	RLP	0.991	LRR-RLP	0.8496	(LRR-RLP)	0.9873
sp—O23006.1—LYM2	Y	Y	RLP	0.9962	RLP	0.9908	Undefined	0.5005	(Undefined)	0.9873
sp—O48849.1—RLP23	Y	Y	RLP	0.9959	RLP	0.9906	LRR-RLP	0.7833	(LRR-RLP)	0.9873
AAC78592.1_Hcr2-0A	Y	Y	RLP	0.9966	RLP	0.8518	LRR-RLP	0.8513	(LRR-RLP)	0.9872
sp—Q6NPN4.1—LYM3	Y	Y	RLP	0.9452	RLP	0.99	LysM-RLP	0.4501	(LysM-RLP)	0.9872
AAC78591.1	Y	Y	RLP	0.9966	RLP	0.9899	LRR-RLP	0.8507	(LRR-RLP)	0.9871
AJV90937.1	Y	Y	RLP	0.9968	RLP	0.8507	LRR-RLP	0.8332	(LRR-RLP)	0.9871
AUT14025.1	Y	Y	RLP	0.9962	RLP	0.8537	LRR-RLP	0.7329	(LRR-RLP)	0.987
AAC15780.1_Cf-2.2	Y	Y	RLP	0.9961	RLP	0.8555	LRR-RLP	0.8491	(LRR-RLP)	0.9863
AGI92782.1_RLP1.813	Y	Y	RLP	0.9963	RLP	0.9906	LRR-RLP	0.4005	(LRR-RLP)	0.9862
NP_187187.1	Y	Y	RLP	0.9964	RLP	0.9913	LRR-RLP	0.6497	(LRR-RLP)	0.986
AKR80573.1_I-7	Y	Y	RLP	0.9963	RLP	0.8605	LRR-RLP	0.65	(LRR-RLP)	0.9855
NP_001362850.1_EIX2	Y	Y	RLP	0.9961	RLP	0.8581	LRR-RLP	0.6005	(LRR-RLP)	0.985
sp—Q9SHI4.1—RLP3	N	Y	RLP	0.9965	RLP	0.9904	LRR-RLP	0.8328	(LRR-RLP)	0.8015
NP_001355132.1	N	Y	RLP	0.9965	RLP	0.9903	LRR-RLP	0.5163	(LRR-RLP)	0.8012
Q940E8.1_FEA2	Y	N	RLP	0.9487	RLP	0.8554	LRR-RLP	0.849	NRLP	0.2048
sp—Q67UE8.1—LYP4	Y	N	RLP	0.7894	RLP	0.8564	Undefined	0.0	NRLP	0.2017
AFB75328.1	Y	N	RLP	0.9472	RLP	0.857	LRR-RLP	0.5667	NRLP	0.2012
AKP45167.1	Y	N	RLP	0.9462	RLP	0.8543	Undefined	0.4495	NRLP	0.201
sp—Q69T51.1—LYP6	Y	N	RLP	0.8422	RLP	0.8544	Undefined	0.0	NRLP	0.2007
LOC_Os04g56430.1	Y	N	RLP	0.9471	RLP	0.8518	Salt-stress-response/antifungal-RLP	0.4334	NRLP	0.1986

**Table 7:** Validation of the RLPs from the genome-wide study of Arabidopsis RLPs restricted to the LRR-RLP subfamily.

Accession	SP	TM	RLP-NRLP	RLP-NRLP Probability	RLP-RLK	RLP-RLK Probability	RLP-Subfamily	RLP-Subfamily Probability	Classification	Decision Probability
AT1G65380.1	Y	Y	RLP	0.9962	RLP	0.9907	LRR-RLP	0.8505	(LRR-RLP)	0.9902
AT1G17240.1	Y	Y	RLP	0.9962	RLP	0.9913	LRR-RLP	0.8497	(LRR-RLP)	0.9886
AT4G13880.1	Y	Y	RLP	0.9963	RLP	0.9899	LRR-RLP	0.8001	(LRR-RLP)	0.9884
AT5G27060.1	Y	Y	RLP	0.9962	RLP	0.991	LRR-RLP	0.6669	(LRR-RLP)	0.9884
AT3G23110.1	Y	Y	RLP	0.9964	RLP	0.9912	LRR-RLP	0.6502	(LRR-RLP)	0.9883
AT1G80080.1	Y	Y	RLP	0.9961	RLP	0.9911	LRR-RLP	0.5506	(LRR-RLP)	0.9883
AT2G32680.1	Y	Y	RLP	0.9967	RLP	0.9918	LRR-RLP	0.7838	(LRR-RLP)	0.9882
AT1G74180.1	Y	Y	RLP	0.9959	RLP	0.858	LRR-RLP	0.8163	(LRR-RLP)	0.988
AT3G05370.1	Y	Y	RLP	0.9962	RLP	0.8556	LRR-RLP	0.6337	(LRR-RLP)	0.988
AT3G11080.1	Y	Y	RLP	0.9962	RLP	0.991	LRR-RLP	0.8496	(LRR-RLP)	0.988
AT3G28890.1	Y	Y	RLP	0.9966	RLP	0.8561	LRR-RLP	0.6336	(LRR-RLP)	0.988
AT2G25440.1	Y	Y	RLP	0.9962	RLP	0.9902	LRR-RLP	0.4832	(LRR-RLP)	0.9878
AT5G45770.1	Y	Y	RLP	0.9965	RLP	0.99	LRR-RLP	0.683	(LRR-RLP)	0.9878
AT2G42800.1	Y	Y	RLP	0.9963	RLP	0.9908	LRR-RLP	0.6665	(LRR-RLP)	0.9876
AT3G05360.1	Y	Y	RLP	0.9967	RLP	0.9913	LRR-RLP	0.6668	(LRR-RLP)	0.9876
AT5G65830.1	Y	Y	RLP	0.9966	RLP	0.8566	LRR-RLP	0.667	(LRR-RLP)	0.9876
AT1G28340.1	Y	Y	RLP	0.8425	RLP	0.9905	Malectin-RLP	0.4502	(Malectin-RLP)	0.9875
AT1G74190.1	Y	Y	RLP	0.9959	RLP	0.8564	LRR-RLP	0.8499	(LRR-RLP)	0.9871
AT2G15080.1	Y	Y	RLP	0.9965	RLP	0.9904	LRR-RLP	0.8502	(LRR-RLP)	0.987
AT3G05650.1	Y	Y	RLP	0.9964	RLP	0.9906	LRR-RLP	0.6664	(LRR-RLP)	0.9868
AT1G45616.1	Y	Y	RLP	0.9961	RLP	0.9913	LRR-RLP	0.7665	(LRR-RLP)	0.9868
AT3G05660.1	Y	Y	RLP	0.9966	RLP	0.8557	LRR-RLP	0.85	(LRR-RLP)	0.9866
AT1G58190.1	Y	Y	RLP	0.9962	RLP	0.8521	LRR-RLP	0.6663	(LRR-RLP)	0.9866
AT3G49750.1	Y	Y	RLP	0.9963	RLP	0.9909	LRR-RLP	0.7502	(LRR-RLP)	0.9865
AT4G13920.1	Y	Y	RLP	0.9967	RLP	0.9911	LRR-RLP	0.8498	(LRR-RLP)	0.9865
AT5G25910.1	Y	Y	RLP	0.9964	RLP	0.9899	LRR-RLP	0.8501	(LRR-RLP)	0.9864
AT2G33060.1	Y	Y	RLP	0.9966	RLP	0.9914	LRR-RLP	0.8332	(LRR-RLP)	0.9863
AT4G04220.1	Y	Y	RLP	0.9962	RLP	0.9911	LRR-RLP	0.8506	(LRR-RLP)	0.9863
AT2G33050.1	Y	Y	RLP	0.9964	RLP	0.9915	LRR-RLP	0.7498	(LRR-RLP)	0.986
AT1G71400.1	Y	Y	RLP	0.996	RLP	0.8563	LRR-RLP	0.6831	(LRR-RLP)	0.9851
AT4G18760.1	Y	Y	RLP	0.9967	RLP	0.9903	LRR-RLP	0.8495	(LRR-RLP)	0.9885
AT1G71390.1	N	Y	RLP	0.9966	RLP	0.99	LRR-RLP	0.6667	(LRR-RLP)	0.8021
AT2G25470.1	N	Y	RLP	0.9964	RLP	0.8556	LRR-RLP	0.8502	(LRR-RLP)	0.8014
AT1G47890.1	N	Y	RLP	0.9967	RLP	0.9908	LRR-RLP	0.8501	(LRR-RLP)	0.8001
AT4G13810.1	N	Y	RLP	0.9964	RLP	0.9907	LRR-RLP	0.833	(LRR-RLP)	0.7997
AT3G23010.1	N	Y	RLP	0.9965	RLP	0.9908	LRR-RLP	0.667	(LRR-RLP)	0.7995
AT1G74170.1	N	Y	RLP	0.9964	RLP	0.8561	LRR-RLP	0.7164	(LRR-RLP)	0.7994
AT3G24982.1	N	Y	RLP	0.9963	RLP	0.989	LRR-RLP	0.8512	(LRR-RLP)	0.7993
AT1G17250.1	N	Y	RLP	0.9965	RLP	0.9911	LRR-RLP	0.8496	(LRR-RLP)	0.799
AT3G23120.1	N	Y	RLP	0.997	RLP	0.9905	LRR-RLP	0.6835	(LRR-RLP)	0.7976
AT3G53240.1	N	Y	RLP	0.9961	RLP	0.9905	LRR-RLP	0.783	(LRR-RLP)	0.7973
AT1G07390.1	N	Y	RLP	0.9957	RLP	0.7119	LRR-RLP	0.7826	(LRR-RLP)	0.7969
AT3G11010.1	N	Y	RLP	0.9961	RLP	0.9902	LRR-RLP	0.6665	(LRR-RLP)	0.7958
AT1G34290.1	Y	Y	RLP	0.9964	RLP	0.9898	Undefined	0.2166	(Undefined)	0.7949
AT5G49290.1	N	Y	RLP	0.9966	RLP	0.9901	LRR-RLP	0.6833	(LRR-RLP)	0.7941
AT2G32660		N								
AT2G33020		N								
AT2G33030		N								
AT2G33080		N								
AT3G24900		N								
AT3G25010		N								
AT4G13900		N								
AT5G40170		N								
AT3G25020		N								

**Table 8:** Random sequences confronted against RLPredictiOme.

Accession	SP	TM	RLP-NRLP	RLP-NRLP Probability	RLP-RLK	RLP-RLK Probability	RLP-Subfamily	RLP-Subfamily Probability	Classification	Decision Probability
Alien_71_464	Y	Y	NRLP	0.0532	RLP	0.7145	Other-RLP	0.4166	NRLP	0.4033
Alien_78_801	Y	Y	NRLP	0.0532	RLP	0.857	WAK-RLP	0.3169	NRLP	0.4014
Alien_88_471	N	Y	NRLP	0.369	RLP	0.855	Unknown	0.2837	NRLP	0.2068
Alien_90_956	N	Y	NRLP	0.0527	RLK-like	0.5721	Other-RLP	0.3499	NRLP	0.2064
Alien_94_666	N	Y	NRLP	0.0535	RLP	0.8558	S-domain-RLP	0.3164	NRLP	0.2045
Alien_11_789	N	Y	NRLP	0.0524	RLK-like	0.4288	Other-RLP	0.4331	NRLP	0.2034
Alien_34_248	N	Y	NRLP	0.2093	RLP	0.8571	Other-RLP	0.4004	NRLP	0.2022
Alien_70_660	N	Y	NRLP	0.3677	RLP	0.8564	Unknown	0.2491	NRLP	0.2002
Alien_59_959	N	Y	NRLP	0.052	RLK-like	0.576	S-domain-RLP	0.417	NRLP	0.1994
Alien_20_195	Y	N	NRLP	0.3704	RLP	0.8544	Unknown	0.2671	NRLP	0.1987
Alien_23_503	N	Y	NRLP	0.3698	RLP	0.8596	Unknown	0.3	NRLP	0.1987
Alien_69_854	N	Y	NRLP	0.0542	RLP	0.7198	Other-RLP	0.4327	NRLP	0.1985
Alien_2_750	N	Y	NRLP	0.0526	RLK-like	0.5768	Other-RLP	0.3331	NRLP	0.1956
Alien_66_528	N	N	NRLP	0.0001	RLP	0.8549	S-domain-RLP	0.3829	NRLP	0.0195
Alien_1_268	N	N	NRLP	0.0002	RLP	0.8536	Other-RLP	0.3831	NRLP	0.0093
Alien_51_917	N	N	NRLP	0.0002	RLK-like	0.573	Unknown	0.283	NRLP	0.0044
Alien_79_429	N	N	NRLP	0.3166	RLP	0.8588	Other-RLP	0.3001	NRLP	0.0041
Alien_61_779	N	N	NRLP	0.0002	RLP	0.7131	S-domain-RLP	0.3834	NRLP	0.0036
Alien_67_112	N	N	NRLP	0.1591	RLP	0.7131	Other-RLP	0.3342	NRLP	0.0035
Alien_42_363	N	N	NRLP	0.316	RLP	0.8576	S-domain-RLP	0.3336	NRLP	0.003
Alien_4_417	N	N	NRLP	0.0002	RLK-like	0.5712	WAK-RLP	0.4337	NRLP	0.0029
Alien_24_102	N	N	NRLP	0.4222	RLP	0.861	WAK-RLP	0.3498	NRLP	0.0027
Alien_9_882	N	N	NRLP	0.0002	RLP	0.7132	S-domain-RLP	0.3664	NRLP	0.0019
Alien_7_199	N	N	NRLP	0.3166	RLP	0.8564	WAK-RLP	0.3504	NRLP	0.0018
Alien_29_460	N	N	NRLP	0.2089	RLP	0.8554	Unknown	0.284	NRLP	0.0017
Alien_50_474	N	N	NRLP	0.0009	RLP	0.8548	Unknown	0.2495	NRLP	0.0017
Alien_72_442	N	N	NRLP	0.0002	RLP	0.8498	Unknown	0.2333	NRLP	0.0017
Alien_97_120	N	N	NRLP	0.3685	RLP	0.8566	Unknown	0.2999	NRLP	0.0017
Alien_38_893	N	N	NRLP	0.0003	RLK-like	0.5771	S-domain-RLP	0.4499	NRLP	0.0016
Alien_73_528	N	N	NRLP	0.0002	RLP	0.857	S-domain-RLP	0.3665	NRLP	0.0016
Alien_83_641	N	N	NRLP	0.0003	RLP	0.7085	Other-RLP	0.3502	NRLP	0.0016
Alien_44_248	N	N	NRLP	0.0003	RLP	0.7133	S-domain-RLP	0.3833	NRLP	0.0015
Alien_62_945	N	N	NRLP	0.0002	RLK-like	0.5733	S-domain-RLP	0.4834	NRLP	0.0015
Alien_16_855	N	N	NRLP	0.0002	RLK-like	0.4308	Unknown	0.2658	NRLP	0.0014
Alien_40_703	N	N	NRLP	0.0002	RLP	0.711	S-domain-RLP	0.3499	NRLP	0.0014
Alien_45_534	N	N	NRLP	0.0002	RLP	0.8553	WAK-RLP	0.3165	NRLP	0.0014
Alien_74_665	N	N	NRLP	0.0001	RLP	0.8547	Unknown	0.2503	NRLP	0.0014
Alien_18_925	N	N	NRLP	0.0001	RLK-like	0.5679	Other-RLP	0.4166	NRLP	0.0013
Alien_33_955	N	N	NRLP	0.0003	RLK-like	0.4348	Unknown	0.2332	NRLP	0.0013
Alien_39_171	N	N	NRLP	0.1577	RLP	0.8516	Unknown	0.2665	NRLP	0.0012
Alien_49_350	N	N	NRLP	0.0002	RLP	0.8573	S-domain-RLP	0.4842	NRLP	0.0012
Alien_63_622	N	N	NRLP	0.0002	RLP	0.8555	Unknown	0.2664	NRLP	0.0012
Alien_89_627	N	N	NRLP	0.0002	RLP	0.8567	Other-RLP	0.3835	NRLP	0.0012
Alien_91_929	N	N	NRLP	0.0003	RLK-like	0.573	Other-RLP	0.4331	NRLP	0.0012
Alien_14_450	N	N	NRLP	0.3148	RLP	0.7157	WAK-RLP	0.333	NRLP	0.0011
Alien_15_536	N	N	NRLP	0.0007	RLP	0.8566	Unknown	0.2668	NRLP	0.0011
Alien_22_586	N	N	NRLP	0.001	RLP	0.8562	S-domain-RLP	0.3993	NRLP	0.0011
Alien_3_226	N	N	NRLP	0.0003	RLK-like	0.431	Unknown	0.2991	NRLP	0.0011
Alien_57_326	N	N	NRLP	0.3151	RLP	0.8605	Unknown	0.2502	NRLP	0.0011
Alien_13_137	N	N	NRLP	0.2113	RLK-like	0.5764	Unknown	0.1667	NRLP	0.001
Alien_35_659	N	N	NRLP	0.0002	RLK-like	0.5687	Other-RLP	0.3829	NRLP	0.001
Alien_37_440	N	N	NRLP	0.0003	RLK-like	0.5743	Unknown	0.2666	NRLP	0.001
Alien_48_571	N	N	NRLP	0.0002	RLP	0.8586	Unknown	0.2999	NRLP	0.001
Alien_54_839	N	N	NRLP	0.0004	RLP	0.7158	Unknown	0.2674	NRLP	0.001
Alien_12_553	N	N	NRLP	0.3185	RLP	0.858	Unknown	0.2335	NRLP	0.0009
Alien_17_304	N	N	NRLP	0.3169	RLP	0.8541	Unknown	0.2828	NRLP	0.0009
Alien_25_176	N	N	NRLP	0.0003	RLP	0.8568	Unknown	0.2667	NRLP	0.0009
Alien_30_623	N	N	NRLP	0.0002	RLP	0.8547	Other-RLP	0.3833	NRLP	0.0009
Alien_32_240	N	N	NRLP	0.1576	RLP	0.8531	Unknown	0.2499	NRLP	0.0009
Alien_53_589	N	N	NRLP	0.0006	RLP	0.7103	Unknown	0.3	NRLP	0.0009
Alien_58_715	N	N	NRLP	0.0001	RLK-like	0.5748	S-domain-RLP	0.3842	NRLP	0.0009
Alien_82_456	N	N	NRLP	0.0001	RLP	0.855	S-domain-RLP	0.3165	NRLP	0.0009
Alien_85_415	N	N	NRLP	0.0004	RLP	0.715	Unknown	0.2167	NRLP	0.0009
Alien_8_947	N	N	NRLP	0.0001	RLK-like	0.5689	Unknown	0.25	NRLP	0.0009
Alien_10_555	N	N	NRLP	0.0002	RLP	0.8536	Unknown	0.2996	NRLP	0.0008
Alien_19_229	N	N	NRLP	0.0003	RLP	0.8599	PAN-RLP	0.3336	NRLP	0.0008
Alien_27_824	N	N	NRLP	0.0002	RLP	0.7111	Unknown	0.3337	NRLP	0.0008
Alien_41_731	N	N	NRLP	0.0004	RLP	0.7117	Unknown	0.2666	NRLP	0.0008
Alien_43_686	N	N	NRLP	0.0001	RLP	0.7129	S-domain-RLP	0.3662	NRLP	0.0008
Alien_47_420	N	N	NRLP	0.0004	RLP	0.8546	Other-RLP	0.4172	NRLP	0.0008
Alien_52_779	N	N	NRLP	0.0003	RLK-like	0.4383	Unknown	0.2999	NRLP	0.0008
Alien_55_478	N	N	NRLP	0.0002	RLP	0.7179	Other-RLP	0.3997	NRLP	0.0008
Alien_60_817	N	N	NRLP	0.0002	RLP	0.7135	Unknown	0.2999	NRLP	0.0008
Alien_64_626	N	N	NRLP	0.0002	RLP	0.7138	Other-RLP	0.4	NRLP	0.0008
Alien_75_673	N	N	NRLP	0.0002	RLP	0.8548	Unknown	0.2832	NRLP	0.0008
Alien_81_442	N	N	NRLP	0.0003	RLK-like	0.5736	S-domain-RLP	0.4833	NRLP	0.0008
Alien_87_495	N	N	NRLP	0.0005	RLP	0.8555	S-domain-RLP	0.3838	NRLP	0.0008
Alien_93_110	N	N	NRLP	0.3149	RLP	0.8597	WAK-RLP	0.467	NRLP	0.0008
Alien_99_622	N	N	NRLP	0.0002	RLP	0.8568	Unknown	0.25	NRLP	0.0008
Alien_21_499	N	N	NRLP	0.0002	RLP	0.86	S-domain-RLP	0.3498	NRLP	0.0007
Alien_31_429	N	N	NRLP	0.0002	RLP	0.7128	Unknown	0.2996	NRLP	0.0007
Alien_46_860	N	N	NRLP	0.0002	RLK-like	0.571	Unknown	0.2995	NRLP	0.0007
Alien_56_859	N	N	NRLP	0.0005	RLK-like	0.5724	S-domain-RLP	0.3328	NRLP	0.0007
Alien_5_855	N	N	NRLP	0.0003	RLK-like	0.572	Unknown	0.2997	NRLP	0.0007
Alien_65_609	N	N	NRLP	0.0002	RLK-like	0.4257	Unknown	0.2667	NRLP	0.0007
Alien_6_529	N	N	NRLP	0.0001	RLP	0.8565	Unknown	0.2504	NRLP	0.0007
Alien_86_232	N	N	NRLP	0.1581	RLP	0.8535	Other-RLP	0.3495	NRLP	0.0007
Alien_92_960	N	N	NRLP	0.0005	RLK-like	0.5741	Other-RLP	0.3168	NRLP	0.0007
Alien_95_597	N	N	NRLP	0.157	RLP	0.8588	Unknown	0.2833	NRLP	0.0007
Alien_96_597	N	N	NRLP	0.3704	RLP	0.8544	WAK-RLP	0.3999	NRLP	0.0007
Alien_0_119	N	N	NRLP	0.0528	RLP	0.7163	PAN-RLP	0.4339	NRLP	0.0006
Alien_26_112	N	N	NRLP	0.5285	RLP	0.8585	Unknown	0.2664	NRLP	0.0006
Alien_76_327	N	N	NRLP	0.0003	RLP	0.7066	Other-RLP	0.4002	NRLP	0.0006
Alien_77_685	N	N	NRLP	0.0002	RLK-like	0.569	Unknown	0.2494	NRLP	0.0006
Alien_98_323	N	N	NRLP	0.1046	RLP	0.7172	Other-RLP	0.5328	NRLP	0.0006
Alien_28_468	N	N	NRLP	0.0001	RLP	0.8563	Unknown	0.2831	NRLP	0.0005
Alien_36_821	N	N	NRLP	0.0001	RLP	0.717	Unknown	0.2337	NRLP	0.0005
Alien_68_626	N	N	NRLP	0.0002	RLP	0.8541	Unknown	0.2835	NRLP	0.0005
Alien_80_637	N	N	NRLP	0.0002	RLK-like	0.5715	S-domain-RLP	0.4333	NRLP	0.0005
Alien_84_494	N	N	NRLP	0.1614	RLP	0.8574	S-domain-RLP	0.3501	NRLP	0.0005

**Table 9:** Number of RLPs and predicted RLKs.

Class (Subfamily)	RLP	Correctly Classified *	Unknown Function **	Incorrectly Subfamily Classified ***	Mistakenly Classified ****	RLKs in Arabidopsis
LRR-RLP	49	46	3	0	2	235
L-Lectin-RLP	5	0	5		5	45
Salt stress response/antifungal-RLP	9	3	1	5	0	44
WAK-RLP	6	5	1		4	42
S-domain-RLP	1	1			1	37
Unknown-RLP (Extensin, PERK, RKF3, URKI)	43	43			11	28
Malectin-RLP	6	2	3	1	5	15
RCC1-RLP	4		4			8
LysM-RLP	4	2	2			3
B-lectin-RLP	1			1		2
C-Lectin-RLP	0					2
Ethylene-responsive-RLP	3	3			3	2
PAS-RLP	0					2
Thaumatin-RLP	6	6				2
PPR-RLP	0					1
Glycosyl-hydrolases-RLP	3		3			0
PAN-RLP	1		1		1	0
Other-RLP	35	11	24		13	0
Undefined	78					
Total	176	122	47	7	45	468

* Correctly classified as shown in Table S2 in black bold. ** Unknown function as shown in Table S2 in red. *** Incorrectly subfamily classified as shown in Table S2 in blue. **** Mistakes as shown in Table S2 in standard black.

**Table 10:** Molecular evolution analysis of the GDPDLs.

Sequence	Ka	Ks	Ka/Ks	Selection	Date (Mya)	*p*-Value
GDPDL5-GDPDL3	0.382	1.578	0.242	Purifying	129.316	7.98 × 10^−49^
GDPD (ectodomain)- GDPDL4	0.214	1.466	0.146	Purifying	120.193	2.22 × 10-^45^
GDPDL4-GDPD-RLK	0.214	1.288	0.166	Purifying	105.602	9.31 × 10^−45^
GDPDL1-GDPDL4	0.180	0.940	0.192	Purifying	77.037	1.60 × 10^−51^
GDPDL3-GDPDL4	0.164	0.852	0.192	Purifying	69.822	1.12 × 10^−46^
GDPDL4-GDPDL6	0.646	0.802	0.805	Purifying	65.744	0.146094
GDPD-RLK-GDPDL6	0.695	0.638	1.090	Positive	52.286	0.109708
GDPD (ectodomain)- GDPDL3	0.170	0.397	0.428	Purifying	32.525	4.56 × 10^−13^
GDPDL3-GDPD-RLK	0.167	0.394	0.423	Purifying	32.333	3.06 × 10^−13^
GDPD-RLK-GDPDL3	0.167	0.394	0.423	Purifying	32.333	3.06 × 10^−13^
GDPDL1-GDPDL3	0.141	0.390	0.363	Purifying	31.961	1.05 × 10^−17^
GDPDL1-GDPD-RLK	0.120	0.327	0.368	Purifying	26.786	5.38 × 10^−16^
GDPD-RLK-GDPDL1	0.120	0.327	0.368	Purifying	26.786	5.38 × 10^−16^
GDPDL1-GDPD (ectodomain)	0.125	0.326	0.384	Purifying	26.730	5.08 × 10^−15^

**Table 11:** Protein-protein interactions between the GDPDL proteins and Arabidopsis proteins. The colors indicate the hubs from Figure 3A.

Name	Betweenness Centrality	Closeness Centrality	Degree	Eccentricity	Description
SNC4	0.19234075	0.37614679	12	3	glycerophosphoryl diester phosphodiesterase family protein, putative, expressed
RLP51	0.0	0.27516779	2	4	leucine rich repeat family protein, putative, expressed
SNC1	3.0111 × 10^−4^	0.27702703	4	4	rp3 protein, putative, expressed
SUA	1.0037 × 10^−4^	0.27702703	4	4	RNA recognition motif family protein, expressed
DRT111	1.0037 × 10^−4^	0.27702703	4	4	G-patch domain containing protein, expressed
AT2G20050	0.0	0.27424749	1	4	AGC_PKA/PKG_like.1-ACG kinases include homologs to PKA, PKG and PKC, expressed
AT1G59780	0.0	0.27424749	1	4	NBS-LRR disease resistance protein, putative, expressed
AT3G55350	0.0	0.27609428	3	4	trp repressor/replication initiator, putative, expressed
BPA1	0.30818366	0.51898734	6	2	RNA recognition motif containing protein, putative, expressed
AT4G1772	0.30818366	0.51898734	6	2	RNA recognition motif, putative, expressed
AT1G22920	0.0	0.27424749	2	4	COP9 signalosome complex subunit 5b, putative, expressed
GDPDL5	0.17835276	0.37104072	10	3	glycerophosphoryl diester phosphodiesterase family protein, putative, expressed
MLP328	0.0	0.27702703	7	4	pathogenesis-related Bet v I family protein, putative, expressed
AGL46	0.0	0.27702703	7	4	OsMADS89-MADS-box family gene with M-gamma type-box, expressed
AT2G47115	0.04302	0.2779661	8	4	expressed protein
AT1G29660	0.04302	0.2779661	8	4	GDSL-like lipase/acylhydrolase, putative, expressed
AT5G51950	0.04302	0.2779661	8	4	HOTHEAD precursor, putative, expressed
AT1G20680	0.04302	0.2779661	8	4	Ser/Thr-rich protein T10 in DGCR region, putative, expressed
AT2G17710	0.04302	0.2779661	8	4	expressed protein
AT5G42530	0.04302	0.2779661	8	4	
BPA1	0.30818366	0.51898734	6	2	RNA recognition motif containing protein, putative, expressed
AT4G17720	0.30818366	0.51898734	6	2	RNA recognition motif, putative, expressed
GDPDL3	0.1693342	0.37104072	10	3	glycerophosphoryl diester phosphodiesterase family protein, putative, expressed
SHV2	0.0	0.27516779	5	4	COBRA-like protein 7 precursor, putative, expressed
MRH1	0.0	0.27516779	5	4	MRH1, putative, expressed
BST1	0.0	0.27516779	5	4	endonuclease/exonuclease/phosphatase family domain containing protein, expressed
MRH6	0.0	0.27516779	5	4	universal stress protein domain containing protein, putative, expressed
MRH2	0.0	0.27516779	5	4	kinesin motor domain containing protein, expressed
ATCOAE	0.0	0.27152318	1	4	dephospho-CoA kinase, putative, expressed
AT3G23750	0.0	0.27152318	1	4	receptor protein kinase TMK1 precursor, putative, expressed
BPA1	0.30818366	0.51898734	6	2	RNA recognition motif containing protein, putative, expressed
AT4G17720	0.30818366	0.51898734	6	2	RNA recognition motif, putative, expressed
GDPDL1	0.12794717	0.37442922	10	3	glycerophosphoryl diester phosphodiesterase family protein, putative, expressed
AT1G49750	0.0	0.27333333	1	4	uncharacterized protein At4g06744 precursor, putative, expressed
AT3G45710	0.0	0.27333333	1	4	peptide transporter PTR2, putative, expressed
PLDGAMMA1	0.00779455	0.29181495	3	4	phospholipase D, putative, expressed
MAP18	0.0	0.27333333	1	4	Unknown function
CDS1	0.0	0.28275862	2	4	phosphatidate cytidylyltransferase, putative, expressed
BPA1	0.30818366	0.51898734	6	2	RNA recognition motif containing protein, putative, expressed
AT4G17720	0.30818366	0.51898734	6	2	RNA recognition motif, putative, expressed
GDPDL4	0.21573054	0.38497653	14	3	glycerophosphoryl diester phosphodiesterase family protein, putative, expressed
AT5G38480	0.0	0.27891156	1	4	14-3-3 protein, putative, expressed
FLA7	0.00445805	0.29390681	6	4	fasciclin domain containing protein, expressed
SKU5	0.0	0.2877193	4	4	monocopper oxidase, putative, expressed
FLA8	0.0	0.2877193	4	4	fasciclin-like arabinogalactan protein, putative, expressed
ZW9	0.00445805	0.29390681	6	4	ubiquitin carboxyl-terminal hydrolase, putative, expressed
AT1G32860	0.00853443	0.29496403	2	4	glycosyl hydrolases family 17, putative, expressed
AT3G56370	0.0	0.27891156	1	4	receptor-like protein kinase precursor, putative, expressed
AT4G09000	0.0	0.27891156	1	4	14-3-3 protein, putative, expressed
BG_PPAP	0.0	0.27891156	1	4	glycosyl hydrolases family 17, putative, expressed
AT1G01080	0.06480132	0.39047619	3	4	RNA recognition motif containing protein, putative, expressed
AT5G65430	0.0	0.27891156	1	4	14-3-3 protein, putative, expressed
BPA1	0.30818366	0.51898734	6	2	RNA recognition motif containing protein, putative, expressed
AT4G17720	0.30818366	0.51898734	6	2	RNA recognition motif, putative, expressed
GDPDL6	0.67455299	0.4969697	38	3	glycerophosphoryl diester phosphodiesterase family protein, putative, expressed
AT4G11860	0.0	0.27891156	1	4	ubiquitin interaction motif family protein, expressed
AT3G23410	0.0	0.33333333	1	4	alcohol oxidase, putative, expressed
AT4G23400	0.0	0.33333333	1	4	aquaporin protein, putative, expressed
AT4G30850	0.0	0.33333333	1	4	haemolysin-III, putative, expressed
AT1G57870	0.0	0.33333333	1	4	CGMC_GSK.5-CGMC includes CDA, MAPK, GSK3, and CLKC kinases, expressed
AT1G31812	0.0	0.33333333	1	4	acyl CoA binding protein, putative, expressed
AT1G14360	0.0	0.33333333	1	4	solute carrier family 35 member B1, putative, expressed
AT5G06320	0.0	0.33333333	1	4	harpin-induced protein 1 domain containing protein, expressed
AT1G07550	0.0	0.33333333	1	4	senescence-induced receptor-like serine/threonine-protein kinase precursor, putative, expressed
AT5G07340	0.0	0.33333333	1	4	calreticulin precursor protein, putative, expressed
AT2G41705	0.0	0.33333333	1	4	crcB-like protein, expressed
AT3G12180	0.0	0.33333333	1	4	cornichon protein, putative, expressed
AT5G11890	0.0	0.33333333	1	4	harpin-induced protein 1 domain containing protein, expressed
AT1G14020	0.0	0.33333333	1	4	auxin-independent growth promoter protein, putative, expressed
AT1G34640	0.0	0.33333333	1	4	expressed protein
AT3G66654	0.0	0.33333333	1	4	peptidyl-prolyl cis-trans isomerase, putative, expressed
AT2G22425	0.0	0.33333333	1	4	signal peptidase complex subunit 1, putative, expressed
AT2G27290	0.0	0.33333333	1	4	protein of unknown function DUF1279 domain containing protein, expressed
AT5G49540	0.0	0.33333333	1	4	transmembrane protein 93, putative, expressed
AT1G13770	0.0	0.33333333	1	4	DUF647 domain containing protein, putative, expressed
AT1G29060	0.0	0.33333333	1	4	expressed protein
AT4G14455	0.0	0.33333333	1	4	SNARE domain containing protein, putative, expressed
AT4G25360	0.0	0.33333333	1	4	leaf senescence related protein, putative, expressed
AT4G12250	0.0	0.33333333	1	4	UDP-glucuronate 4-epimerase, putative, expressed
AT5G35460	0.0	0.33333333	1	4	integral membrane protein, putative, expressed
AT1G16170	0.0	0.33333333	1	4	expressed protein
AT5G03345	0.0	0.33333333	1	4	expressed protein
AT1G47640	0.0	0.33333333	1	4	SSA2-2S albumin seed storage family protein precursor, putative, expressed
AT5G52420	0.0	0.33333333	1	4	expressed protein
BPA1	0.30818366	0.51898734	6	2	RNA recognition motif containing protein, putative, expressed
AT4G17720	0.30818366	0.51898734	6	2	RNA recognition motif, putative, expressed

## Data Availability

The data are available at http://209.145.56.49:8080/web/.

## Supplementary Materials

The supporting information can be downloaded at: https://www.mdpi.com/article/10.3390/ijms232012176/s1.

## Abbreviations

ACC	accuracy	ML	machine learning
BAK1	BRI1-ASSOCIATED RECEPTOR KINASE1	MLPL	major latex protein-like
BRI1	BRASSINOSTEROID INSENSITIVE 1	MS	Murashige and Skoog
CabLCV	cabbage leaf curl virus	NEP1	NECROSIS- AND ETHYLENE-INDUCING PEPTIDE 1
CAP	adenylate-cyclase-associated	NLPs	NEP1-LIKE PROTEINS
CERK1	CHITIN ELICITOR RECEPTOR KINASE 1	NRLPs	non-RLPs
CLV1	CLAVATA1	nsLTP	non-specific lipid transfer proteins
CPAASC2	chemical properties of amino acid side chains 2	PAMPs	pathogen-associated molecular patterns
DAMPs	damage-associated molecular patterns	PEPR1	PEP1 RECEPTOR 1
ECD	extracellular domain	PEPR2	PEP1 RECEPTOR 2
EPF1	EPIDERMAL PATTERNING FACTOR 1	PPI	protein-protein interaction
EPF2	EPIDERMAL PATTERNING FACTOR 2	PRRs	pattern recognition receptors
ER	endoplasmic reticulum	PSK	PHYTOSULFOKINE
ERL1	ERECTA-LIKE 1	PSKR1	PHYTOSULFOKINE RECEPTOR 1
ETI	effector-triggered immunity	PPI	protein-protein interactions
FDR	false discovery rate	PTI	PAMP-triggered immunity
GDPDL	glycerophosphoryl diester phosphodiesterase family	RLCK	receptor-like cytoplasmic kinases
HMM	hidden Markov model	RLP	receptor-like protein
LRR	leucine-rich repeat	SOBIR1	SUPPRESSOR OF BIR1-1
LRR-RLK	leucine-rich repeat kinase receptor-like kinase	SP	signal peptide
LYM1	LYSIN-MOTIF 1	TMM	RLP TOO MANY MOUTHS
LYM3	LYSIN-MOTIF 3	TN	true negatives
LysM	lysin-motifs	TP	true positives
MCC	Mathew’s correlation coefficient	TRV	tobacco rattle virus
