# Supplementary material for: RLPredictiOme, a Machine Learning-Derived Method for High-Throughput Prediction of Plant Receptor-like Proteins, Reveals Novel Classes of Transmembrane Receptors
Source: Int J Mol Sci. 2022 Oct 12;23(20):12176. doi: 10.3390/ijms232012176 (PMC9603095; doi:10.3390/ijms232012176)
Supplement: Supplementary file 1 [file ijms-23-12176-s001.zip › Supplementary_Tables.pdf]

Supplementary Data

RLPredictiOme, a machine learning-derived method for high-throughput prediction of plant receptor-like proteins, reveals novel classes of transmembrane receptors

Table S1. Validation of the LRR-RLP subfamily of the rice genome.

SP	TM	RLP/NRLP	RLP/RLP Probability	RLP/RLK	RLP/RLK Probability	RLP-Subfamily	RLP-Subfamily Probability	Classification	Decision probability
**Y**	Y	RLP	0.9964	RLP	0.9909	LRR-RLP	0.8335	(LRR-RLP)	0.991
**Y**	Y	RLP	0.9966	RLP	0.9917	LRR-RLP	0.8514	(LRR-RLP)	0.9898
**Y**	Y	RLP	0.9966	RLP	0.9913	LRR-RLP	0.8507	(LRR-RLP)	0.9893
**Y**	Y	RLP	0.9958	RLP	0.7127	LRR-RLP	0.8501	(LRR-RLP)	0.9893
**Y**	Y	RLP	0.9961	RLP	0.8586	LRR-RLP	0.7664	(LRR-RLP)	0.9893
**Y**	Y	RLP	0.9968	RLP	0.8564	LRR-RLP	0.8506	(LRR-RLP)	0.9891
**Y**	Y	RLP	0.9962	RLP	0.9895	LRR-RLP	0.8501	(LRR-RLP)	0.9891
**Y**	Y	RLP	0.9967	RLP	0.8576	LRR-RLP	0.8493	(LRR-RLP)	0.9891
**Y**	Y	RLP	0.9965	RLP	0.8494	LRR-RLP	0.8331	(LRR-RLP)	0.989
**Y**	Y	RLP	0.9958	RLP	0.9896	LRR-RLP	0.8502	(LRR-RLP)	0.9889
**Y**	Y	RLP	0.9958	RLP	0.9913	LRR-RLP	0.8339	(LRR-RLP)	0.9889
**Y**	Y	RLP	0.9962	RLP	0.9897	LRR-RLP	0.8509	(LRR-RLP)	0.9888
**Y**	Y	RLP	0.9961	RLP	0.8568	LRR-RLP	0.7677	(LRR-RLP)	0.9888
**Y**	Y	RLP	0.9967	RLP	0.9899	LRR-RLP	0.8493	(LRR-RLP)	0.9888
**Y**	Y	RLP	0.9472	RLP	0.9907	LRR-RLP	0.8505	(LRR-RLP)	0.9888
**Y**	Y	RLP	0.9965	RLP	0.8566	LRR-RLP	0.8497	(LRR-RLP)	0.9887
**Y**	Y	RLP	0.9967	RLP	0.8593	LRR-RLP	0.8485	(LRR-RLP)	0.9886
**Y**	Y	RLP	0.9961	RLP	0.8544	LRR-RLP	0.833	(LRR-RLP)	0.9886
**Y**	Y	RLP	0.9967	RLP	0.9915	LRR-RLP	0.8501	(LRR-RLP)	0.9883
**Y**	Y	RLP	0.9963	RLP	0.8562	LRR-RLP	0.5677	(LRR-RLP)	0.9883
**Y**	Y	RLP	0.9964	RLP	0.991	LRR-RLP	0.8501	(LRR-RLP)	0.9882
**Y**	Y	RLP	0.9959	RLP	0.9901	LRR-RLP	0.8511	(LRR-RLP)	0.9881
**Y**	Y	RLP	0.9966	RLP	0.9889	LRR-RLP	0.8496	(LRR-RLP)	0.988
**Y**	Y	RLP	0.9968	RLP	0.8577	LRR-RLP	0.8494	(LRR-RLP)	0.988
**Y**	Y	RLP	0.9965	RLP	0.9918	LRR-RLP	0.8502	(LRR-RLP)	0.9878

**Y**	Y	RLP	0.9959	RLP	0.8523	LRR-RLP	0.8511	(LRR-RLP)	0.9878
**Y**	Y	RLP	0.9949	RLP	0.9899	LRR-RLP	0.8504	(LRR-RLP)	0.9878
**Y**	Y	RLP	0.9961	RLP	0.9917	LRR-RLP	0.85	(LRR-RLP)	0.9877
**Y**	Y	RLP	0.9961	RLP	0.9891	LRR-RLP	0.8	(LRR-RLP)	0.9877
**Y**	Y	RLP	0.9963	RLP	0.7165	LRR-RLP	0.7998	(LRR-RLP)	0.9877
**Y**	Y	RLP	0.9962	RLP	0.8512	LRR-RLP	0.8511	(LRR-RLP)	0.9876
**Y**	Y	RLP	0.9968	RLP	0.7135	LRR-RLP	0.8328	(LRR-RLP)	0.9876
**Y**	Y	RLP	0.9964	RLP	0.9892	LRR-RLP	0.8501	(LRR-RLP)	0.9876
**Y**	Y	RLP	0.9964	RLP	0.8568	LRR-RLP	0.8495	(LRR-RLP)	0.9876
**Y**	Y	RLP	0.996	RLP	0.9917	LRR-RLP	0.85	(LRR-RLP)	0.9876
**Y**	Y	RLP	0.9971	RLP	0.8592	LRR-RLP	0.7994	(LRR-RLP)	0.9875
**Y**	Y	RLP	0.996	RLP	0.8597	LRR-RLP	0.833	(LRR-RLP)	0.9875
**Y**	Y	RLP	0.9964	RLP	0.9903	LRR-RLP	0.8485	(LRR-RLP)	0.9875
**Y**	Y	RLP	0.9965	RLP	0.8578	LRR-RLP	0.8497	(LRR-RLP)	0.9874
**Y**	Y	RLP	0.9963	RLP	0.9905	LRR-RLP	0.8491	(LRR-RLP)	0.9873
**Y**	Y	RLP	0.9968	RLP	0.9909	LRR-RLP	0.8517	(LRR-RLP)	0.9872
**Y**	Y	RLP	0.9968	RLP	0.8554	LRR-RLP	0.8337	(LRR-RLP)	0.9871
**Y**	Y	RLP	0.9963	RLP	0.9902	LRR-RLP	0.6662	(LRR-RLP)	0.9871
**Y**	Y	RLP	0.9968	RLP	0.9905	LRR-RLP	0.7171	(LRR-RLP)	0.9871
**Y**	Y	RLP	0.9963	RLP	0.9907	LRR-RLP	0.6671	(LRR-RLP)	0.9871
**Y**	Y	RLP	0.8973	RLP	0.9916	LRR-RLP	0.4337	(LRR-RLP)	0.987
**Y**	Y	RLP	0.9962	RLP	0.9905	LRR-RLP	0.8497	(LRR-RLP)	0.9869
**Y**	Y	RLP	0.9963	RLP	0.9925	LRR-RLP	0.8496	(LRR-RLP)	0.9869
**Y**	Y	RLP	0.9959	RLP	0.7173	LRR-RLP	0.7996	(LRR-RLP)	0.9868
**Y**	Y	RLP	0.9962	RLP	0.9894	LRR-RLP	0.8491	(LRR-RLP)	0.9868
**Y**	Y	RLP	0.9965	RLP	0.9907	LRR-RLP	0.8502	(LRR-RLP)	0.9868
**Y**	Y	RLP	0.9967	RLP	0.8521	LRR-RLP	0.8332	(LRR-RLP)	0.9867
**Y**	Y	RLP	0.948	RLP	0.9917	LRR-RLP	0.8338	(LRR-RLP)	0.9867
**Y**	Y	RLP	0.9969	RLP	0.9909	LRR-RLP	0.8497	(LRR-RLP)	0.9866
**Y**	Y	RLP	0.9971	RLP	0.9912	LRR-RLP	0.6671	(LRR-RLP)	0.9866
**Y**	Y	RLP	0.9473	RLP	0.858	LRR-RLP	0.8508	(LRR-RLP)	0.9866
**Y**	Y	RLP	0.996	RLP	0.991	LRR-RLP	0.8334	(LRR-RLP)	0.9866
**Y**	Y	RLP	0.9971	RLP	0.8539	LRR-RLP	0.8496	(LRR-RLP)	0.9866
**Y**	Y	RLP	0.9971	RLP	0.9922	LRR-RLP	0.6664	(LRR-RLP)	0.9866
**Y**	Y	RLP	0.9962	RLP	0.9913	LRR-RLP	0.8168	(LRR-RLP)	0.9866
**Y**	Y	RLP	0.9967	RLP	0.858	LRR-RLP	0.8496	(LRR-RLP)	0.9865
**Y**	Y	RLP	0.9968	RLP	0.8505	LRR-RLP	0.833	(LRR-RLP)	0.9864
**Y**	Y	RLP	0.9963	RLP	0.9904	LRR-RLP	0.8504	(LRR-RLP)	0.9864

**Y**	Y	RLP	0.9965	RLP	0.8564	LRR-RLP	0.833	(LRR-RLP)	0.9863
**Y**	Y	RLP	0.8923	RLP	0.576	LRR-RLP	0.8166	(LRR-RLP)	0.9862
**Y**	Y	RLP	0.9958	RLP	0.9912	LRR-RLP	0.7004	(LRR-RLP)	0.9862
**Y**	Y	RLP	0.9968	RLP	0.9905	LRR-RLP	0.8503	(LRR-RLP)	0.9861
**Y**	Y	RLP	0.9961	RLP	0.8515	LRR-RLP	0.8163	(LRR-RLP)	0.9861
**Y**	Y	RLP	0.9966	RLP	0.8542	LRR-RLP	0.8504	(LRR-RLP)	0.9861
**Y**	Y	RLP	0.997	RLP	0.9916	LRR-RLP	0.8163	(LRR-RLP)	0.9861
**Y**	Y	RLP	0.9968	RLP	0.9902	LRR-RLP	0.8496	(LRR-RLP)	0.9859
**Y**	Y	RLP	0.9962	RLP	0.9906	LRR-RLP	0.8333	(LRR-RLP)	0.9859
**Y**	Y	RLP	0.9968	RLP	0.9897	LRR-RLP	0.8507	(LRR-RLP)	0.9857
**Y**	Y	RLP	0.9966	RLP	0.9903	LRR-RLP	0.6996	(LRR-RLP)	0.9856
**Y**	Y	RLP	0.9963	RLP	0.9912	LRR-RLP	0.8498	(LRR-RLP)	0.9855
**Y**	Y	RLP	0.9968	RLP	0.9908	LRR-RLP	0.85	(LRR-RLP)	0.9855
**Y**	Y	RLP	0.9963	RLP	0.9911	Unknown	0.3165	(Unknown)	0.9855
**Y**	Y	RLP	0.9965	RLP	0.9906	LRR-RLP	0.8009	(LRR-RLP)	0.9852
**Y**	Y	RLP	0.996	RLP	0.99	LRR-RLP	0.6337	(LRR-RLP)	0.9844
**Y**	Y	RLP	0.996	RLP	0.9911	LRR-RLP	0.8157	(LRR-RLP)	0.9825
**N**	Y	RLP	0.9964	RLP	0.9908	LRR-RLP	0.8497	(LRR-RLP)	0.8062
**N**	Y	RLP	0.9968	RLP	0.9906	LRR-RLP	0.6496	(LRR-RLP)	0.805
**N**	Y	RLP	0.9962	RLP	0.9908	LRR-RLP	0.8329	(LRR-RLP)	0.8049
**N**	Y	RLP	0.9957	RLP	0.9905	LRR-RLP	0.3511	(LRR-RLP)	0.8049
**N**	Y	RLP	0.9966	RLP	0.7123	LRR-RLP	0.8488	(LRR-RLP)	0.8048
**N**	Y	RLP	0.9955	RLP	0.9902	LRR-RLP	0.3835	(LRR-RLP)	0.8043
**N**	Y	RLP	0.9966	RLP	0.9894	LRR-RLP	0.8332	(LRR-RLP)	0.8041
**N**	Y	RLP	0.7891	RLP	0.7116	LRR-RLP	0.5184	(LRR-RLP)	0.804
**N**	Y	RLP	0.9964	RLP	0.7185	LRR-RLP	0.8498	(LRR-RLP)	0.8038
**N**	Y	RLP	0.9965	RLP	0.991	LRR-RLP	0.849	(LRR-RLP)	0.8033
**N**	Y	RLP	0.9963	RLP	0.9908	LRR-RLP	0.6168	(LRR-RLP)	0.8031
**N**	Y	RLP	0.9963	RLP	0.9918	LRR-RLP	0.634	(LRR-RLP)	0.8031
**N**	Y	RLP	0.9963	RLP	0.9917	LRR-RLP	0.8163	(LRR-RLP)	0.8028
**N**	Y	RLP	0.9963	RLP	0.8557	LRR-RLP	0.85	(LRR-RLP)	0.8021
**N**	Y	RLP	0.9961	RLP	0.9912	LRR-RLP	0.4171	(LRR-RLP)	0.8018
**N**	Y	RLP	0.997	RLP	0.9921	LRR-RLP	0.851	(LRR-RLP)	0.8014
**N**	Y	RLP	0.9966	RLP	0.9914	LRR-RLP	0.8497	(LRR-RLP)	0.8014
**N**	Y	RLP	0.9968	RLP	0.991	LRR-RLP	0.6508	(LRR-RLP)	0.8013
**N**	Y	RLP	0.9959	RLP	0.9903	LRR-RLP	0.8505	(LRR-RLP)	0.8012
**N**	Y	RLP	0.996	RLP	0.9912	LRR-RLP	0.851	(LRR-RLP)	0.8011
**N**	Y	RLP	0.947	RLP	0.9906	LRR-RLP	0.6663	(LRR-RLP)	0.8011

**N**	Y	RLP	0.9961	RLP	0.7127	LRR-RLP	0.8489	(LRR-RLP)	0.8009
**N**	Y	RLP	0.9962	RLP	0.9892	LRR-RLP	0.8501	(LRR-RLP)	0.8007
**N**	Y	RLP	0.9968	RLP	0.8573	LRR-RLP	0.8164	(LRR-RLP)	0.8006
**N**	Y	RLP	0.9959	RLP	0.9913	LRR-RLP	0.8329	(LRR-RLP)	0.8004
**N**	Y	RLP	0.9966	RLP	0.9909	LRR-RLP	0.8494	(LRR-RLP)	0.8004
**N**	Y	RLP	0.9963	RLP	0.991	LRR-RLP	0.6495	(LRR-RLP)	0.8002
**N**	Y	RLP	0.9966	RLP	0.855	LRR-RLP	0.85	(LRR-RLP)	0.8001
**N**	Y	RLP	0.9968	RLP	0.9914	LRR-RLP	0.6665	(LRR-RLP)	0.8
**N**	Y	RLP	0.9961	RLP	0.8561	LRR-RLP	0.8491	(LRR-RLP)	0.8
**N**	Y	RLP	0.9959	RLP	0.7158	LRR-RLP	0.8509	(LRR-RLP)	0.7999
**N**	Y	RLP	0.9958	RLP	0.9901	LRR-RLP	0.5669	(LRR-RLP)	0.7999
**N**	Y	RLP	0.9967	RLP	0.9901	LRR-RLP	0.8489	(LRR-RLP)	0.7999
**N**	Y	RLP	0.9967	RLP	0.9912	LRR-RLP	0.6007	(LRR-RLP)	0.7991
**N**	Y	RLP	0.9962	RLP	0.9909	LRR-RLP	0.8512	(LRR-RLP)	0.7986
**N**	Y	RLP	0.9967	RLP	0.9893	LRR-RLP	0.8332	(LRR-RLP)	0.7986
**N**	Y	RLP	0.9473	RLP	0.9905	LRR-RLP	0.6664	(LRR-RLP)	0.7986
**N**	Y	RLP	0.9958	RLP	0.9911	LRR-RLP	0.8173	(LRR-RLP)	0.7985
**N**	Y	RLP	0.9963	RLP	0.9904	LRR-RLP	0.5822	(LRR-RLP)	0.7985
**N**	Y	RLP	0.9967	RLP	0.9915	LRR-RLP	0.6675	(LRR-RLP)	0.7984
**N**	Y	RLP	0.9963	RLP	0.8591	LRR-RLP	0.8174	(LRR-RLP)	0.7984
**N**	Y	RLP	0.9968	RLP	0.9908	LRR-RLP	0.6674	(LRR-RLP)	0.7983
**N**	Y	RLP	0.9964	RLP	0.9928	LRR-RLP	0.8505	(LRR-RLP)	0.7982
**N**	Y	RLP	0.9968	RLP	0.7155	LRR-RLP	0.8331	(LRR-RLP)	0.7981
**N**	Y	RLP	0.9457	RLP	0.9907	LRR-RLP	0.8507	(LRR-RLP)	0.798
**N**	Y	RLP	0.9968	RLP	0.9907	LRR-RLP	0.7002	(LRR-RLP)	0.798
**N**	Y	RLP	0.9968	RLP	0.8562	LRR-RLP	0.8509	(LRR-RLP)	0.7978
**N**	Y	RLP	0.9968	RLP	0.991	LRR-RLP	0.8329	(LRR-RLP)	0.7975
**N**	Y	RLP	0.9961	RLP	0.9909	LRR-RLP	0.4158	(LRR-RLP)	0.7975
**N**	Y	RLP	0.996	RLP	0.9898	LRR-RLP	0.8319	(LRR-RLP)	0.7974
**N**	Y	RLP	0.6299	RLP	0.9904	Other-RLP	0.3164	(Other-RLP)	0.7971
**N**	Y	RLP	0.9967	RLP	0.9909	LRR-RLP	0.8168	(LRR-RLP)	0.7971
**N**	Y	RLP	0.9961	RLP	0.8608	LRR-RLP	0.7661	(LRR-RLP)	0.7971
**N**	Y	RLP	0.9962	RLP	0.9919	LRR-RLP	0.8493	(LRR-RLP)	0.797
**N**	Y	RLP	0.7911	RLP	0.99	Other-RLP	0.3841	(Other-RLP)	0.797
**N**	Y	RLP	0.996	RLP	0.9916	LRR-RLP	0.6673	(LRR-RLP)	0.797
**N**	Y	RLP	0.9966	RLP	0.9915	LRR-RLP	0.6666	(LRR-RLP)	0.7969
**N**	Y	RLP	0.6834	RLP	0.7095	LRR-RLP	0.8495	(LRR-RLP)	0.7969
**N**	Y	RLP	0.996	RLP	0.9911	LRR-RLP	0.6671	(LRR-RLP)	0.7968

**N**	Y	RLP	0.996	RLP	0.9919	LRR-RLP	0.8508	(LRR-RLP)	0.7967
**N**	Y	RLP	0.9956	RLP	0.9901	LRR-RLP	0.7003	(LRR-RLP)	0.7966
**N**	Y	RLP	0.9964	RLP	0.9901	LRR-RLP	0.8497	(LRR-RLP)	0.7959
**N**	Y	RLP	0.9965	RLP	0.9887	LRR-RLP	0.8488	(LRR-RLP)	0.7958
**N**	Y	RLP	0.9958	RLP	0.9908	LRR-RLP	0.7502	(LRR-RLP)	0.7957
**N**	Y	RLP	0.9961	RLP	0.9911	LRR-RLP	0.6836	(LRR-RLP)	0.7951
**N**	Y	RLP	0.9969	RLP	0.99	LRR-RLP	0.8001	(LRR-RLP)	0.795
**N**	Y	RLP	0.9963	RLP	0.9896	LRR-RLP	0.8499	(LRR-RLP)	0.7947
**N**	Y	RLP	0.9962	RLP	0.9922	LRR-RLP	0.8168	(LRR-RLP)	0.7939
**N**	Y	RLP	0.9962	RLP	0.9909	LRR-RLP	0.6679	(LRR-RLP)	0.7938
**N**	Y	RLP	0.9964	RLP	0.9919	LRR-RLP	0.8502	(LRR-RLP)	0.7936
**N**	Y	RLP	0.9959	RLP	0.991	LRR-RLP	0.6161	(LRR-RLP)	0.7935
**N**	Y	RLP	0.9969	RLP	0.9906	LRR-RLP	0.6662	(LRR-RLP)	0.7934
**N**	Y	RLP	0.996	RLP	0.9898	LRR-RLP	0.8497	(LRR-RLP)	0.7922
**N**	Y	RLP	0.9961	RLP	0.9901	LRR-RLP	0.8505	(LRR-RLP)	0.792
**Y**	N	RLP	0.9499	RLP	0.8572	LRR-RLP	0.7006	noRLP	0.2033
**N**	N	RLP	0.9468	RLP	0.8516	Unknown	0.2667	noRLP	0.2008
**Y**	N	RLP	0.9468	RLP	0.8537	LRR-RLP	0.5839	noRLP	0.1974
**N**	N	noRLP	0.1575	RLP	0.5637	Ethylene-responsive-RLP	0.4992	noRLP	0.0012

Table S2. High throughput prediction of the Arabidopsis proteins by RLPrefictiOme. The color codes match with Table 9.

ACCESSION	SP	TM	RLP X NRLP	RLP/ NRLP PROBABILITY	RLP X RLK	RLP/ RLK PROBABILITY	RLP-SUBFAMILY	RLP-SUBFAMILY PROBABILITY	CLASSIFICATION	DECISION PROBABILITY	DESCRIPTION
AT1G65380.1	Y	Y	RLP	0.9962	RLP	0.9907	LRR-RLP	0.8505	(LRR-RLP)	0.9902	**AtRLP10**
AT1G17240.1	Y	Y	RLP	0.9962	RLP	0.9913	LRR-RLP	0.8497	(LRR-RLP)	0.9886	**AtRLP2**
AT4G18760.1	Y	Y	RLP	0.9967	RLP	0.9903	LRR-RLP	0.8495	(LRR-RLP)	0.9885	**AtRLP51**
AT4G13880.1	Y	Y	RLP	0.9963	RLP	0.9899	LRR-RLP	0.8001	(LRR-RLP)	0.9884	**AtRLP48**
AT5G27060.1	Y	Y	RLP	0.9962	RLP	0.991	LRR-RLP	0.6669	(LRR-RLP)	0.9884	**AtRLP53**
AT3G23110.1	Y	Y	RLP	0.9964	RLP	0.9912	LRR-RLP	0.6502	(LRR-RLP)	0.9883	**AtRLP37**

AT1G80080.1	Y	Y	RLP	0.9961	RLP	0.9911	LRR-RLP	0.5506	(LRR-RLP)	0.9883	**AtRLP17**
AT2G32680.1	Y	Y	RLP	0.9967	RLP	0.9918	LRR-RLP	0.7838	(LRR-RLP)	0.9882	**AtRLP23**
AT3G11080.1	Y	Y	RLP	0.9962	RLP	0.991	LRR-RLP	0.8496	(LRR-RLP)	0.988	**AtRLP35**
AT1G74180.1	Y	Y	RLP	0.9959	RLP	0.858	LRR-RLP	0.8163	(LRR-RLP)	0.988	**AtRLP14**
AT3G05370.1	Y	Y	RLP	0.9962	RLP	0.8556	LRR-RLP	0.6337	(LRR-RLP)	0.988	**AtRLP31**
AT3G28890.1	Y	Y	RLP	0.9966	RLP	0.8561	LRR-RLP	0.6336	(LRR-RLP)	0.988	**AtRLP43**
AT5G45770.1	Y	Y	RLP	0.9965	RLP	0.99	LRR-RLP	0.683	(LRR-RLP)	0.9878	**AtRLP55**
AT2G25440.1	Y	Y	RLP	0.9962	RLP	0.9902	LRR-RLP	0.4832	(LRR-RLP)	0.9878	**AtRLP20**
AT5G65830.1	Y	Y	RLP	0.9966	RLP	0.8566	LRR-RLP	0.667	(LRR-RLP)	0.9876	**ATRLP57**
AT3G05360.1	Y	Y	RLP	0.9967	RLP	0.9913	LRR-RLP	0.6668	(LRR-RLP)	0.9876	**AtRLP30**
AT2G42800.1	Y	Y	RLP	0.9963	RLP	0.9908	LRR-RLP	0.6665	(LRR-RLP)	0.9876	**AtRLP29**
AT5G37360.1	Y	Y	RLP	0.6322	RLP	0.9916	LRR-RLP	0.4	(LRR-RLP)	0.9875	Subfamily not named and unknown function
AT2G33020.1	Y	Y	RLP	0.9966	RLP	0.9905	LRR-RLP	0.8161	(LRR-RLP)	0.9873	**AtRLP24**
AT1G74190.1	Y	Y	RLP	0.9959	RLP	0.8564	LRR-RLP	0.8499	(LRR-RLP)	0.9871	**AtRLP15**
AT2G15080.1	Y	Y	RLP	0.9965	RLP	0.9904	LRR-RLP	0.8502	(LRR-RLP)	0.987	**AtRLP19**
AT5G19230.1	Y	Y	RLP	0.9969	RLP	0.9904	LRR-RLP	0.4334	(LRR-RLP)	0.987	Subfamily not named and unknown function
AT1G45616.1	Y	Y	RLP	0.9961	RLP	0.9913	LRR-RLP	0.7665	(LRR-RLP)	0.9868	**AtRLP6**
AT3G05650.1	Y	Y	RLP	0.9964	RLP	0.9906	LRR-RLP	0.6664	(LRR-RLP)	0.9868	**AtRLP32**
AT3G05660.1	Y	Y	RLP	0.9966	RLP	0.8557	LRR-RLP	0.85	(LRR-RLP)	0.9866	**AtRLP33**
AT1G58190.1	Y	Y	RLP	0.9962	RLP	0.8521	LRR-RLP	0.6663	(LRR-RLP)	0.9866	**AtRLP9**
AT4G13920.1	Y	Y	RLP	0.9967	RLP	0.9911	LRR-RLP	0.8498	(LRR-RLP)	0.9865	**AtRLP50**
AT3G49750.1	Y	Y	RLP	0.9963	RLP	0.9909	LRR-RLP	0.7502	(LRR-RLP)	0.9865	**AtRLP44**
AT5G25910.1	Y	Y	RLP	0.9964	RLP	0.9899	LRR-RLP	0.8501	(LRR-RLP)	0.9864	**AtRLP52**
AT4G04220.1	Y	Y	RLP	0.9962	RLP	0.9911	LRR-RLP	0.8506	(LRR-RLP)	0.9863	**AtRLP46**
AT2G33060.1	Y	Y	RLP	0.9966	RLP	0.9914	LRR-RLP	0.8332	(LRR-RLP)	0.9863	**AtRLP27**

AT2G33050.1	Y	Y	RLP	0.9964	RLP	0.9915	LRR-RLP	0.7498	(LRR-RLP)	0.986	**AtRLP26**
AT4G28560.1	Y	Y	RLP	0.9965	RLP	0.9908	LRR-RLP	0.6164	(LRR-RLP)	0.986	**RIC7**
AT1G71400.1	Y	Y	RLP	0.996	RLP	0.8563	LRR-RLP	0.6831	(LRR-RLP)	0.9851	**AtRLP12**
AT1G71390.1	N	Y	RLP	0.9966	RLP	0.99	LRR-RLP	0.6667	(LRR-RLP)	0.8021	**AtRLP11**
AT2G25470.1	N	Y	RLP	0.9964	RLP	0.8556	LRR-RLP	0.8502	(LRR-RLP)	0.8014	**AtRLP21**
AT4G13810.1	N	Y	RLP	0.9964	RLP	0.9907	LRR-RLP	0.833	(LRR-RLP)	0.7997	**AtRLP47**
AT3G23010.1	N	Y	RLP	0.9965	RLP	0.9908	LRR-RLP	0.667	(LRR-RLP)	0.7995	**AtRLP36**
AT3G24982.1	N	Y	RLP	0.9963	RLP	0.989	LRR-RLP	0.8512	(LRR-RLP)	0.7993	**AtRLP40**
AT4G25750.1	N	Y	RLP	0.7363	RLP	0.9922	LRR-RLP	0.4168	(LRR-RLP)	0.7992	ABC-2 type transporter domain containing protein. expressed
AT1G17250.1	N	Y	RLP	0.9965	RLP	0.9911	LRR-RLP	0.8496	(LRR-RLP)	0.799	**AtRLP3**
AT3G21580.1	N	Y	RLP	0.8424	RLP	0.9903	LRR-RLP	0.3832	(LRR-RLP)	0.7981	cobalt ion transporter. putative. expressed
AT3G23120.1	N	Y	RLP	0.997	RLP	0.9905	LRR-RLP	0.6835	(LRR-RLP)	0.7976	**AtRLP38**
AT3G53240.1	N	Y	RLP	0.9961	RLP	0.9905	LRR-RLP	0.783	(LRR-RLP)	0.7973	**AtRLP45**
AT1G07390.1	N	Y	RLP	0.9957	RLP	0.7119	LRR-RLP	0.7826	(LRR-RLP)	0.7969	**AtRLP1**
AT3G11010.1	N	Y	RLP	0.9961	RLP	0.9902	LRR-RLP	0.6665	(LRR-RLP)	0.7958	**AtRLP34**
AT3G44070.1	N	Y	RLP	0.9961	RLP	0.9899	LRR-RLP	0.5335	(LRR-RLP)	0.7951	Subfamily not named and unknown function
AT5G49290.1	N	Y	RLP	0.9966	RLP	0.9901	LRR-RLP	0.6833	(LRR-RLP)	0.7941	**ATRLP56**
AT1G34290.1	Y	Y	RLP	0.9964	RLP	0.9898	**(Undefined)**	0.2166	**(Undefined)**	0.7949	**AtRLP5**
AT1G74170.1	N	Y	RLP	0.9964	RLP	0.8561	LRR-RLP	0.7164	(LRR-RLP)	0.7994	**AtRLP13**
AT1G47890.1	N	Y	RLP	0.9967	RLP	0.9908	LRR-RLP	0.8501	(LRR-RLP)	0.8001	**AtRLP7**
AT2G20520.1	Y	Y	RLP	0.8429	RLP	0.9908	LysM-RLP	0.6839	(LysM-RLP)	0.9885	fasciclin domain containing protein. expressed
AT2G48130.1	Y	Y	RLP	0.996	RLP	0.991	LysM-RLP	0.3337	(LysM-RLP)	0.9883	LTPL78 - Protease

											inhibitor / Other-RLP associated with Probable-lipid-transfer-RLK
AT2G27130.1	Y	Y	RLP	0.8431	RLP	0.9899	LysM-RLP	0.3001	(LysM-RLP)	0.9881	LTPL78 - Protease inhibitor / Other-RLP associated with Probable-lipid-transfer-RLK
AT5G64080.1	Y	Y	RLP	0.7392	RLP	0.9901	LysM-RLP	0.4837	(LysM-RLP)	0.988	LTPL78 - Protease inhibitor / Other-RLP associated with Probable-lipid-transfer-RLK
AT2G04780.1	Y	Y	RLP	0.8962	RLP	0.99	LysM-RLP	0.3665	(LysM-RLP)	0.9878	fasciclin domain containing protein. expressed
AT1G77630.1	Y	Y	RLP	0.9457	RLP	0.9912	LysM-RLP	0.5002	(LysM-RLP)	0.9876	**LysM-RLP**
AT4G14815.1	Y	Y	RLP	0.9475	RLP	0.9905	LysM-RLP	0.3501	(LysM-RLP)	0.9876	LTPL76 - Protease inhibitor / Other-RLP (Probable-lipid-transfer-RLK)
AT2G45470.1	Y	Y	RLP	0.9466	RLP	0.9911	LysM-RLP	0.5328	(LysM-RLP)	0.9875	fasciclin-like arabinogalactan protein. putative. expressed
AT2G32300.1	Y	Y	RLP	0.7903	RLP	0.9904	LysM-RLP	0.3502	(LysM-RLP)	0.9875	plastocyanin-like domain containing protein / Other-RLP associated with Plastocyanin-like-RLK
AT3G46550.1	Y	Y	RLP	0.9961	RLP	0.9896	LysM-RLP	0.3003	(LysM-RLP)	0.9874	fasciclin-like arabinogalactan protein. putative. expressed
AT3G06360.1	Y	Y	RLP	0.9963	RLP	0.9911	LysM-RLP	0.4834	(LysM-RLP)	0.9873	Subfamily not named and unknown function
AT4G14805.1	Y	Y	RLP	0.9962	RLP	0.9908	LysM-RLP	0.5666	(LysM-RLP)	0.9871	LTPL47 - Protease inhibitor / Other-RLP (Probable-lipid-

											transfer-RLK)
AT1G63550.1	Y	Y	RLP	0.9962	RLP	0.9906	LysM-RLP	0.4004	(LysM-RLP)	0.987	Salt stress response/antifungal
AT2G44300.1	Y	Y	RLP	0.7878	RLP	0.9888	LysM-RLP	0.3835	(LysM-RLP)	0.9868	LTPL82 - Protease inhibitor / Other-RLP associated with Probable-lipid-transfer-RLK
AT4G22666.1	Y	Y	RLP	0.7913	RLP	0.9917	LysM-RLP	0.3499	(LysM-RLP)	0.9867	LTPL85 - Protease inhibitor / Other-RLP associated with Probable-lipid-transfer-RLK
AT4G12360.1	Y	Y	RLP	0.996	RLP	0.9905	LysM-RLP	0.5332	(LysM-RLP)	0.9865	LTPL85 - Protease inhibitor / Other-RLP associated with Probable-lipid-transfer-RLK
AT1G73890.1	Y	Y	RLP	0.9479	RLP	0.9913	LysM-RLP	0.5662	(LysM-RLP)	0.9862	LTPL82 - Protease inhibitor / Other-RLP associated with Probable-lipid-transfer-RLK
AT1G73550.1	Y	Y	RLP	0.946	RLP	0.9903	Unknown	0.2503	(LysM-RLP)	0.7999	LTPL85 - Protease inhibitor / Other-RLP associated with Probable-lipid-transfer-RLK
AT5G26270.1	N	Y	RLP	0.7363	RLP	0.991	LysM-RLP	0.4834	(LysM-RLP)	0.7991	Subfamily not named and unknown function
AT2G17120.1	Y	Y	RLP	0.9959	RLP	0.9904	Unknown	0.2992	(LysM-RLP)	0.8015	**LysM-RLP**
AT2G19440.1	Y	Y	RLP	0.6853	RLP	0.7135	L-Lectin-RLP	0.3661	(L-Lectin-RLP)	0.9882	Glycosyl hydrolases family 17 (Glycosyl-hydrolases-RLP)
AT1G16022.1	Y	Y	RLP	0.8955	RLP	0.9902	L-Lectin-RLP	0.3341	(L-Lectin-RLP)	0.9875	Subfamily not named and unknown function

AT3G26110.1	Y	Y	RLP	0.8426	RLP	0.9901	L-Lectin-RLP	0.3501	(L-Lectin-RLP)	0.9872	Subfamily not named and unknown function
AT1G70170.1	Y	Y	RLP	0.9961	RLP	0.9912	L-Lectin-RLP	0.4999	(L-Lectin-RLP)	0.987	metalloendoproteinase 1 precursor. putative. expressed
AT4G31840.1	Y	Y	RLP	0.7364	RLP	0.9909	L-Lectin-RLP	0.3835	(L-Lectin-RLP)	0.9867	plastocyanin-like domain containing protein / Other-RLP associated with Plastocyanin-like-RLK
AT5G40620.1	Y	Y	RLP	0.9967	RLP	0.9901	L-Lectin-RLP	0.4836	(L-Lectin-RLP)	0.9866	Subfamily not named and unknown function
AT1G05140.1	N	Y	RLP	0.6822	RLP	0.7146	L-Lectin-RLP	0.3668	(L-Lectin-RLP)	0.8001	peptidase M50 family protein. putative. expressed
ATMG00410.1	N	Y	RLP	0.6299	RLP	0.9898	L-Lectin-RLP	0.3998	(L-Lectin-RLP)	0.7992	ATP synthase. A subunit family protein. putative. expressed
AT2G07741.1	N	Y	RLP	0.6325	RLP	0.9911	L-Lectin-RLP	0.3999	(L-Lectin-RLP)	0.7989	ATP synthase. A subunit family protein. putative. expressed
AT2G32480.1	N	Y	RLP	0.6305	RLP	0.9901	L-Lectin-RLP	0.4333	(L-Lectin-RLP)	0.7988	peptidase M50 family protein. putative. expressed
AT2G24945.1	N	Y	RLP	0.6837	RLP	0.9909	L-Lectin-RLP	0.3664	(L-Lectin-RLP)	0.7982	Subfamily not named and unknown function
AT5G37480.1	N	Y	RLP	0.7367	RLP	0.9911	L-Lectin-RLP	0.3835	(L-Lectin-RLP)	0.7933	Subfamily not named and unknown function
AT1G25570.1	Y	Y	RLP	0.9966	RLP	0.9902	Malectin-RLP	0.4674	(Malectin-RLP)	0.9884	Subfamily not named and unknown function
AT4G16120.1	Y	Y	RLP	0.9966	RLP	0.9915	Malectin-RLP	0.3004	(Malectin-RLP)	0.988	COBRA-like protein 7 precursor. putative. expressed
AT4G12420.1	Y	Y	RLP	0.6821	RLP	0.9895	Malectin-RLP	0.4832	(Malectin-RLP)	0.9877	monocopper oxidase. putative. expressed
AT1G28340.1	Y	Y	RLP	0.8425	RLP	0.9905	Malectin-RLP	0.4502	(Malectin-RLP)	0.9875	**Malectin-RLP**

AT3G46270.1	Y	Y	RLP	0.9964	RLP	0.9892	Malectin-RLP	0.5168	(Malectin-RLP)	0.9873	Subfamily not named and unknown function
AT4G18340.1	Y	Y	RLP	0.7893	RLP	0.9907	Malectin-RLP	0.333	(Malectin-RLP)	0.987	glycosyl hydrolases family 17. putative. expressed (Glycosyl-hydrolases-RLP)
AT4G25240.1	Y	Y	RLP	0.7897	RLP	0.9903	Malectin-RLP	0.3995	(Malectin-RLP)	0.9867	monocopper oxidase. putative. expressed
AT5G51480.1	Y	Y	RLP	0.9963	RLP	0.9902	Malectin-RLP	0.4167	(Malectin-RLP)	0.9866	monocopper oxidase. putative. expressed
AT3G46280.1	Y	Y	RLP	0.9966	RLP	0.9911	Malectin-RLP	0.4335	(Malectin-RLP)	0.9853	Subfamily not named and unknown function
AT5G49150.1	N	Y	RLP	0.8938	RLP	0.9914	Malectin-RLP	0.3336	(Malectin-RLP)	0.7986	GEX2. putative. expressed
AT2G04060.1	N	Y	RLP	0.6831	RLP	0.9898	Malectin-RLP	0.4003	(Malectin-RLP)	0.7933	beta-galactosidase precursor. putative. expressed (Glycosyl-hydrolases-RLP)
AT1G24485.1	Y	Y	RLP	0.9963	RLP	0.9914	Unknown	0.2172	(Malectin-RLP)	0.7941	**Malectin-RLP**
AT1G10380.1	Y	Y	RLP	0.9964	RLP	0.9897	Thaumatin-RLP	0.4165	(Thaumatin-RLP)	0.988	**Thaumatin-RLP**
AT4G38660.1	Y	Y	RLP	0.8941	RLP	0.9906	Thaumatin-RLP	0.634	(Thaumatin-RLP)	0.9876	**Thaumatin-RLP**
AT4G36010.1	Y	Y	RLP	0.84	RLP	0.9902	Thaumatin-RLP	0.7497	(Thaumatin-RLP)	0.9875	**Thaumatin-RLP**
AT1G75800.1	Y	Y	RLP	0.9964	RLP	0.9893	Thaumatin-RLP	0.8491	(Thaumatin-RLP)	0.9871	**Thaumatin-RLP**
AT1G04520.1	Y	Y	RLP	0.8433	RLP	0.9905	Thaumatin-RLP	0.3664	(Thaumatin-RLP)	0.987	**Thaumatin-RLP**
AT1G77700.1	N	Y	RLP	0.9966	RLP	0.9904	Thaumatin-RLP	0.5666	(Thaumatin-RLP)	0.7974	**Thaumatin-RLP**
AT2G20700.1	Y	Y	RLP	0.9967	RLP	0.9899	Salt-stress-response/ antifungal-RLP	0.3336	(Salt-stress-response/ antifungal-RLP)	0.9856	Subfamily not named and unknown function
AT3G60720.1	Y	Y	RLP	0.997	RLP	0.9908	Unknown	0.1832	(Salt-stress-response/ antifungal-RLP)	0.7982	**Salt stress response/antifungal-RLP**

AT2G33330.1	Y	Y	RLP	0.7908	RLP	0.9898	Unknown	0.2498	(Salt-stress-response/ antifungal-RLP)	0.7989	**Salt stress response/antifungal-RLP**
AT1G61750.1	Y	Y	RLP	0.9963	RLP	0.9901	Salt-stress-response/ antifungal-RLP	0.3334	(Salt-stress-response/ antifungal-RLP)	0.9862	**Salt stress response/antifungal-RLP**
AT5G53110.1	Y	Y	RLP	0.9957	RLP	0.9907	WAK-RLP	0.3333	(WAK-RLP)	0.989	**WAK-RLP**
AT4G14746.1	Y	Y	RLP	0.8375	RLP	0.991	WAK-RLP	0.3176	(WAK-RLP)	0.9885	Subfamily not named and unknown function
AT2G30290.1	Y	Y	RLP	0.6813	RLP	0.9904	WAK-RLP	0.3164	(WAK-RLP)	0.9879	vacuolar-sorting receptor precursor. putative. expressed
AT1G11915.1	Y	Y	RLP	0.9966	RLP	0.9913	WAK-RLP	0.5163	(WAK-RLP)	0.9877	**WAK-RLP**
AT2G46494.1	Y	Y	RLP	0.9964	RLP	0.9908	WAK-RLP	0.3334	(WAK-RLP)	0.9874	zinc finger. C3HC4 type domain containing protein. expressed
AT5G37660.1	Y	Y	RLP	0.9963	RLP	0.9895	WAK-RLP	0.317	(WAK-RLP)	0.987	Salt stress response/antifungal-RLP
AT1G02300.1	Y	Y	RLP	0.6844	RLP	0.8582	WAK-RLP	0.2334	(WAK-RLP)	0.8005	Papain family cysteine protease domain containing protein. expressed
AT1G66940.1	Y	Y	RLP	0.9962	RLP	0.9898	WAK-RLP	0.2665	(WAK-RLP)	0.7981	**WAK-RLP**
AT1G74045.1	N	Y	RLP	0.8418	RLP	0.9915	WAK-RLP	0.4006	(WAK-RLP)	0.7964	tetraspanin family protein. putative. expressed
AT1G70690.1	Y	Y	RLP	0.9964	RLP	0.9906	Unknown	0.2993	(WAK-RLP)	0.7949	Salt stress response/antifungal-RLP
AT5G43980.1	Y	Y	RLP	0.9965	RLP	0.9902	Unknown	0.2672	(WAK-RLP)	0.7911	Salt stress response/antifungal-RLP
AT2G46495.1	Y	Y	RLP	0.8945	RLP	0.9908	WAK-RLP	0.3003	(WAK-RLP)	0.9859	**WAK-RLP**

AT2G46495.1	Y	Y	RLP	0.8945	RLP	0.9908	Unknown	0.3003	(WAK-RLP)	0.9859	**WAK-RLP**
AT5G03700.1	Y	Y	RLP	0.7348	RLP	0.8555	S-domain-RLP	0.4498	(S-domain-RLP)	0.9878	**(S-domain-RLP)**
AT1G46840.1	N	Y	RLP	0.6293	RLP	0.9913	S-domain-RLP	0.4168	(S-domain-RLP)	0.7988	OsFBO21 - F-box and other domain containing protein. expressed
AT1G48940.1	Y	Y	RLP	0.8424	RLP	0.9902	Other-RLP	0.5169	(Other-RLP)	0.9888	**plastocyanin-like domain containing protein. putative. expressed (Plastocyanin-like-RLK)**
AT1G69980.1	Y	Y	RLP	0.684	RLP	0.992	Other-RLP	0.6165	(Other-RLP)	0.9884	Subfamily not named and unknown function
AT5G14030.1	Y	Y	RLP	0.6827	RLP	0.9902	Other-RLP	0.5163	(Other-RLP)	0.9883	translocon-associated protein beta domain containing protein. expressed
AT3G51580.1	Y	Y	RLP	0.7894	RLP	0.9908	Other-RLP	0.3504	(Other-RLP)	0.9882	Subfamily not named and unknown function
AT5G66160.1	Y	Y	RLP	0.6327	RLP	0.9913	Other-RLP	0.3168	(Other-RLP)	0.9877	**RING finger protein 13 / Other-RLK (Ring finger-RLK)**
AT3G51710.1	Y	Y	RLP	0.9453	RLP	0.9913	Other-RLP	0.4495	(Other-RLP)	0.9876	D-mannose binding lectin protein with Apple-like carbohydrate-binding domain (B_Lectin-RLP)
AT2G44290.1	Y	Y	RLP	0.7367	RLP	0.9896	Other-RLP	0.3168	(Other-RLP)	0.9875	**LTPL82 - Protease inhibitor / Other-RLP associated with Probable-lipid-transfer-RLK**

AT1G35630.1	Y	Y	RLP	0.6857	RLP	0.9908	Other-RLP	0.4832	(Other-RLP)	0.9874	**RING finger protein 13. putative. expressed (Ring finger-RLK)**
AT5G66820.1	Y	Y	RLP	0.7932	RLP	0.9904	Other-RLP	0.4829	(Other-RLP)	0.9874	Subfamily not named and unknown function
AT3G53490.1	Y	Y	RLP	0.63	RLP	0.991	Other-RLP	0.3332	(Other-RLP)	0.9874	Subfamily not named and unknown function
AT4G26690.1	Y	Y	RLP	0.9479	RLP	0.9896	Other-RLP	0.3832	(Other-RLP)	0.9873	**glycerophosphoryl diester phosphodiesterase family protein. putative. expressed**
AT5G16660.1	Y	Y	RLP	0.634	RLP	0.9915	Other-RLP	0.3831	(Other-RLP)	0.9872	Subfamily not named and unknown function
AT3G20520.1	Y	Y	RLP	0.8425	RLP	0.9913	Other-RLP	0.351	(Other-RLP)	0.9872	**glycerophosphoryl diester phosphodiesterase family protein. putative. expressed**
AT2G26600.1	Y	Y	RLP	0.6849	RLP	0.7123	Other-RLP	0.3501	(Other-RLP)	0.9872	**glycosyl hydrolases family 17. putative. expressed (Glycosyl-hydrolases-RLP)**
AT2G32670.1	Y	Y	RLP	0.7367	RLP	0.8563	Other-RLP	0.416	(Other-RLP)	0.987	vesicle-associated membrane protein. putative. expressed
AT3G50050.1	Y	Y	RLP	0.8937	RLP	0.8558	Other-RLP	0.3665	(Other-RLP)	0.9867	eukaryotic aspartyl protease domain containing protein. expressed
AT5G42370.1	Y	Y	RLP	0.6338	RLP	0.9905	Other-RLP	0.483	(Other-RLP)	0.9866	Subfamily not named and unknown function
AT1G59970.1	Y	Y	RLP	0.6337	RLP	0.9911	Other-RLP	0.3997	(Other-RLP)	0.9866	metalloendoproteinase 1 precursor. putative. expressed
AT4G23720.1	Y	Y	RLP	0.6818	RLP	0.9901	Other-RLP	0.3495	(Other-RLP)	0.9866	Subfamily not named and unknown function
AT1G66970.1	Y	Y	RLP	0.8952	RLP	0.9901	Other-RLP	0.3996	(Other-RLP)	0.9865	**glycerophosphoryl**

											**diester phosphodiesterase family protein. putative. expressed**
AT5G58050.1	Y	Y	RLP	0.6339	RLP	0.9911	Other-RLP	0.3171	(Other-RLP)	0.9859	**glycerophosphoryl diester phosphodiesterase family protein. putative. expressed**
AT5G55480.1	Y	Y	RLP	0.7973	RLP	0.8751	Other-RLP	0.352	(Other-RLP)	0.9858	**glycerophosphoryl diester phosphodiesterase family protein. putative. expressed**
AT5G46850.1	N	Y	RLP	0.6337	RLP	0.9898	Other-RLP	0.3503	(Other-RLP)	0.8033	Subfamily not named and unknown function
AT5G42860.1	N	Y	RLP	0.8408	RLP	0.9911	Other-RLP	0.4662	(Other-RLP)	0.8032	Subfamily not named and unknown function
AT5G54170.1	N	Y	RLP	0.6817	RLP	0.9901	Other-RLP	0.3671	(Other-RLP)	0.802	membrane related protein CP5. putative. expressed
AT1G14490.1	N	Y	RLP	0.8419	RLP	0.991	Other-RLP	0.4336	(Other-RLP)	0.8009	Subfamily not named and unknown function
AT1G72820.1	N	Y	RLP	0.6787	RLP	0.7161	Other-RLP	0.3667	(Other-RLP)	0.8008	mitochondrial carrier protein. putative. expressed
ATCG00540.1	N	Y	RLP	0.6866	RLP	0.9906	Other-RLP	0.5661	(Other-RLP)	0.8002	apocytochrome f precursor. putative. expressed
AT4G17905.1	N	Y	RLP	0.6841	RLP	0.8584	Other-RLP	0.3333	(Other-RLP)	0.8	**RING-H2 finger protein ATL5G / Other-RLK (Ring finger-RLK)**
AT5G47180.1	N	Y	RLP	0.6861	RLP	0.9895	Other-RLP	0.4828	(Other-RLP)	0.7997	MSP domain containing protein. expressed
AT5G41530.1	N	Y	RLP	0.6812	RLP	0.9903	Other-RLP	0.3337	(Other-RLP)	0.7996	Subfamily not named and unknown function

AT5G63780.1	N	Y	RLP	0.8449	RLP	0.9903	Other-RLP	0.417	(Other-RLP)	0.7992	zinc finger. C3HC4 type domain containing protein. expressed
AT4G25030.1	N	Y	RLP	0.6286	RLP	0.992	Other-RLP	0.4671	(Other-RLP)	0.7991	Subfamily not named and unknown function
AT2G20590.1	N	Y	RLP	0.6826	RLP	0.9912	Other-RLP	0.5665	(Other-RLP)	0.7989	reticulon domain containing protein. putative. expressed
AT3G20270.1	N	Y	RLP	0.7896	RLP	0.8535	Other-RLP	0.3163	(Other-RLP)	0.7989	BPI/LBP family protein At3g20270 precursor. putative. expressed
AT1G74730.1	N	Y	RLP	0.631	RLP	0.9903	Other-RLP	0.3012	(Other-RLP)	0.7989	Subfamily not named and unknown function
AT2G30505.1	N	Y	RLP	0.7873	RLP	0.9898	Other-RLP	0.4996	(Other-RLP)	0.7988	Subfamily not named and unknown function
AT2G33110.1	N	Y	RLP	0.7364	RLP	0.9917	Other-RLP	0.4005	(Other-RLP)	0.7986	vesicle-associated membrane protein. putative. expressed
AT4G22890.1	N	Y	RLP	0.7345	RLP	0.9915	Other-RLP	0.3502	(Other-RLP)	0.7984	Subfamily not named and unknown function
AT1G78880.1	N	Y	RLP	0.8948	RLP	0.9901	Other-RLP	0.4337	(Other-RLP)	0.7977	Subfamily not named and unknown function
AT5G59400.1	N	Y	RLP	0.7376	RLP	0.9901	Other-RLP	0.5168	(Other-RLP)	0.7974	Subfamily not named and unknown function
AT5G63050.1	N	Y	RLP	0.7383	RLP	0.9903	Other-RLP	0.4166	(Other-RLP)	0.7974	Subfamily not named and unknown function
AT1G67540.1	N	Y	RLP	0.6852	RLP	0.9919	Other-RLP	0.3664	(Other-RLP)	0.7973	Subfamily not named and unknown function
AT5G52980.1	N	Y	RLP	0.8419	RLP	0.9918	Other-RLP	0.3996	(Other-RLP)	0.797	Subfamily not named and unknown function
AT1G76490.1	N	Y	RLP	0.6824	RLP	0.9889	Other-RLP	0.5165	(Other-RLP)	0.7963	3-hydroxy-3-methylglutaryl-coenzyme A reductase. putative. expressed
AT2G34380.1	N	Y	RLP	0.6318	RLP	0.9913	Other-RLP	0.3669	(Other-RLP)	0.7951	Subfamily not named and unknown function
AT5G48830.1	N	Y	RLP	0.6317	RLP	0.9908	Other-RLP	0.3667	(Other-RLP)	0.7922	Subfamily not named and unknown function

AT1G48510.1	N	Y	RLP	0.6308	RLP	0.9906	Other-RLP	0.5332	(Other-RLP)	0.7916	SURF1. putative. expressed
AT5G64930.1	N	Y	RLP	0.841	RLP	0.9906	Other-RLP	0.4667	(Other-RLP)	0.7913	Subfamily not named and unknown function
AT4G35170.1	N	Y	RLP	0.7899	RLP	0.9912	Other-RLP	0.3162	(Other-RLP)	0.7986	Subfamily not named and unknown function
AT3G27410.1	Y	Y	RLP	0.996	RLP	0.9911	Ethylene-responsive-RLP	0.3665	(Ethylene-responsive-RLP)	0.9874	Subfamily not named and unknown function
AT2G28440.1	Y	Y	RLP	0.8409	RLP	0.9912	Ethylene-responsive-RLP	0.466	(Ethylene-responsive-RLP)	0.9869	Subfamily not named and unknown function
AT1G30515.1	N	Y	RLP	0.6846	RLP	0.9906	Ethylene-responsive-RLP	0.3163	(Ethylene-responsive-RLP)	0.7923	Subfamily not named and unknown function
AT3G01070.1	Y	Y	RLP	0.6867	RLP	0.9907	Glycosyl-hydrolases-RLP	0.3661	(Glycosyl-hydrolases-RLP)	0.9874	plastocyanin-like domain containing protein / Other-RLP associated with Plastocyanin-like-RLK
AT5G14345.1	Y	Y	RLP	0.735	RLP	0.99	Glycosyl-hydrolases-RLP	0.3342	(Glycosyl-hydrolases-RLP)	0.9873	plastocyanin-like domain containing protein / Other-RLP associated with Plastocyanin-like-RLK
AT2G44520.1	N	Y	RLP	0.6813	RLP	0.9901	Glycosyl-hydrolases-RLP	0.4503	(Glycosyl-hydrolases-RLP)	0.8024	prenyltransferase. putative. expressed
AT1G35880.1	N	Y	RLP	0.844	RLP	0.9909	Glycosyl-hydrolases-RLP	0.3334	(Glycosyl-hydrolases-RLP)	0.8005	Subfamily not named and unknown function
AT5G66450.1	N	Y	RLP	0.7877	RLP	0.9904	Glycosyl-hydrolases-RLP	0.3835	(Glycosyl-hydrolases-RLP)	0.7993	Subfamily not named and unknown function
AT5G57345.1	N	Y	RLP	0.7378	RLP	0.9897	Glycosyl-	0.3327	(Glycosyl-hydrolases-	0.7973	Subfamily not named

							hydrolases-RLP		RLP)		and unknown function
AT5G19750.1	N	Y	RLP	0.682	RLP	0.99	Glycosyl-hydrolases-RLP	0.3833	(Glycosyl-hydrolases-RLP)	0.7963	Mpv17 / PMP22 family domain containing protein. expressed
AT2G39805.1	N	Y	RLP	0.8952	RLP	0.9907	Glycosyl-hydrolases-RLP	0.4001	(Glycosyl-hydrolases-RLP)	0.7949	Yip1 domain containing protein. expressed
AT2G42390.1	Y	Y	RLP	0.6859	RLP	0.992	PAN-RLP	0.334	(PAN-RLP)	0.9879	glucosidase II beta subunit-like domain containing protein. expressed
AT3G03726.1	N	Y	RLP	0.79	RLP	0.9896	PAN-RLP	0.3661	(PAN-RLP)	0.7988	Subfamily not named and unknown function
		Y									
AT3G27200.1	Y	Y	RLP	0.787	RLP	0.9903	RCC1-RLP	0.4167	(RCC1-RLP)	0.989	Subfamily not named and unknown function
AT4G01575.1	Y	Y	RLP	0.8394	RLP	0.991	RCC1-RLP	0.383	(RCC1-RLP)	0.9871	Subfamily not named and unknown function
AT2G01660.1	Y	Y	RLP	0.9969	RLP	0.9906	RCC1-RLP	0.3166	(RCC1-RLP)	0.9869	Salt stress response/antifungal-RLP
AT2G27389.1	Y	Y	RLP	0.7899	RLP	0.9915	RCC1-RLP	0.45	(RCC1-RLP)	0.9867	Subfamily not named and unknown function
AT2G40316.1	Y	Y	RLP	0.9967	RLP	0.9907	RCC1-RLP	0.4007	(RCC1-RLP)	0.9866	Subfamily not named and unknown function
AT1G56200.1	N	Y	RLP	0.8952	RLP	0.9903	RCC1-RLP	0.3168	(RCC1-RLP)	0.8009	Subfamily not named and unknown function
AT1G53640.1	N	Y	RLP	0.6809	RLP	0.9917	RCC1-RLP	0.3501	(RCC1-RLP)	0.7979	Subfamily not named and unknown function
AT3G46240.1	N	Y	RLP	0.7885	RLP	0.9894	RCC1-RLP	0.3338	(RCC1-RLP)	0.7974	Malectin-RLP
AT3G08600.1	Y	Y	RLP	0.7903	RLP	0.9914	Unknown-RLP	0.6003	(Unknown-RLP)	0.9887	**Subfamily not named and unknown function**
AT5G65390.1	Y	Y	RLP	0.9963	RLP	0.9904	Unknown-RLP	0.3665	(Unknown-RLP)	0.9883	**Subfamily not named**

											**and unknown function**
AT3G03860.1	Y	Y	RLP	0.686	RLP	0.9906	Unknown-RLP	0.3338	(Unknown-RLP)	0.9883	OsAPRL5 adenosine 5\'-phosphosulfate reductase-like OsAPRL5. expressed
AT4G08670.1	Y	Y	RLP	0.9965	RLP	0.9911	Unknown-RLP	0.3329	(Unknown-RLP)	0.9881	LTPL66 - Protease inhibitor / Other-RLP associated with Probable-lipid-transfer-RLK
AT4G11950.1	Y	Y	RLP	0.7883	RLP	0.9909	Unknown-RLP	0.4833	(Unknown-RLP)	0.9879	**Subfamily not named and unknown function**
AT1G11130.1	Y	Y	RLP	0.6837	RLP	0.7131	Unknown-RLP	0.3502	(Unknown-RLP)	0.9878	Leucine-rich repeat protein kinase family protein
AT1G23040.1	Y	Y	RLP	0.9485	RLP	0.8575	Unknown-RLP	0.5163	(Unknown-RLP)	0.9877	**Subfamily not named and unknown function**
AT2G47930.1	Y	Y	RLP	0.9964	RLP	0.9896	Unknown-RLP	0.4668	(Unknown-RLP)	0.9877	**Subfamily not named and unknown function**
AT1G35230.1	Y	Y	RLP	0.9961	RLP	0.9904	Unknown-RLP	0.5497	(Unknown-RLP)	0.9876	**Subfamily not named and unknown function**
AT3G27416.1	Y	Y	RLP	0.9966	RLP	0.9909	Unknown-RLP	0.4169	(Unknown-RLP)	0.9875	**Subfamily not named and unknown function**
AT5G10430.1	Y	Y	RLP	0.9452	RLP	0.9907	Unknown-RLP	0.534	(Unknown-RLP)	0.9874	**Subfamily not named and unknown function**
AT5G53870.1	Y	Y	RLP	0.9965	RLP	0.9911	Unknown-RLP	0.5164	(Unknown-RLP)	0.9874	plastocyanin-like domain containing protein / Other-RLP associated with Plastocyanin-like-RLK
AT4G09560.1	Y	Y	RLP	0.8934	RLP	0.991	Unknown-RLP	0.3335	(Unknown-RLP)	0.9872	RING finger protein 13 / Other-RLK (Ring finger-RLK)
AT2G23130.1	Y	Y	RLP	0.996	RLP	0.991	Unknown-RLP	0.6997	(Unknown-RLP)	0.9871	**Subfamily not named and unknown function**
AT4G27520.1	Y	Y	RLP	0.9466	RLP	0.99	Unknown-RLP	0.433	(Unknown-RLP)	0.9871	plastocyanin-like domain containing protein / Other-RLP

											associated with Plastocyanin-like-RLK
AT5G60650.1	Y	Y	RLP	0.8417	RLP	0.856	Unknown-RLP	0.3497	(Unknown-RLP)	0.9871	**Subfamily not named and unknown function**
AT4G34190.1	Y	Y	RLP	0.7873	RLP	0.9905	Unknown-RLP	0.4495	(Unknown-RLP)	0.987	**Subfamily not named and unknown function**
AT3G60280.1	Y	Y	RLP	0.8962	RLP	0.9911	Unknown-RLP	0.3169	(Unknown-RLP)	0.9868	plastocyanin-like domain containing protein / Other-RLP associated with Plastocyanin-like-RLK
AT1G02405.1	Y	Y	RLP	0.9961	RLP	0.9903	Unknown-RLP	0.6669	(Unknown-RLP)	0.9867	**Proline-rich family protein/Subfamily not named and unknown function**
AT1G36150.1	Y	Y	RLP	0.9465	RLP	0.9902	Unknown-RLP	0.4164	(Unknown-RLP)	0.9867	LTPL69 - Protease inhibitor / Other-RLP associated with Probable-lipid-transfer-RLK
AT5G11990.1	Y	Y	RLP	0.9966	RLP	0.9914	Unknown-RLP	0.5335	(Unknown-RLP)	0.9866	**Proline-rich family protein/Subfamily not named and unknown function**
AT4G28100.1	Y	Y	RLP	0.8927	RLP	0.9907	Unknown-RLP	0.3832	(Unknown-RLP)	0.9865	**Subfamily not named and unknown function**
AT4G09030.1	Y	Y	RLP	0.9965	RLP	0.9908	Unknown-RLP	0.6673	(Unknown-RLP)	0.9864	**Subfamily not named and unknown function**
AT5G18690.1	Y	Y	RLP	0.9963	RLP	0.9902	Unknown-RLP	0.3503	(Unknown-RLP)	0.9864	**Subfamily not named and unknown function**
AT3G45275.1	Y	Y	RLP	0.8421	RLP	0.9907	Unknown-RLP	0.4164	(Unknown-RLP)	0.9862	**Subfamily not named and unknown function**
AT2G22470.1	Y	Y	RLP	0.8958	RLP	0.9906	Unknown-RLP	0.4672	(Unknown-RLP)	0.986	**Subfamily not named and unknown function**
AT1G72600.1	Y	Y	RLP	0.8427	RLP	0.9914	Unknown-RLP	0.4997	(Unknown-RLP)	0.9858	**Subfamily not named and unknown function**
AT5G14380.1	Y	Y	RLP	0.8933	RLP	0.9906	Unknown-RLP	0.3833	(Unknown-RLP)	0.9858	**Subfamily not named and unknown function**

AT3G45230.1	Y	Y	RLP	0.8437	RLP	0.9914	Unknown-RLP	0.5834	(Unknown-RLP)	0.9855	**Subfamily not named and unknown function**
AT3G29270.1	N	Y	RLP	0.7359	RLP	0.9904	Unknown-RLP	0.35	(Unknown-RLP)	0.8032	RING zinc finger protein-like / Other-RLK (Ring finger-RLK)
AT2G07678.1	N	Y	RLP	0.7893	RLP	0.8558	Unknown-RLP	0.3661	(Unknown-RLP)	0.7997	**Subfamily not named and unknown function**
AT2G01590.1	N	Y	RLP	0.6804	RLP	0.9905	Unknown-RLP	0.4832	(Unknown-RLP)	0.7993	**Subfamily not named and unknown function**
AT5G06660.1	N	Y	RLP	0.736	RLP	0.9907	Unknown-RLP	0.466	(Unknown-RLP)	0.799	**Subfamily not named and unknown function**
AT1G14345.1	N	Y	RLP	0.6346	RLP	0.9902	Unknown-RLP	0.5664	(Unknown-RLP)	0.7989	**Subfamily not named and unknown function**
AT5G63040.1	N	Y	RLP	0.7899	RLP	0.9919	Unknown-RLP	0.3167	(Unknown-RLP)	0.7985	**Subfamily not named and unknown function**
AT2G20230.1	N	Y	RLP	0.7363	RLP	0.9905	Unknown-RLP	0.3332	(Unknown-RLP)	0.7982	**Subfamily not named and unknown function**
AT4G35080.1	N	Y	RLP	0.6868	RLP	0.9911	Unknown-RLP	0.3838	(Unknown-RLP)	0.7979	high-affinity nickel-transport family protein. putative. expressed
AT4G30260.1	N	Y	RLP	0.7871	RLP	0.9912	Unknown-RLP	0.3666	(Unknown-RLP)	0.7979	Yip1 domain containing protein. expressed
AT3G56010.1	N	Y	RLP	0.7366	RLP	0.99	Unknown-RLP	0.3169	(Unknown-RLP)	0.7979	**Subfamily not named and unknown function**
AT3G12030.1	N	Y	RLP	0.7916	RLP	0.9905	Unknown-RLP	0.4002	(Unknown-RLP)	0.7978	**Subfamily not named and unknown function**
AT4G28770.1	N	Y	RLP	0.9479	RLP	0.9907	Unknown-RLP	0.3338	(Unknown-RLP)	0.7978	**Subfamily not named and unknown function**
AT4G14690.1	N	Y	RLP	0.7387	RLP	0.9913	Unknown-RLP	0.317	(Unknown-RLP)	0.7977	early light-induced protein. chloroplast precursor. putative. expressed
AT3G26350.1	N	Y	RLP	0.6292	RLP	0.9915	Unknown-RLP	0.5831	(Unknown-RLP)	0.7976	harpin-induced protein 1 domain containing protein. expressed

AT4G24460.1	N	Y	RLP	0.7368	RLP	0.9902	Unknown-RLP	0.3169	(Unknown-RLP)	0.7974	**Subfamily not named and unknown function**
AT2G25169.1	N	Y	RLP	0.8403	RLP	0.9905	Unknown-RLP	0.3667	(Unknown-RLP)	0.7973	**Subfamily not named and unknown function**
AT1G44890.1	N	Y	RLP	0.6841	RLP	0.8546	Unknown-RLP	0.3498	(Unknown-RLP)	0.7971	**Subfamily not named and unknown function**
AT3G49840.1	N	Y	RLP	0.7341	RLP	0.991	Unknown-RLP	0.3502	(Unknown-RLP)	0.7967	**Subfamily not named and unknown function**
AT2G38360.1	N	Y	RLP	0.629	RLP	0.9902	Unknown-RLP	0.3174	(Unknown-RLP)	0.7967	prenylated rab acceptor. putative. expressed
AT1G29390.1	N	Y	RLP	0.8953	RLP	0.9913	Unknown-RLP	0.4835	(Unknown-RLP)	0.7966	cold acclimation protein WCOR413. putative. expressed
AT1G54215.1	N	Y	RLP	0.6825	RLP	0.9908	Unknown-RLP	0.6666	(Unknown-RLP)	0.7963	**Subfamily not named and unknown function**
AT2G16800.1	N	Y	RLP	0.8426	RLP	0.9904	Unknown-RLP	0.4	(Unknown-RLP)	0.796	high-affinity nickel-transport family protein. putative. expressed
ATMG00920.1	N	Y	RLP	0.7872	RLP	0.858	Unknown-RLP	0.3839	(Unknown-RLP)	0.7959	**Subfamily not named and unknown function**
AT4G32490.1	N	Y	RLP	0.7914	RLP	0.9892	Unknown-RLP	0.3164	(Unknown-RLP)	0.7959	plastocyanin-like domain containing protein / Other-RLP associated with Plastocyanin-like-RLK
AT4G32600.1	N	Y	RLP	0.7913	RLP	0.9907	Unknown-RLP	0.4002	(Unknown-RLP)	0.7958	zinc finger family protein. putative. expressed
AT5G01080.1	N	Y	RLP	0.6836	RLP	0.9903	Unknown-RLP	0.3334	(Unknown-RLP)	0.7957	**Subfamily not named and unknown function**
AT3G01345.1	N	Y	RLP	0.8439	RLP	0.9904	Unknown-RLP	0.3499	(Unknown-RLP)	0.7951	**Subfamily not named and unknown function**
AT4G04870.1	N	Y	RLP	0.6829	RLP	0.8572	Unknown-RLP	0.3663	(Unknown-RLP)	0.7941	CDP-alcohol phosphatidyltransferase. putative. expressed
AT4G03298.1	N	Y	RLP	0.7313	RLP	0.9896	Unknown-RLP	0.3998	(Unknown-RLP)	0.794	**Subfamily not named and unknown function**

AT4G08874.1	N	Y	RLP	0.6312	RLP	0.991	Unknown-RLP	0.4	(Unknown-RLP)	0.7932	**Subfamily not named and unknown function**
AT4G21740.1	N	Y	RLP	0.9963	RLP	0.9922	Unknown-RLP	0.3004	(Unknown-RLP)	0.7929	**Subfamily not named and unknown function**
AT5G11280.1	N	Y	RLP	0.6307	RLP	0.9893	Unknown-RLP	0.5336	(Unknown-RLP)	0.7928	**Subfamily not named and unknown function**
AT1G16860.1	N	Y	RLP	0.8935	RLP	0.9908	Unknown-RLP	0.3663	(Unknown-RLP)	0.7928	**Subfamily not named and unknown function**
AT1G80200.1	N	Y	RLP	0.7877	RLP	0.9907	Unknown-RLP	0.416	(Unknown-RLP)	0.792	**Subfamily not named and unknown function**
C	Y	Y	RLP	0.8937	RLP	0.9908	Unknown	0.5159	(Undefined)	0.9891	Subfamily not named and unknown function
AT3G16670.1	Y	Y	RLP	0.8393	RLP	0.9893	Unknown	0.283	(Undefined)	0.7975	Subfamily not named and unknown function
AT2G16230.1	Y	Y	RLP	0.6837	RLP	0.9915	Unknown	0.2834	(Undefined)	0.7973	glucan endo-1.3-beta-glucosidase precursor. putative. expressed
AT3G06035.1	Y	Y	RLP	0.9967	RLP	0.9919	Unknown	0.2661	(Undefined)	0.7959	Subfamily not named and unknown function
AT1G62790.1	Y	Y	RLP	0.738	RLP	0.9912	Unknown	0.2662	(Undefined)	0.7921	LTPL85 - Protease inhibitor / Other-RLP associated with Probable-lipid-transfer-RLK
AT5G50050.1	Y	Y	RLP	0.6851	RLP	0.9904	Unknown	0.2329	(Undefined)	0.8001	Plant invertase/pectin methylesterase inhibitor superfamily protein
AT1G43090.1	Y	Y	RLP	0.7367	RLP	0.9903	Unknown	0.4498	(Undefined)	0.9885	polygalacturonase. putative. expressed
AT4G26466.1	Y	Y	RLP	0.8421	RLP	0.9897	Unknown	0.3165	(Undefined)	0.9884	Subfamily not named and unknown function
AT1G71696.1	Y	Y	RLP	0.6811	RLP	0.9899	Unknown	0.3001	(Undefined)	0.9884	Subfamily not named and unknown function
AT4G36945.1	Y	Y	RLP	0.9966	RLP	0.9912	Unknown	0.3836	(Undefined)	0.988	Subfamily not named and unknown function
AT2G01630.1	Y	Y	RLP	0.6316	RLP	0.9903	Unknown	0.333	(Undefined)	0.9879	glucan endo-1.3-beta-glucosidase precursor.

											putative. expressed
AT2G19060.1	Y	Y	RLP	0.682	RLP	0.9896	Unknown	0.3672	(Undefined)	0.9878	GDSL-like lipase/acylhydrolase. putative. expressed
AT5G49270.1	Y	Y	RLP	0.7377	RLP	0.9918	Unknown	0.3502	(Undefined)	0.9877	COBRA-like protein 7 precursor. putative. expressed
AT5G48750.1	Y	Y	RLP	0.7349	RLP	0.9901	Unknown	0.3333	(Undefined)	0.9875	Subfamily not named and unknown function
AT3G18590.1	Y	Y	RLP	0.8457	RLP	0.9908	Unknown	0.3167	(Undefined)	0.9875	plastocyanin-like domain containing protein / Other-RLP associated with Probable-lipid-transfer-RLK
AT3G15720.1	Y	Y	RLP	0.688	RLP	0.991	Unknown	0.4662	(Undefined)	0.9874	polygalacturonase. putative. expressed
AT2G15770.1	Y	Y	RLP	0.9455	RLP	0.9905	Unknown	0.3167	(Undefined)	0.9874	plastocyanin-like domain containing protein / Other-RLP associated with Probable-lipid-transfer-RLK
AT1G71980.1	Y	Y	RLP	0.7903	RLP	0.9901	Unknown	0.3	(Undefined)	0.9874	RING finger protein 13. putative / Other-RLK (Ring finger-RLK)
AT3G13560.1	Y	Y	RLP	0.6873	RLP	0.9904	Unknown	0.3502	(Undefined)	0.9873	glucan endo-1.3-beta-glucosidase precursor. putative. expressed
AT1G74790.1	Y	Y	RLP	0.9469	RLP	0.9909	Unknown	0.333	(Undefined)	0.9873	expressed protein
AT5G42100.1	Y	Y	RLP	0.7381	RLP	0.9898	Unknown	0.3831	(Undefined)	0.9872	glycosyl hydrolases family 17. putative. expressed
AT1G43100.1	Y	Y	RLP	0.6837	RLP	0.9898	Unknown	0.5506	(Undefined)	0.987	polygalacturonase. putative. expressed
AT4G36440.1	Y	Y	RLP	0.7391	RLP	0.854	Unknown	0.5169	(Undefined)	0.987	Subfamily not named and unknown function

AT4G20790.1	Y	Y	RLP	0.9473	RLP	0.7125	Unknown	0.4673	(Undefined)	0.9868	Leucine-rich repeat protein kinase family protein
AT1G09790.1	Y	Y	RLP	0.7891	RLP	0.8569	Unknown	0.3668	(Undefined)	0.9868	COBRA. putative. expressed
AT2G17760.1	Y	Y	RLP	0.7893	RLP	0.8587	Unknown	0.5002	(Undefined)	0.9866	eukaryotic aspartyl protease domain containing protein. expressed
AT1G65720.1	Y	Y	RLP	0.8959	RLP	0.9899	Unknown	0.3163	(Undefined)	0.9866	Subfamily not named and unknown function
AT3G13410.1	Y	Y	RLP	0.789	RLP	0.9903	Unknown	0.3501	(Undefined)	0.9865	Subfamily not named and unknown function
AT1G22670.1	Y	Y	RLP	0.8951	RLP	0.9899	Unknown	0.3498	(Undefined)	0.9865	RING finger protein 13. putative / Other-RLK (Ring finger-RLK)
AT4G33490.1	Y	Y	RLP	0.6828	RLP	0.8551	Unknown	0.4167	(Undefined)	0.9864	eukaryotic aspartyl protease domain containing protein. expressed
AT2G25410.1	Y	Y	RLP	0.9959	RLP	0.9908	Unknown	0.4165	(Undefined)	0.9864	RING-H2 finger protein ATL2M / Other-RLK (Ring finger-RLK)
AT5G36001.1	Y	Y	RLP	0.9964	RLP	0.9912	Unknown	0.3667	(Undefined)	0.9862	zinc finger. C3HC4 type domain containing protein. expressed
AT3G28720.1	Y	Y	RLP	0.6305	RLP	0.9909	Unknown	0.4169	(Undefined)	0.9854	Subfamily not named and unknown function
AT4G34480.1	Y	Y	RLP	0.6837	RLP	0.9906	Unknown	0.267	(Undefined)	0.8062	glucan endo-1.3-beta-glucosidase precursor. putative. expressed
AT3G52640.1	Y	Y	RLP	0.7366	RLP	0.9907	Unknown	0.2998	(Undefined)	0.8046	nicalin. putative. expressed
AT2G38195.1	N	Y	RLP	0.7353	RLP	0.8595	Unknown	0.3169	(Undefined)	0.8023	Subfamily not named and unknown function
AT3G29810.1	Y	Y	RLP	0.7372	RLP	0.9904	Unknown	0.2343	(Undefined)	0.8016	COBRA. putative. expressed

AT4G16140.1	Y	Y	RLP	0.8414	RLP	0.9907	Unknown	0.2003	(Undefined)	0.8015	Subfamily not named and unknown function
AT1G26510.1	N	Y	RLP	0.6856	RLP	0.9913	Unknown	0.4834	(Undefined)	0.8004	OsFBX440 - F-box domain containing protein. expressed
AT2G11005.1	N	Y	RLP	0.8923	RLP	0.9917	Unknown	0.3168	(Undefined)	0.8004	Subfamily not named and unknown function
AT2G15910.1	N	Y	RLP	0.8941	RLP	0.9905	Unknown	0.3001	(Undefined)	0.7996	expp1 protein precursor. putative. expressed
AT2G36100.1	N	Y	RLP	0.6828	RLP	0.9891	Unknown	0.3501	(Undefined)	0.7992	Subfamily not named and unknown function
AT3G51040.1	N	Y	RLP	0.6309	RLP	0.9907	Unknown	0.3006	(Undefined)	0.7974	green ripe-like. putative. expressed
AT5G40990.1	N	Y	RLP	0.7878	RLP	0.9907	Unknown	0.3001	(Undefined)	0.7972	GDSL-like lipase/acylhydrolase. putative. expressed
AT1G69330.1	N	Y	RLP	0.9961	RLP	0.9899	Unknown	0.3661	(Undefined)	0.7969	RING zinc finger protein-like / Other-RLK (Ring finger-RLK)
AT3G15800.1	Y	Y	RLP	0.6867	RLP	0.9899	Unknown	0.25	(Undefined)	0.7969	glycosyl hydrolases family 17. putative. expressed
AT3G20610.1	N	Y	RLP	0.6334	RLP	0.9904	Unknown	0.3662	(Undefined)	0.7967	non-race specific disease resistance protein/Subfamily not named and unknown function
AT1G80400.1	N	Y	RLP	0.8944	RLP	0.9909	Unknown	0.3993	(Undefined)	0.7965	zinc finger family protein. putative. expressed
AT2G36670.1	Y	Y	RLP	0.63	RLP	0.9909	Unknown	0.2999	(Undefined)	0.7964	Xylanase inhibitor C-terminal
AT4G22650.1	Y	Y	RLP	0.6828	RLP	0.9905	Unknown	0.2334	(Undefined)	0.7953	Subfamily not named and unknown function
AT4G39840.1	Y	Y	RLP	0.9478	RLP	0.9898	Unknown	0.2499	(Undefined)	0.7949	Subfamily not named and unknown function

AT4G22630.1	Y	Y	RLP	0.946	RLP	0.9906	Unknown	0.2501	(Undefined)	0.7947	Subfamily not named and unknown function
AT5G46370.1	N	Y	RLP	0.6819	RLP	0.8558	Unknown	0.3661	(Undefined)	0.7943	potassium channel protein. putative. expressed
AT3G62280.1	Y	Y	RLP	0.8399	RLP	0.9898	Unknown	0.25	(Undefined)	0.7937	GDSL-like lipase/acylhydrolase. putative. expressed
AT1G16360.1	N	Y	RLP	0.8429	RLP	0.9896	Unknown	0.3497	(Undefined)	0.7916	cell cycle control protein. putative. expressed
AT2G30933.1	Y	Y	RLP	0.6858	RLP	0.9916	Unknown	0.4672	(Undefined)	0.9867	X8 domain containing protein. expressed
AT2G44790.1	Y	Y	RLP	0.6334	RLP	0.992	Unknown	0.2001	(Undefined)	0.8006	plastocyanin-like domain containing protein / Other-RLP associated with Plastocyanin-like-RLK
AT2G03505.1	Y	Y	RLP	0.7906	RLP	0.9895	Unknown	0.3	(Undefined)	0.7991	X8 domain containing protein. expressed
AT1G21090.1	Y	Y	RLP	0.9466	RLP	0.9906	Unknown	0.2843	(Undefined)	0.7994	Subfamily not named and unknown function
AT5G49280.1	Y	Y	RLP	0.9964	RLP	0.9909	Unknown	0.2333	(Undefined)	0.7974	Subfamily not named and unknown function
AT1G07690.1	Y	Y	RLP	0.8407	RLP	0.9899	Unknown	0.2338	(Undefined)	0.7954	Subfamily not named and unknown function
AT4G01140.1	Y	Y	RLP	0.896	RLP	0.9903	Unknown	0.2833	(Undefined)	0.7976	Subfamily not named and unknown function
AT5G07190.1	Y	Y	RLP	0.8412	RLP	0.9905	Unknown	0.2502	(Undefined)	0.7962	embryo-specific 3. putative. expressed
AT5G60630.1	Y	Y	RLP	0.6846	RLP	0.9909	Unknown	0.2332	(Undefined)	0.7981	Subfamily not named and unknown function
AT1G71110.1	Y	Y	RLP	0.7383	RLP	0.9913	Unknown	0.2667	(Undefined)	0.7978	Subfamily not named and unknown function
AT2G12400.1	Y	Y	RLP	0.7394	RLP	0.9908	Unknown	0.2333	(Undefined)	0.7977	Subfamily not named and unknown function
AT2G25270.1	Y	Y	RLP	0.7904	RLP	0.9908	Unknown	0.2669	(Undefined)	0.7985	Subfamily not named and unknown function

AT4G31370.1	Y	Y	RLP	0.893	RLP	0.9918	Unknown	0.2666	(Undefined)	0.8001	fasciclin-like arabinogalactan precursor protein / Other-RLP associated with Plastocyanin-like-RLK
AT3G58100.1	Y	Y	RLP	0.6331	RLP	0.9913	Unknown	0.25	(Undefined)	0.8008	X8 domain containing protein. expressed
AT3G51510.1	Y	Y	RLP	0.6313	RLP	0.9897	Unknown	0.2669	(Undefined)	0.8007	Subfamily not named and unknown function
AT5G27830.1	Y	Y	RLP	0.7906	RLP	0.9911	Unknown	0.2835	(Undefined)	0.8005	CRP12 - Cysteine-rich family protein precursor. expressed
AT5G07475.1	Y	Y	RLP	0.8955	RLP	0.9905	Unknown	0.2008	(Undefined)	0.8031	plastocyanin-like domain containing protein / Other-RLP associated with Plastocyanin-like-RLK
AT3G43720.1	Y	Y	RLP	0.7375	RLP	0.9911	Unknown	0.2992	(Undefined)	0.7985	LTPL67 - Protease inhibitor / Other-RLP associated with Probable-lipid-transfer-RLK
AT2G13820.1	Y	Y	RLP	0.8407	RLP	0.9908	Unknown	0.2335	(Undefined)	0.8024	LTPL69 - Protease inhibitor / Other-RLP associated with Probable-lipid-transfer-RLK
AT1G09176.1	Y	Y	RLP	0.8432	RLP	0.9911	Unknown	0.2666	(Undefined)	0.801	Subfamily not named and unknown function
AT4G22900.1	Y	Y	RLP	0.842	RLP	0.991	Unknown	0.2839	(Undefined)	0.8026	Subfamily not named and unknown function
AT1G11362.1	Y	Y	RLP	0.8391	RLP	0.9911	Unknown	0.2502	(Undefined)	0.8002	Subfamily not named and unknown function
AT5G19250.1	Y	Y	RLP	0.9963	RLP	0.9908	Unknown	0.2501	(Undefined)	0.7944	Subfamily not named and unknown function

Table S3. Enriched GO terms in three categories, Biological Process, Molecular Function, or Cellular Component ontology

Celular component						
GOCCID	**Pvalue**	**OddsRatio**	**ExpCount**	**Count**	**Size**	**Term**
GO:0046658	0.00	48.75	0.207	8	68	anchored component of plasma membrane
GO:0044459	0.00	13.09	0.699	8	230	plasma membrane part
GO:0005886	0.00	3.68	8.324	23	3032	plasma membrane
GO:0048046	0.00	7.77	0.997	7	328	apoplast
GO:0005794	0.00	4.43	2.821	11	928	Golgi apparatus
GO:0009505	0.00	7.89	0.833	6	274	plant-type cell wall
GO:0009506	0.00	4.33	2.590	10	852	plasmodesma
GO:0030054	0.00	4.32	2.596	10	854	cell junction
GO:0030312	0.00	4.27	1.782	7	586	external encapsulating structure
GO:0005783	0.01	3.87	1.660	6	546	endoplasmic reticulum
GO:0030173	0.01	110.75	0.012	1	4	integral component of Golgi membrane
GO:0031225	0.01	6.44	0.491	3	180	anchored component of membrane
GO:0005773	0.02	2.87	2.605	7	857	vacuole
GO:0005737	0.04	1.56	36.641	45	12052	cytoplasm
GO:0008180	0.04	27.68	0.040	1	13	COP9 signalosome
GO:0030176	0.04	27.68	0.040	1	13	integral component of endoplasmic reticulum membrane
GO:0098791	0.04	4.04	0.772	3	254	Golgi subcompartment
GO:0098588	0.04	2.74	1.915	5	630	bounding membrane of organelle

GO:0005623	0.05	2.23	65.718	71	21616	cell
GO:0055028	0.05	22.14	0.049	1	16	cortical microtubule

Molecular function

GOMFID	**Pvalue**	**OddsRatio**	**ExpCount**	**Count**	**Size**	**Term**
GO:0008889	1.23E-12	301.83	0.041	6	13	glycerophosphodiester phosphodiesterase activity
GO:0042578	4.89E-09	13.25	1.018	11	326	phosphoric ester hydrolase activity
GO:0045309	1.03E-05	91.30	0.044	3	14	protein phosphorylated amino acid binding
GO:1901981	0.001	54.89	0.044	2	14	phosphatidylinositol phosphate binding
GO:0016301	0.001	3.25	3.870	11	1259	kinase activity
GO:0004127	0.003	Inf	0.003	1	1	cytidylate kinase activity
GO:0004140	0.003	Inf	0.003	1	1	dephospho-CoA kinase activity
GO:0005545	0.003	Inf	0.003	1	1	1-phosphatidylinositol binding
GO:0031210	0.003	Inf	0.003	1	1	phosphatidylcholine binding
GO:0042973	0.003	Inf	0.003	1	1	glucan endo-1.3-beta-D-glucosidase activity
GO:0016787	0.004	2.445	7.145	15	2288	hydrolase activity
GO:0003993	0.008	16.444	0.131	2	42	acid phosphatase activity
GO:0008017	0.008	16.042	0.134	2	43	microtubule binding
GO:0009041	0.009	162.135	0.009	1	3	uridylate kinase activity
GO:0046577	0.012	108.085	0.012	1	4	long-chain-alcohol oxidase activity
GO:0030554	0.014	3.780	1.415	5	453	adenyl nucleotide binding
GO:0005460	0.016	81.060	0.016	1	5	UDP-glucose transmembrane transporter activity
GO:0005546	0.016	81.060	0.016	1	5	phosphatidylinositol-4.5-bisphosphate binding
GO:0050378	0.016	81.060	0.016	1	5	UDP-glucuronate 4-epimerase activity

GO:0000062	0.019	64.844	0.019	1	6	fatty-acyl-CoA binding
GO:0004605	0.025	46.313	0.025	1	8	phosphatidate cytidylyltransferase activity
GO:0008195	0.028	40.522	0.028	1	9	phosphatidate phosphatase activity
GO:0005543	0.029	7.848	0.267	2	87	phospholipid binding
GO:0004630	0.037	29.466	0.037	1	12	phospholipase D activity
GO:0032555	0.041	2.782	1.902	5	609	purine ribonucleotide binding
GO:0005338	0.043	24.930	0.044	1	14	nucleotide-sugar transmembrane transporter activity
GO:0008092	0.044	6.245	0.334	2	107	cytoskeletal protein binding
GO:0097367	0.045	2.708	1.952	5	625	carbohydrate derivative binding
GO:0000166	0.048	2.419	2.636	6	844	nucleotide binding
GO:0005524	0.049	3.050	1.377	4	441	ATP binding

**Biological process**						
GOBPID	**Pvalue**	**OddsRatio**	**ExpCount**	**Count**	**Size**	**Term**
GO:0006071	2.05E-11	162.45	0.06	6	18	glycerol metabolic process
GO:0019751	1.65E-10	105.43	0.08	6	25	polyol metabolic process
GO:0010015	3.62E-08	9.44	1.54	12	458	root morphogenesis
GO:0048765	1.19E-07	10.91	1.08	10	323	root hair cell differentiation
GO:0048469	1.23E-07	10.88	1.09	10	324	cell maturation
GO:0010054	2.78E-07	9.91	1.19	10	354	trichoblast differentiation
GO:0022622	9.83E-07	6.84	2.09	12	623	root system development
GO:0044262	1.94E-06	6.39	2.23	12	665	cellular carbohydrate metabolic process
GO:0071695	6.44E-	6.88	1.68	10	502	anatomical structure maturation

	06					
GO:0010442	1.11E-05	Inf	0.01	2	2	guard cell morphogenesis
GO:0048589	1.21E-05	5.29	2.67	12	808	developmental growth
GO:0009832	2.35E-05	11.73	0.57	6	171	plant-type cell wall biogenesis
GO:0090627	2.45E-05	7.61	1.19	8	366	plant epidermal cell differentiation
GO:0090558	3.69E-05	8.57	0.92	7	286	plant epidermis development
GO:1901615	4.17E-05	5.00	2.55	11	759	organic hydroxy compound metabolic process
GO:0031110	0.000	152.96	0.02	2	6	regulation of microtubule polymerization or depolymerization
GO:0032886	0.000	122.36	0.02	2	7	regulation of microtubule-based process
GO:0048767	0.000	9.80	0.56	5	172	root hair elongation
GO:0010052	0.000	87.39	0.03	2	9	guard cell differentiation
GO:0000902	0.001	4.33	2.33	9	695	cell morphogenesis
GO:0099402	0.001	3.49	3.60	11	1119	plant organ development
GO:0048768	0.001	43.68	0.05	2	16	root hair cell tip growth
GO:0006073	0.001	8.95	0.48	4	149	cellular glucan metabolic process
GO:0090066	0.002	7.68	0.56	4	167	regulation of anatomical structure size
GO:0016049	0.003	3.63	2.42	8	722	cell growth
GO:0052541	0.003	27.79	0.08	2	24	plant-type cell wall cellulose metabolic process
GO:0009267	0.003	5.46	0.99	5	294	cellular response to starvation
GO:0009605	0.003	2.49	6.95	15	2072	response to external stimulus
GO:0080184	0.003	Inf	0.00	1	1	response to phenylpropanoid
GO:0006650	0.004	6.87	0.62	4	186	glycerophospholipid metabolic process
GO:0007275	0.004	2.17	11.57	21	3451	multicellular organism development

GO:0045017	0.004	6.68	0.64	4	191	glycerolipid biosynthetic process
GO:0030243	0.005	9.64	0.33	3	102	cellulose metabolic process
GO:0009932	0.005	4.97	1.08	5	332	cell tip growth
GO:0006661	0.005	9.18	0.35	3	104	phosphatidylinositol biosynthetic process
GO:0031667	0.005	4.77	1.12	5	335	response to nutrient levels
GO:0009816	0.006	18.51	0.12	2	35	defense response to bacterium. incompatible interaction
GO:0031668	0.006	4.58	1.17	5	349	cellular response to extracellular stimulus
GO:0031115	0.007	301.52	0.01	1	2	negative regulation of microtubule polymerization
GO:0009653	0.008	2.53	4.85	11	1512	anatomical structure morphogenesis
GO:0019637	0.008	2.80	3.53	9	1078	organophosphate metabolic process
GO:0052546	0.008	15.66	0.14	2	41	cell wall pectin metabolic process
GO:0044281	0.009	2.11	9.27	17	2765	small molecule metabolic process
GO:0048527	0.009	7.53	0.42	3	126	lateral root development
GO:0008654	0.010	4.11	1.30	5	387	phospholipid biosynthetic process
GO:0009173	0.010	150.75	0.01	1	3	pyrimidine ribonucleoside monophosphate metabolic process
GO:0000272	0.011	13.27	0.16	2	48	polysaccharide catabolic process
GO:0051493	0.013	104.51	0.01	1	4	regulation of cytoskeleton organization
GO:0010971	0.013	100.50	0.01	1	4	positive regulation of G2/M transition of mitotic cell cycle
GO:0030002	0.013	100.50	0.01	1	4	cellular anion homeostasis
GO:0030643	0.013	100.50	0.01	1	4	cellular phosphate ion homeostasis
GO:0080186	0.013	100.50	0.01	1	4	developmental vegetative growth
GO:1901989	0.013	100.50	0.01	1	4	positive regulation of cell cycle phase transition
GO:0046467	0.014	6.29	0.50	3	150	membrane lipid biosynthetic process
GO:0010393	0.015	11.30	0.19	2	56	galacturonan metabolic process
GO:0008361	0.016	11.10	0.19	2	57	regulation of cell size

GO:0044255	0.016	2.48	3.95	9	1177	cellular lipid metabolic process
GO:0000338	0.017	75.37	0.02	1	5	protein deneddylation
GO:0016126	0.018	5.74	0.55	3	164	sterol biosynthetic process
GO:0090626	0.018	4.23	1.00	4	298	plant epidermis morphogenesis
GO:0071555	0.018	5.67	0.56	3	179	cell wall organization
GO:0010026	0.019	5.57	0.57	3	169	trichome differentiation
GO:0010075	0.020	5.50	0.57	3	171	regulation of meristem growth
GO:0010387	0.020	60.29	0.02	1	6	COP9 signalosome assembly
GO:0045931	0.020	60.29	0.02	1	6	positive regulation of mitotic cell cycle
GO:0009664	0.021	4.04	1.04	4	320	plant-type cell wall organization
GO:0010044	0.023	50.24	0.02	1	7	response to aluminum ion
GO:0032271	0.026	44.13	0.03	1	8	regulation of protein polymerization
GO:0015937	0.027	43.06	0.03	1	8	coenzyme A biosynthetic process
GO:0006950	0.027	1.76	12.99	20	3873	response to stress
GO:1902903	0.029	38.61	0.03	1	9	regulation of supramolecular fiber organization
GO:0031333	0.030	37.68	0.03	1	9	negative regulation of protein complex assembly
GO:0090696	0.035	3.41	1.23	4	367	post-embryonic plant organ development
GO:0006796	0.035	2.00	6.01	11	1836	phosphate-containing compound metabolic process
GO:0043647	0.036	7.00	0.30	2	89	inositol phosphate metabolic process
GO:0016043	0.036	1.78	10.01	16	2986	cellular component organization
GO:0051494	0.036	30.14	0.04	1	11	negative regulation of cytoskeleton organization
GO:0007155	0.038	6.77	0.31	2	92	cell adhesion
GO:0010035	0.038	2.11	4.58	9	1367	response to inorganic substance
GO:0010374	0.039	4.21	0.74	3	222	stomatal complex development
GO:0033875	0.040	6.62	0.32	2	94	ribonucleoside bisphosphate metabolic process

GO:0034032	0.040	6.62	0.32	2	94	purine nucleoside bisphosphate metabolic process
GO:0000271	0.042	2.76	1.91	5	570	polysaccharide biosynthetic process
GO:0005982	0.044	4.01	0.78	3	233	starch metabolic process
GO:0019375	0.044	6.28	0.33	2	99	galactolipid biosynthetic process
GO:0055083	0.046	23.18	0.05	1	14	monovalent inorganic anion homeostasis
GO:0072506	0.046	23.18	0.05	1	14	trivalent inorganic anion homeostasis
GO:0009926	0.046	6.09	0.34	2	102	auxin polar transport
GO:0045010	0.047	6.03	0.35	2	103	actin nucleation
GO:0032273	0.048	5.97	0.35	2	104	positive regulation of protein polymerization
GO:0008202	0.049	3.82	0.82	3	244	steroid metabolic process
GO:0072505	0.049	21.52	0.05	1	15	divalent inorganic anion homeostasis
GO:0044089	0.049	5.85	0.36	2	106	positive regulation of cellular component biogenesis
GO:0051495	0.049	5.85	0.36	2	106	positive regulation of cytoskeleton organization
GO:1902905	0.049	5.85	0.36	2	106	positive regulation of supramolecular fiber organization
